# Supplementary material for: Rad53 checkpoint kinase regulation of DNA replication fork rate via Mrc1 phosphorylation
Source: eLife. 2021 Aug 13;10:e69726. doi: 10.7554/eLife.69726 (PMC8387023; doi:10.7554/eLife.69726)

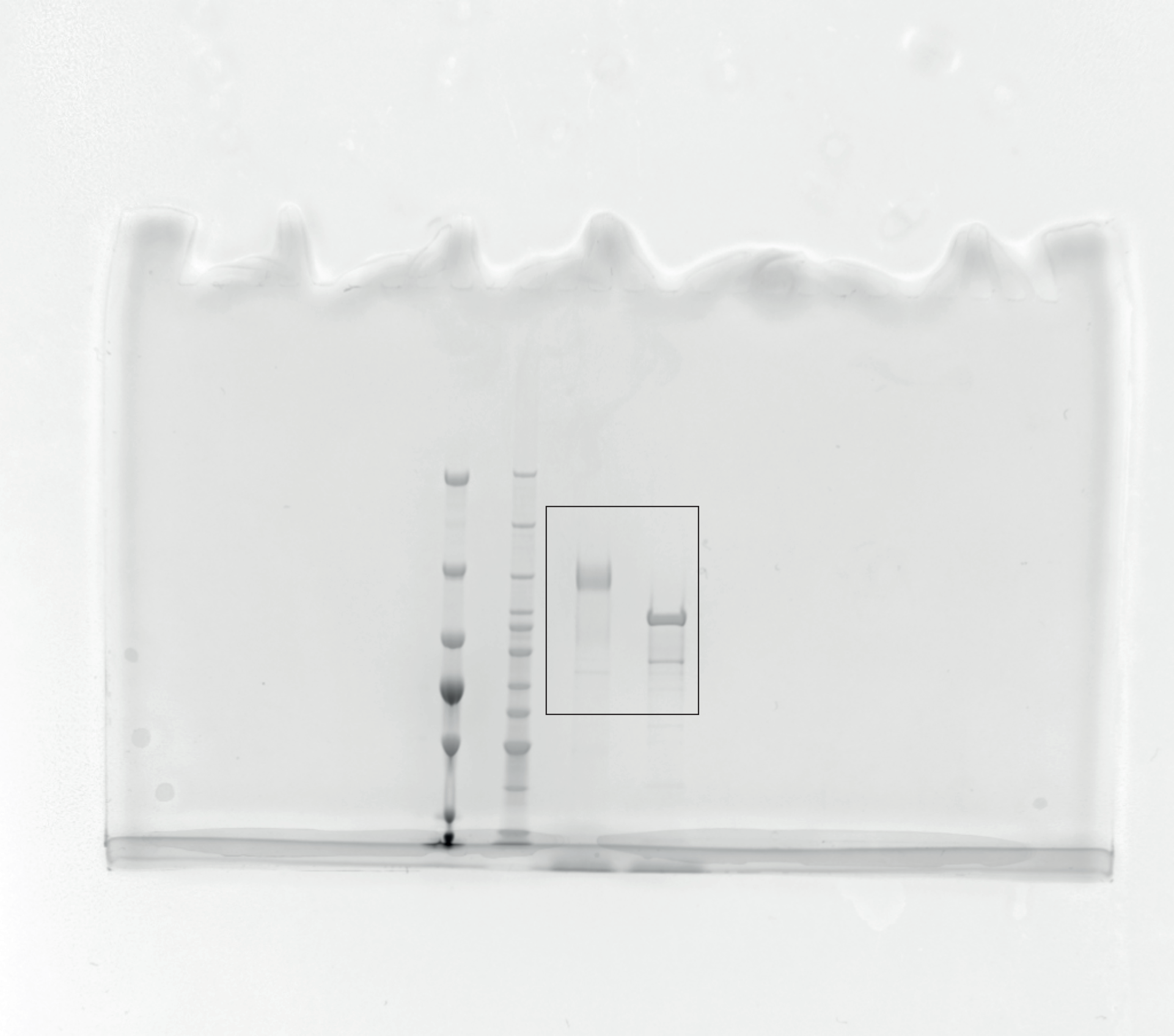

cropped area for Figure 1 - figure supplement 1A

Figure 1 - figure supplement 1 - source data 1.pdf
2048 x 2816

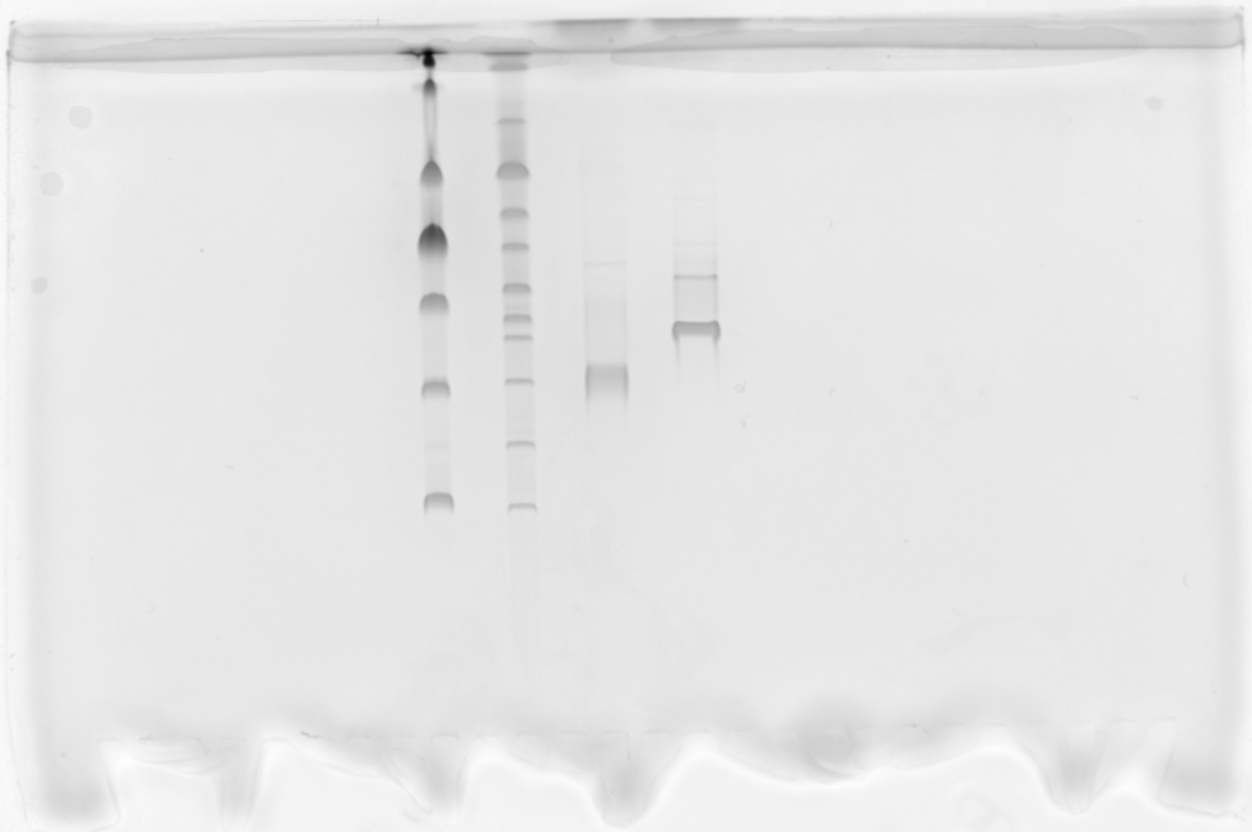

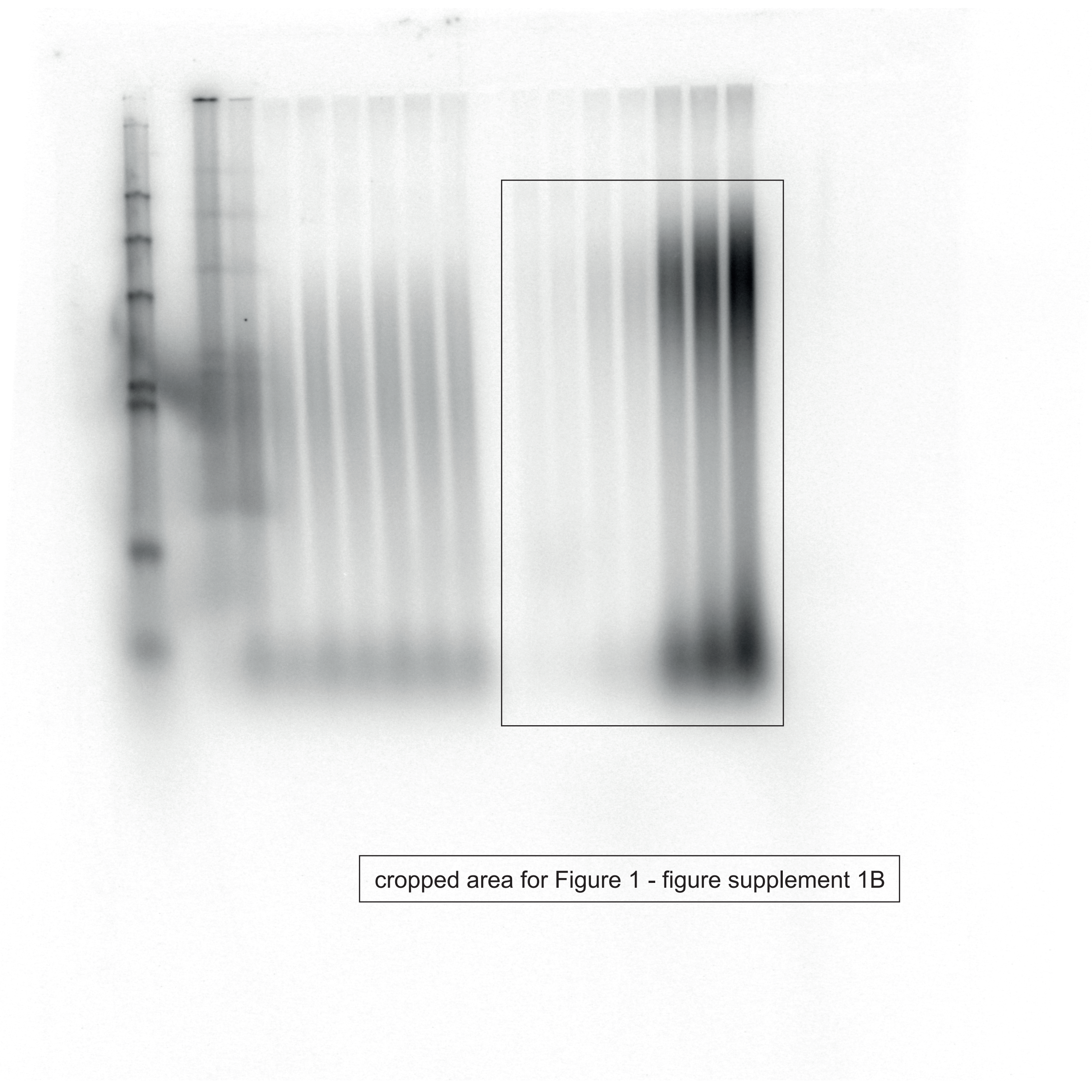

cropped area for Figure 1 - figure supplement 1B

Figure 1 - figure supplement 1 - source data 2.pdf
5000 x 4000

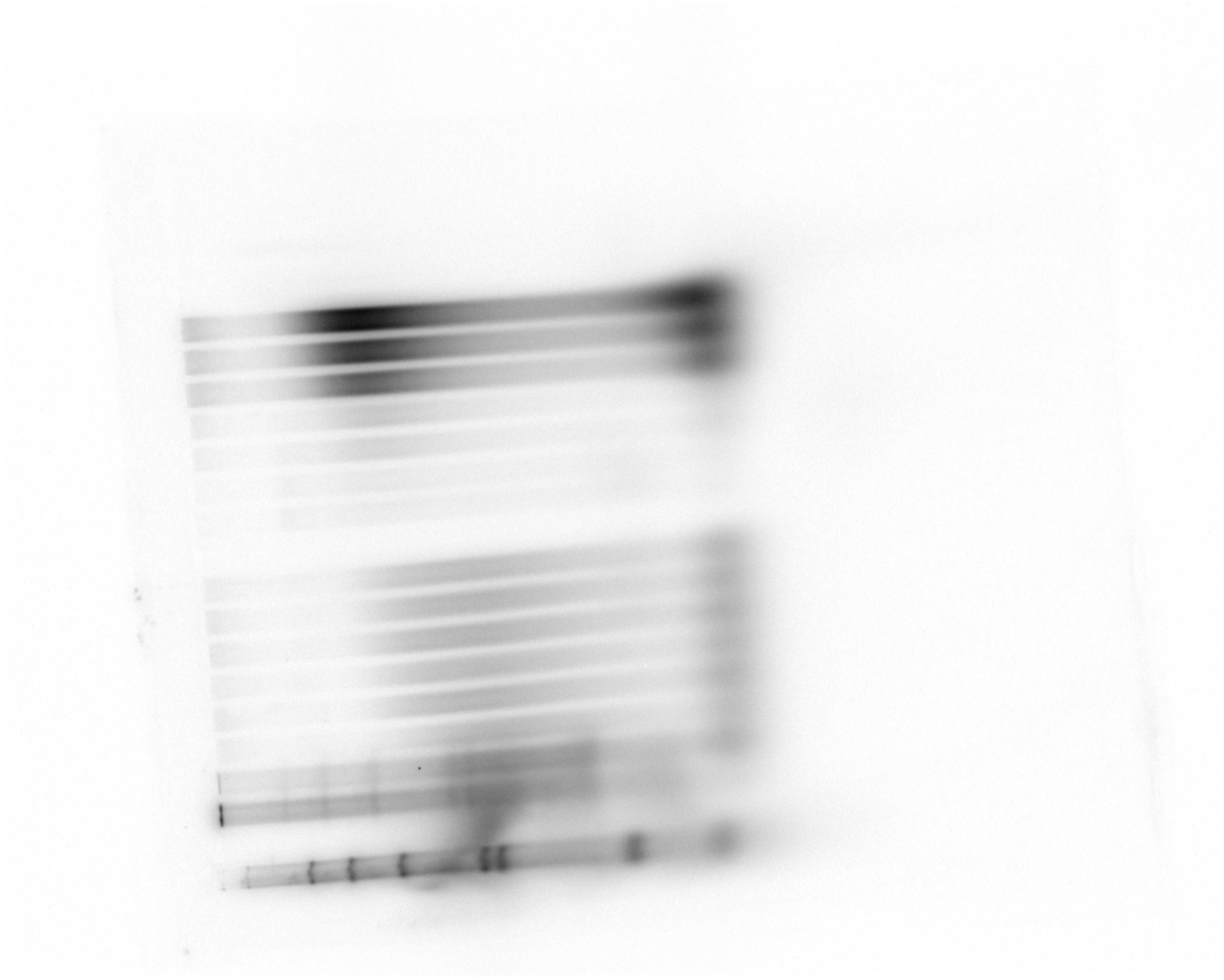

Supplement: Figure 1—figure supplement 1—source data 1. [file elife-69726-fig1-figsupp1-data1.pdf]
