# Supplementary figures and images for: Rad53 checkpoint kinase regulation of DNA replication fork rate via Mrc1 phosphorylation
Source: eLife. 2021 Aug 13;10:e69726. doi: 10.7554/eLife.69726 (PMC8387023; doi:10.7554/eLife.69726)

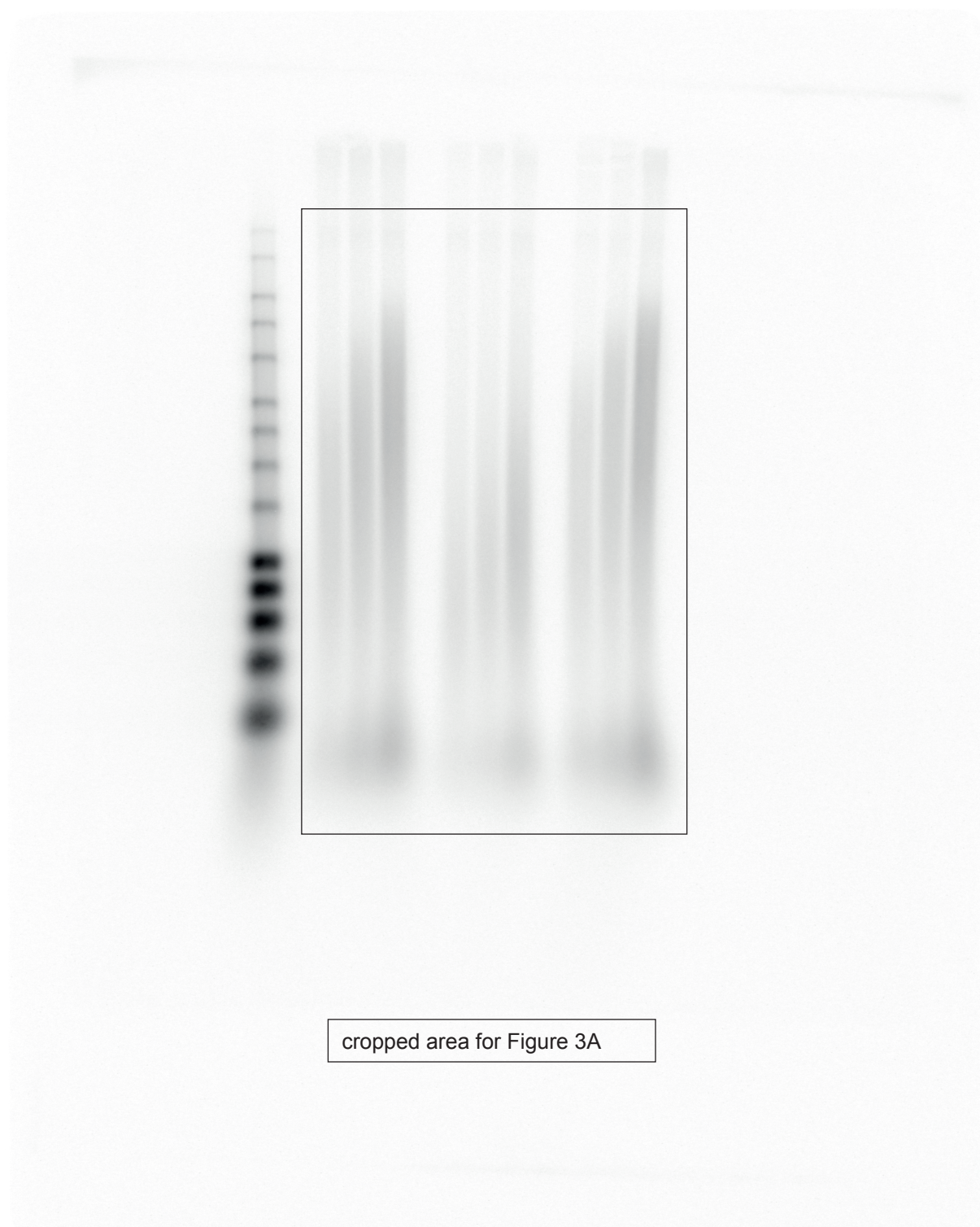

---

Figure 3 - source data 1.pdf  
5000 x 4000

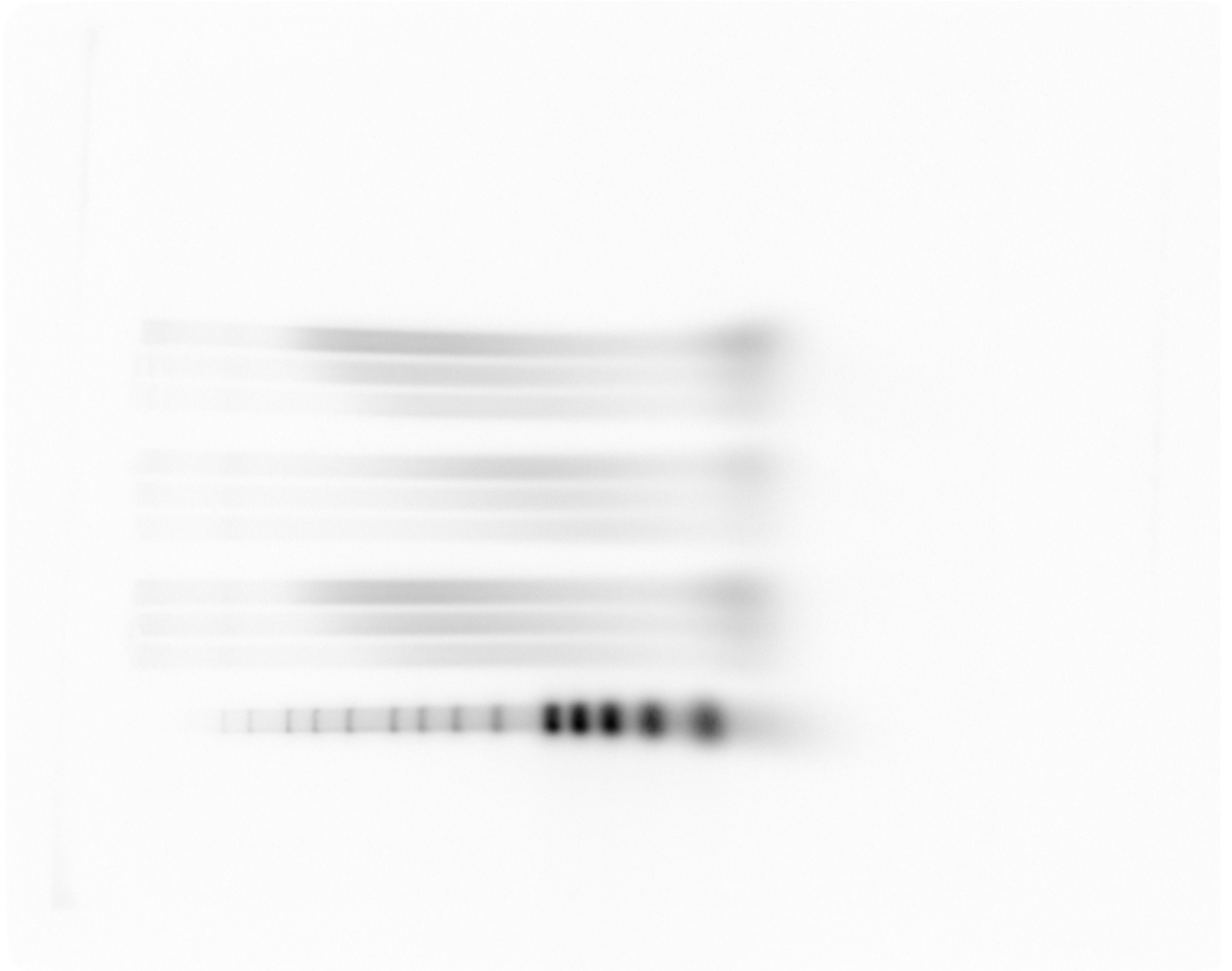

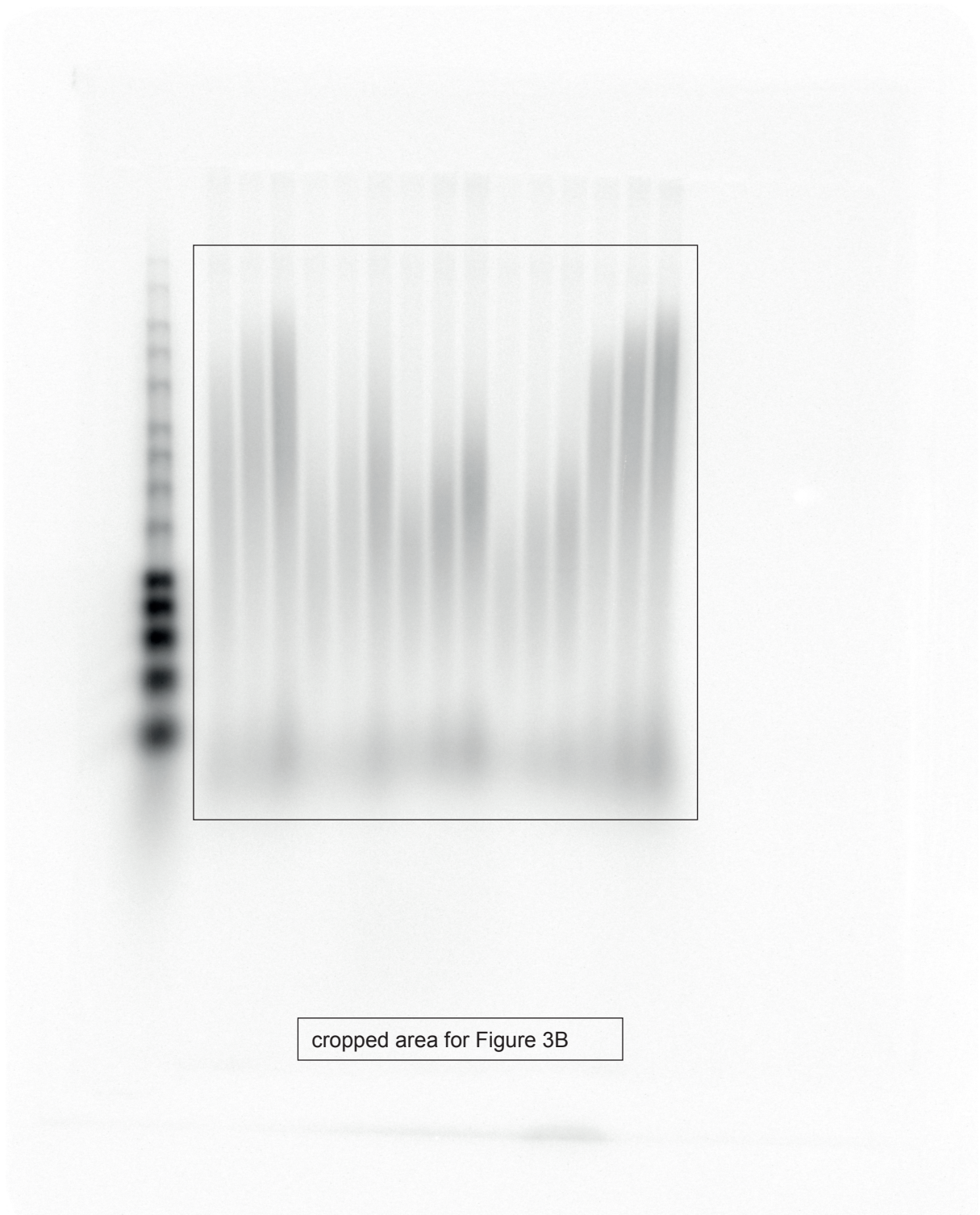

---

Figure 3 - source data 2.pdf  
5000 x 4000

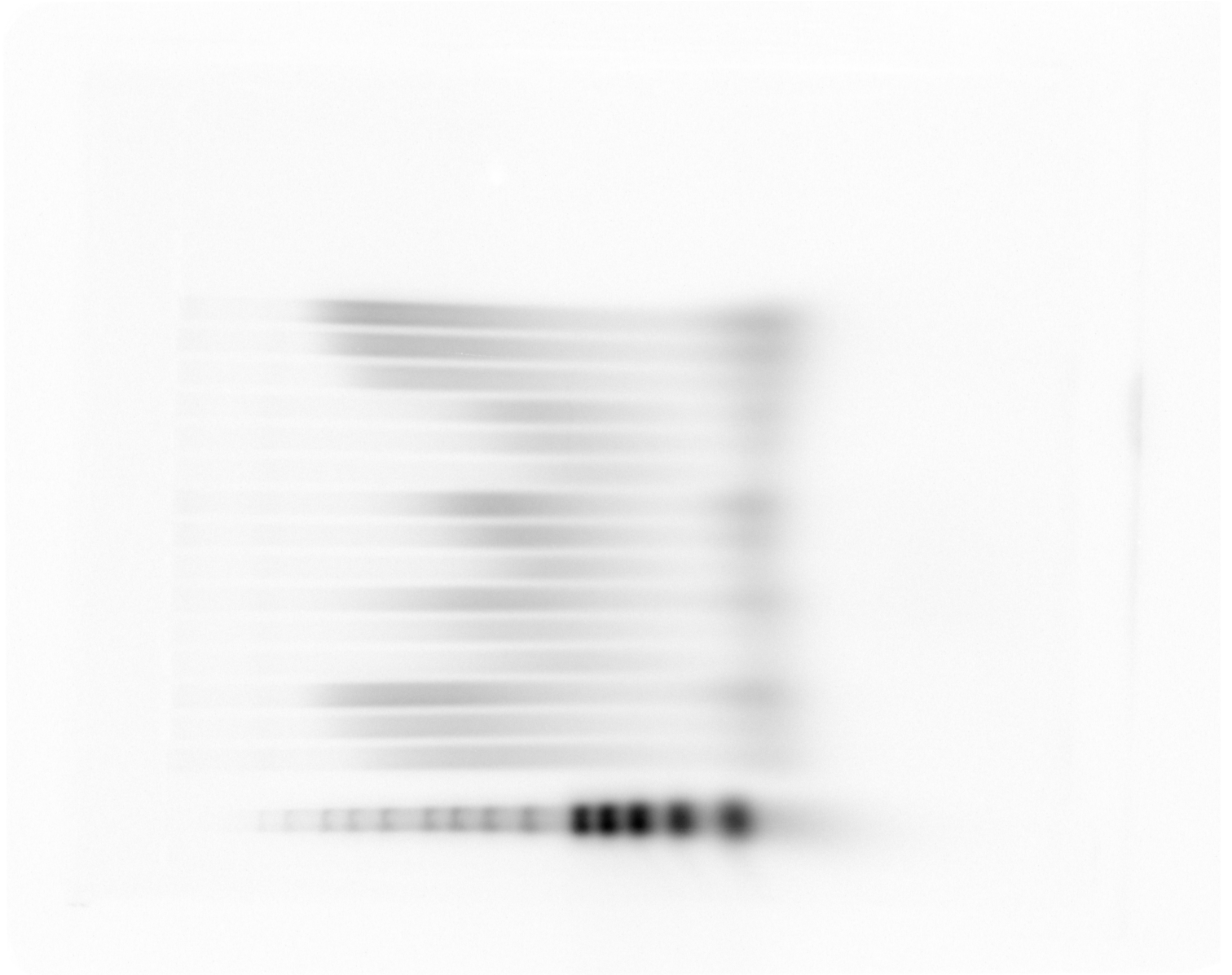

Supplement: Figure 3—source data 1. [file elife-69726-fig3-data1.pdf]

---

Figure 4 - source data 1.pdf  
5000 x 4000

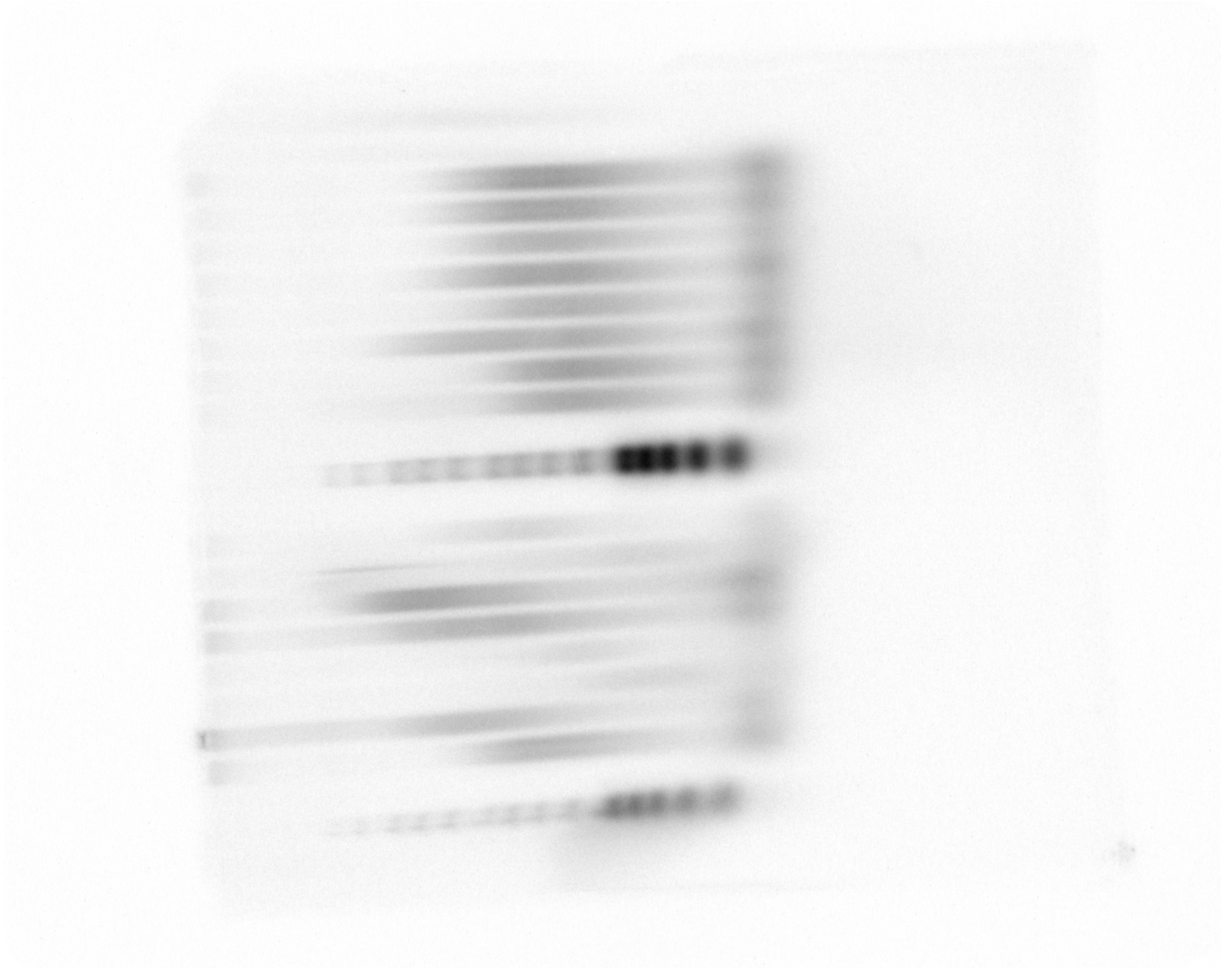

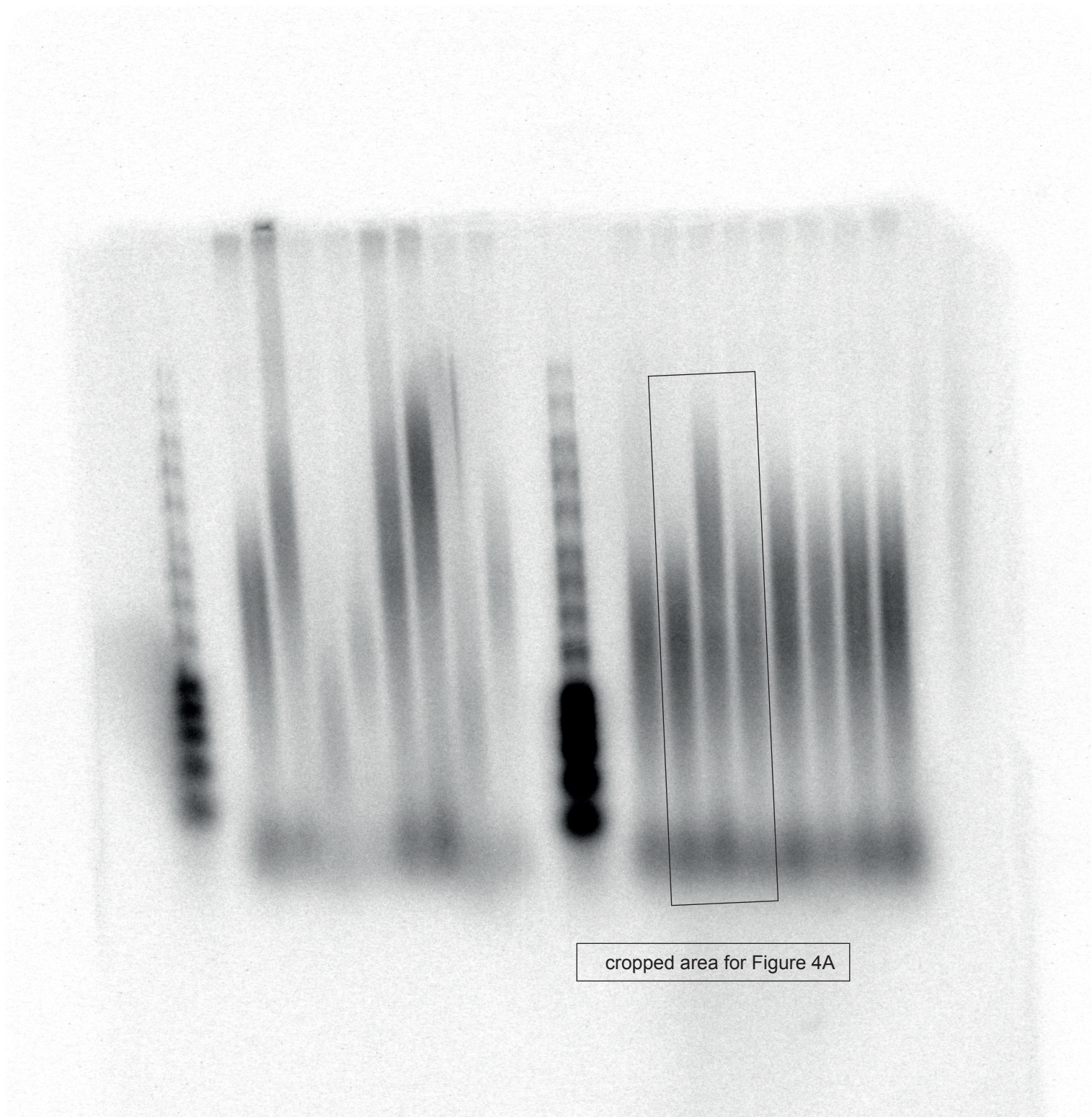

cropped area for Figure 4A

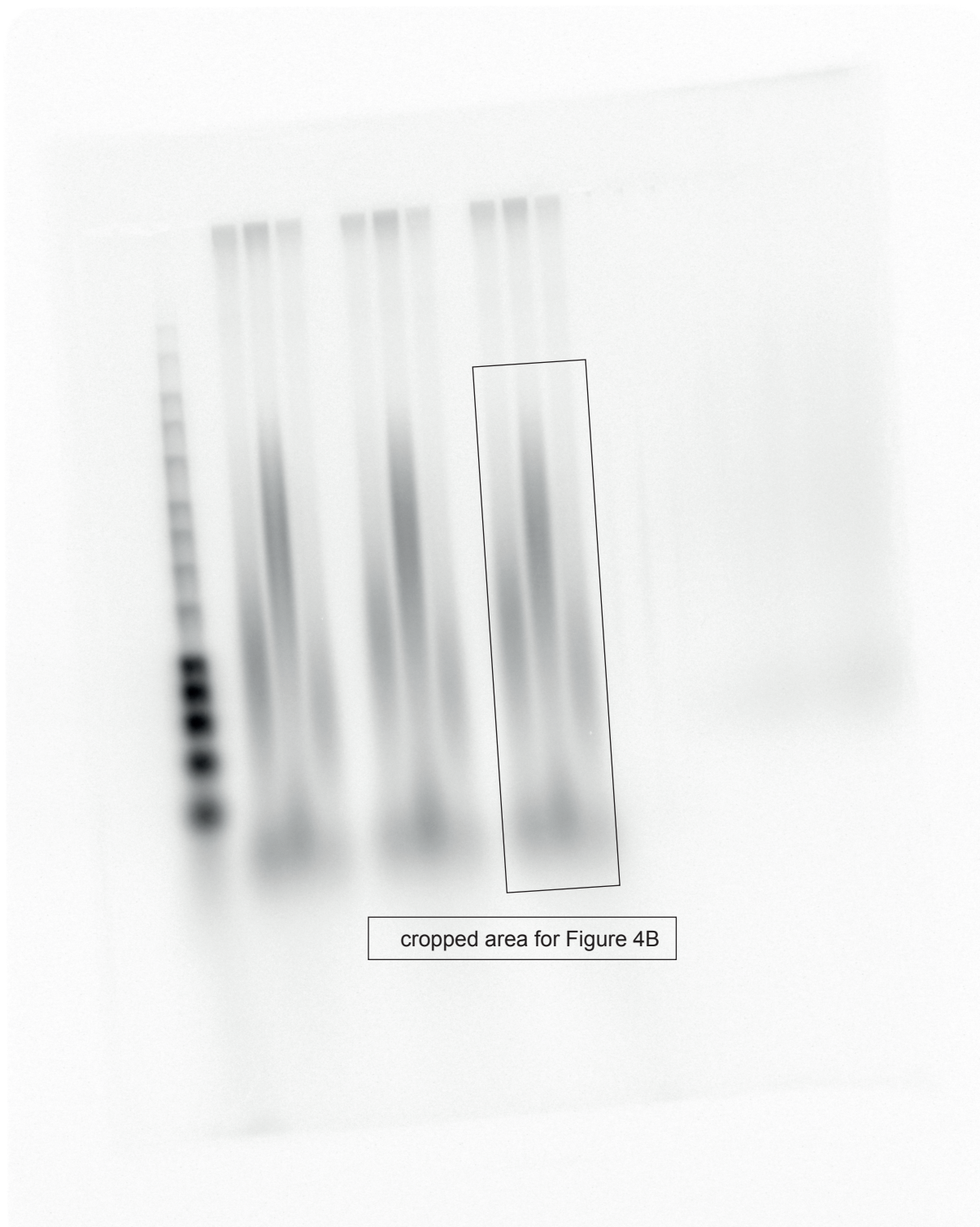

---

Figure 4 - source data 2.pdf  
5000 x 4000

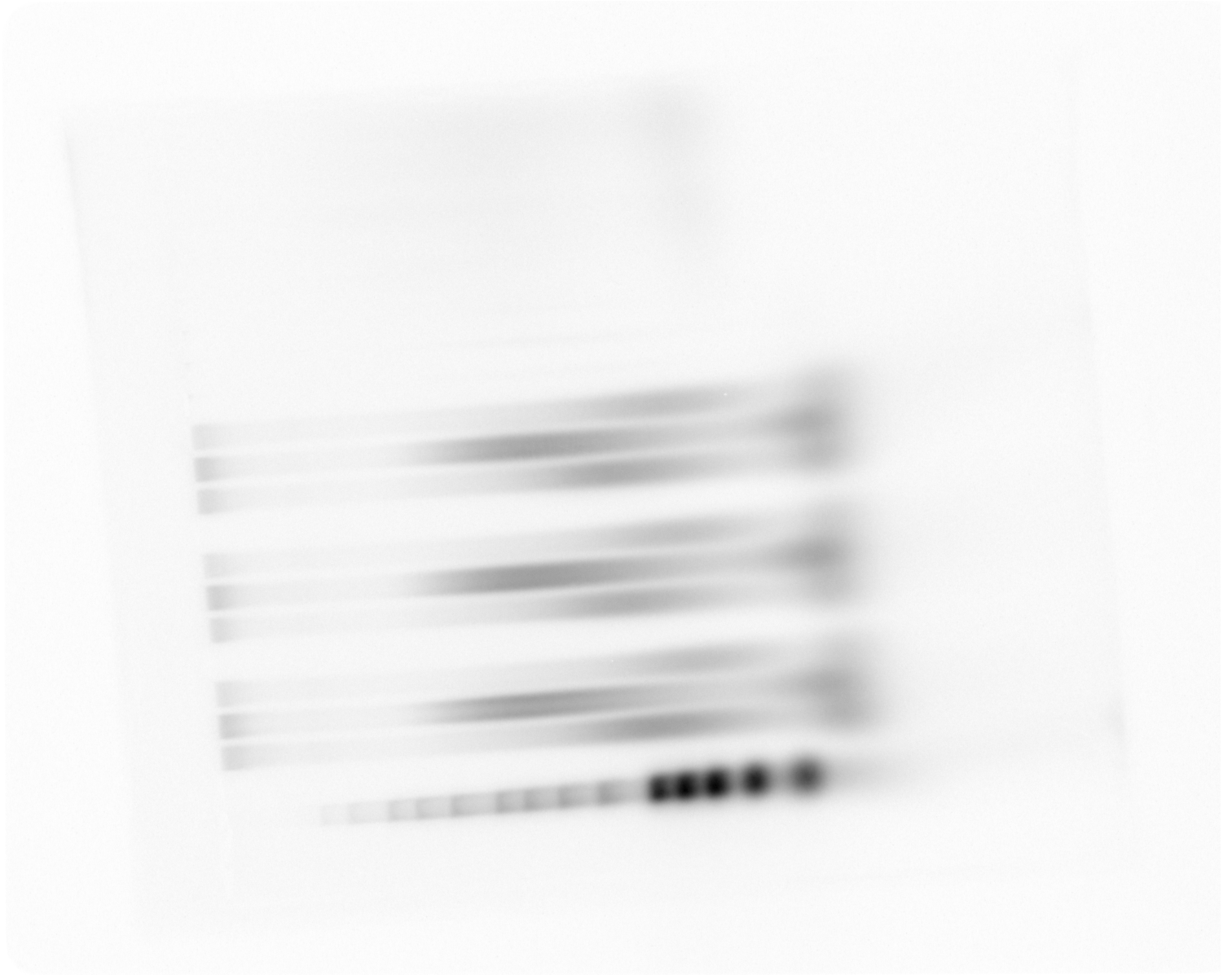

Supplement: Figure 4—source data 1. [file elife-69726-fig4-data1.pdf]

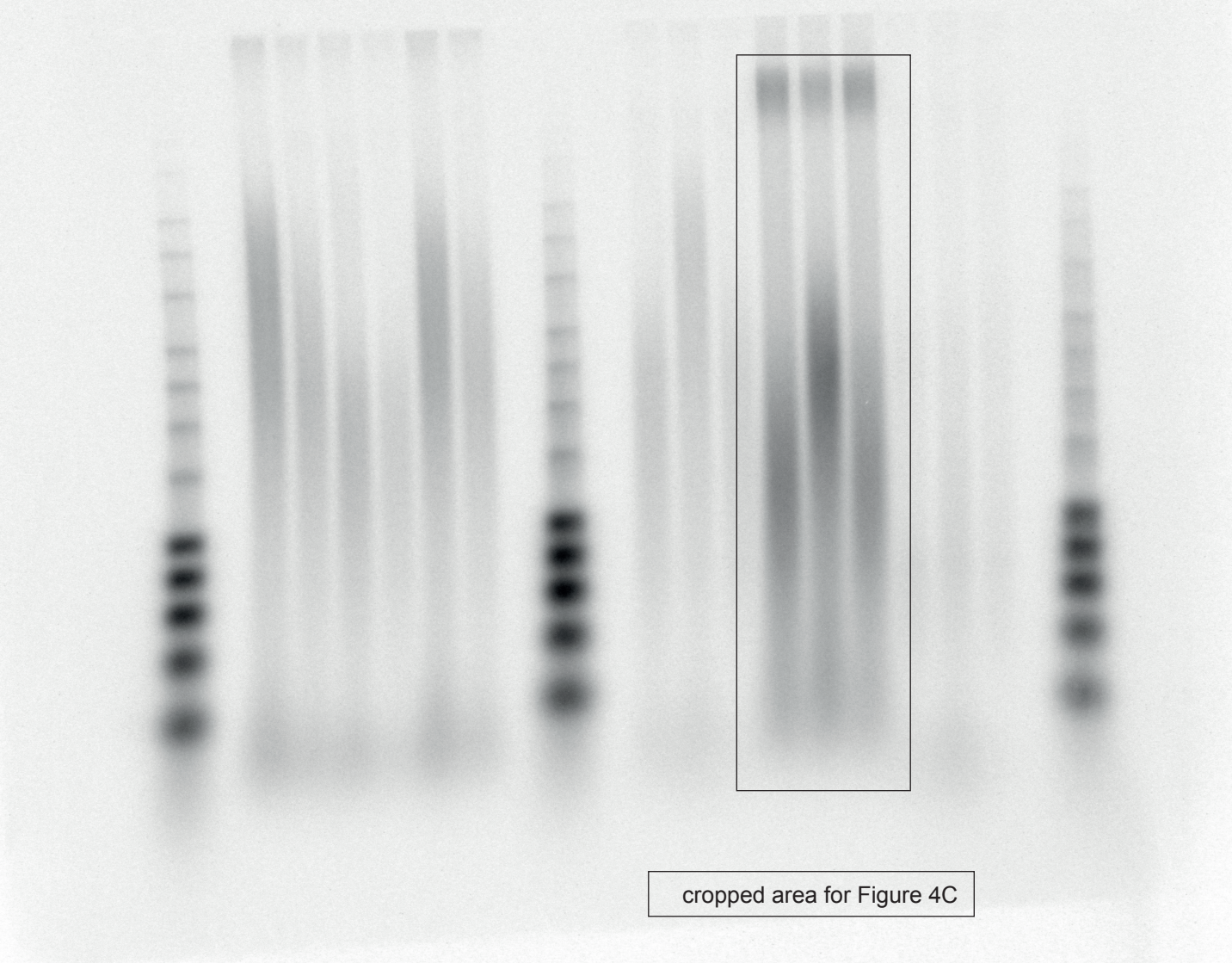

---

Figure 4 - source data 3.pdf  
5000 x 4000

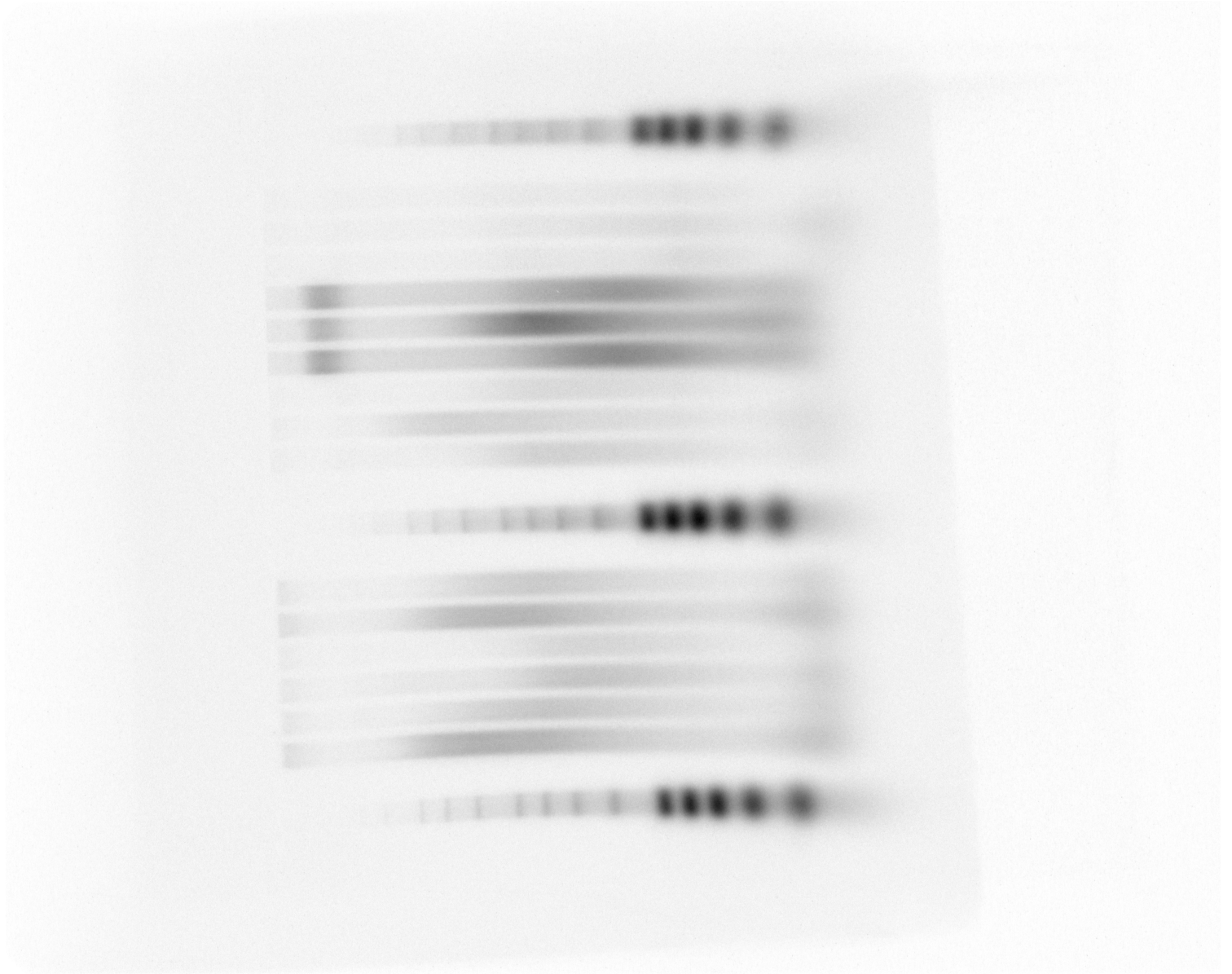

Supplement: Figure 4—source data 2. [file elife-69726-fig4-data2.pdf]

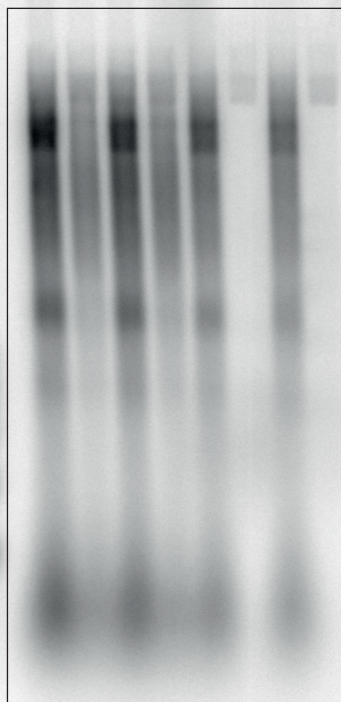

cropped area for Figure 4 - figure supplement 1

---

Figure 4 - figure supplement 1 - source data 1.pdf  
5000 x 4000

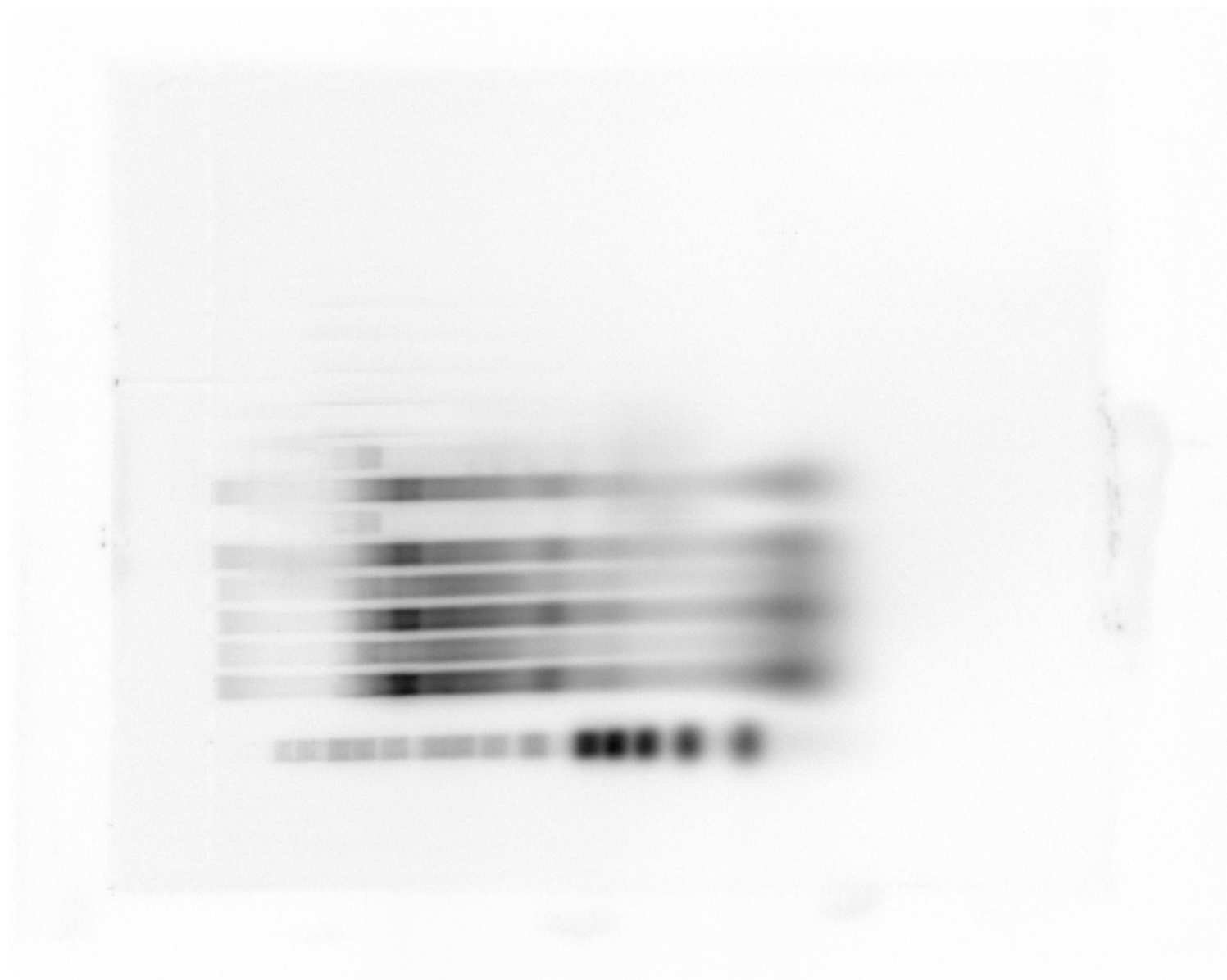

Supplement: Figure 4—figure supplement 1—source data 1. [file elife-69726-fig4-figsupp1-data1.pdf]
