# Supplementary material for: Rad53 checkpoint kinase regulation of DNA replication fork rate via Mrc1 phosphorylation
Source: eLife. 2021 Aug 13;10:e69726. doi: 10.7554/eLife.69726 (PMC8387023; doi:10.7554/eLife.69726)

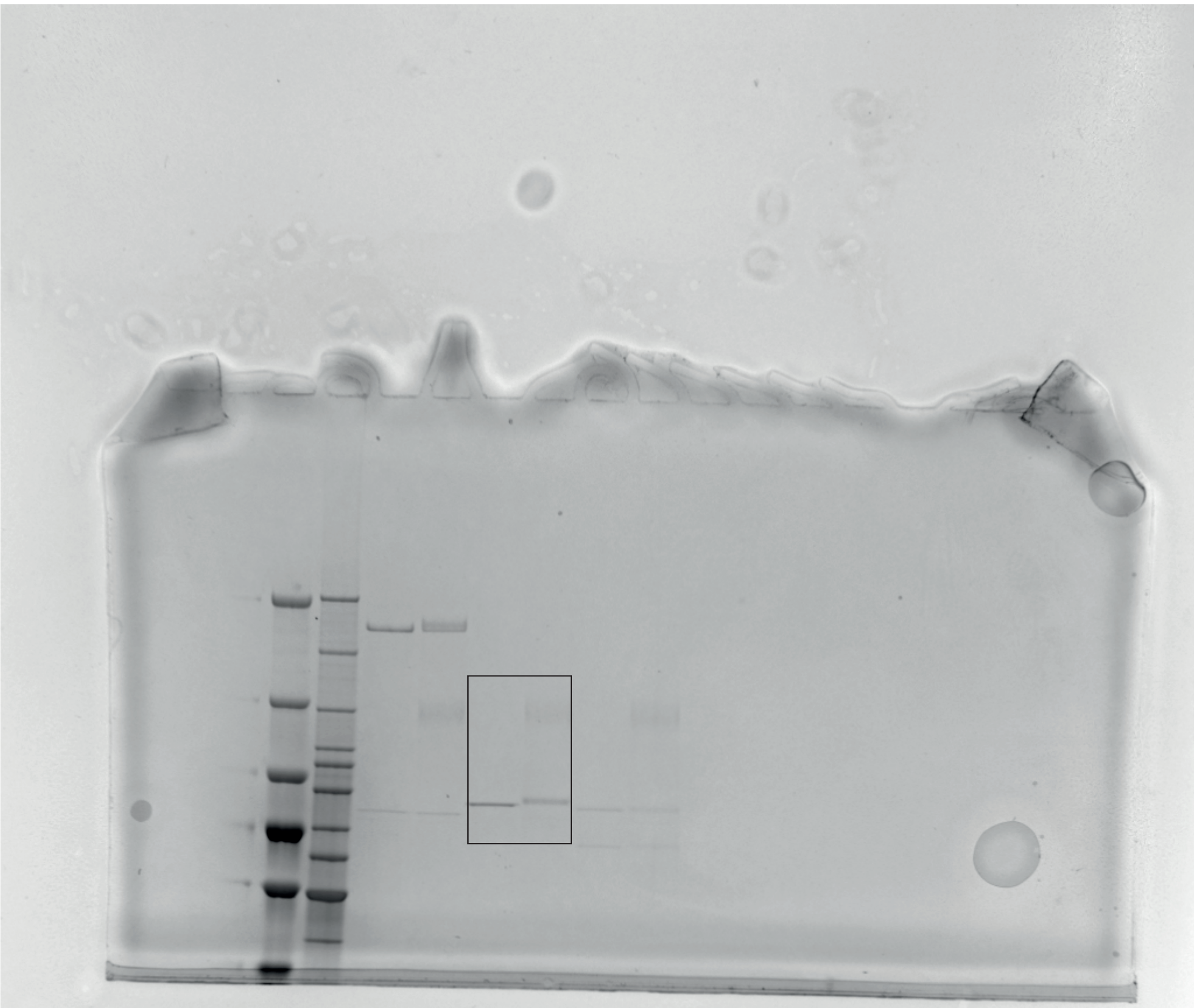

cropped area for Figure 3C

Figure 3 - source data 3.pdf
2048 x 2816

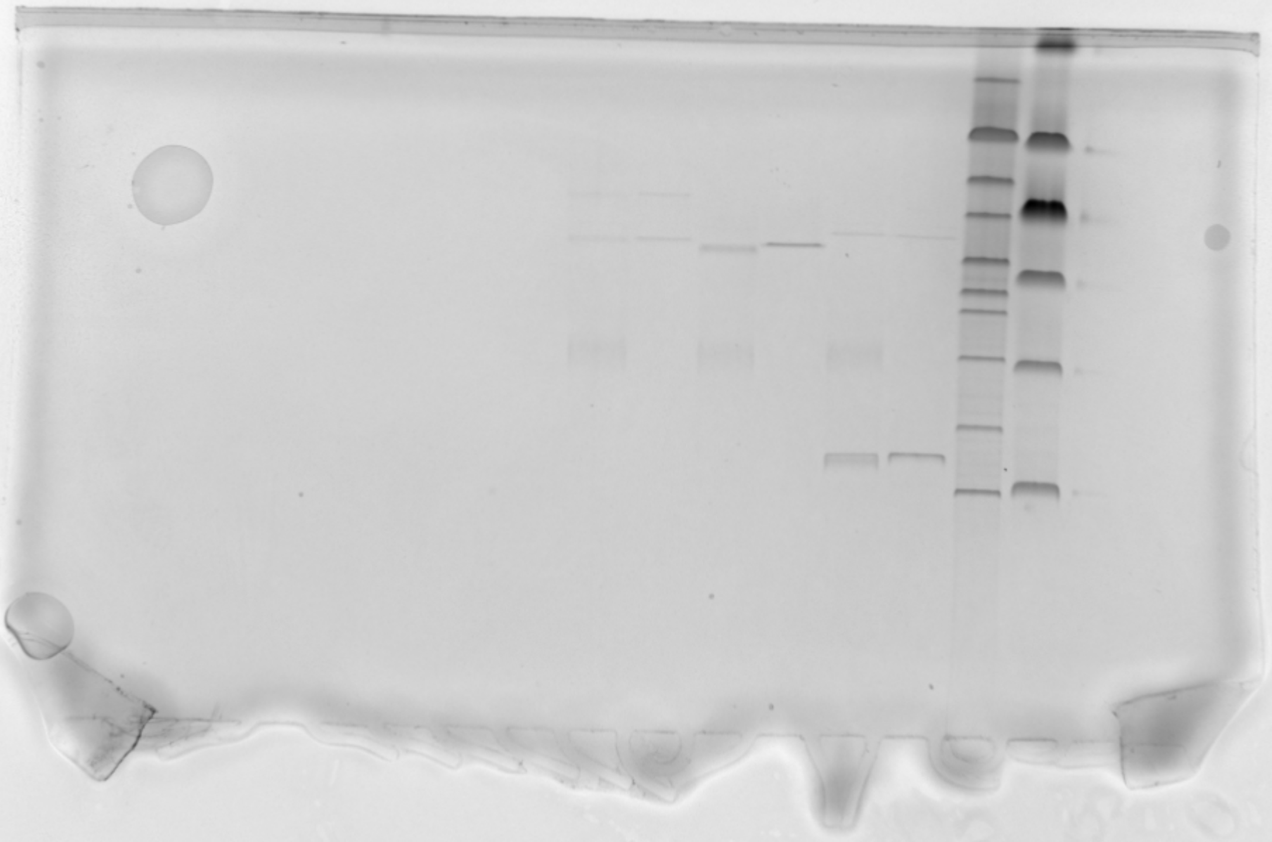

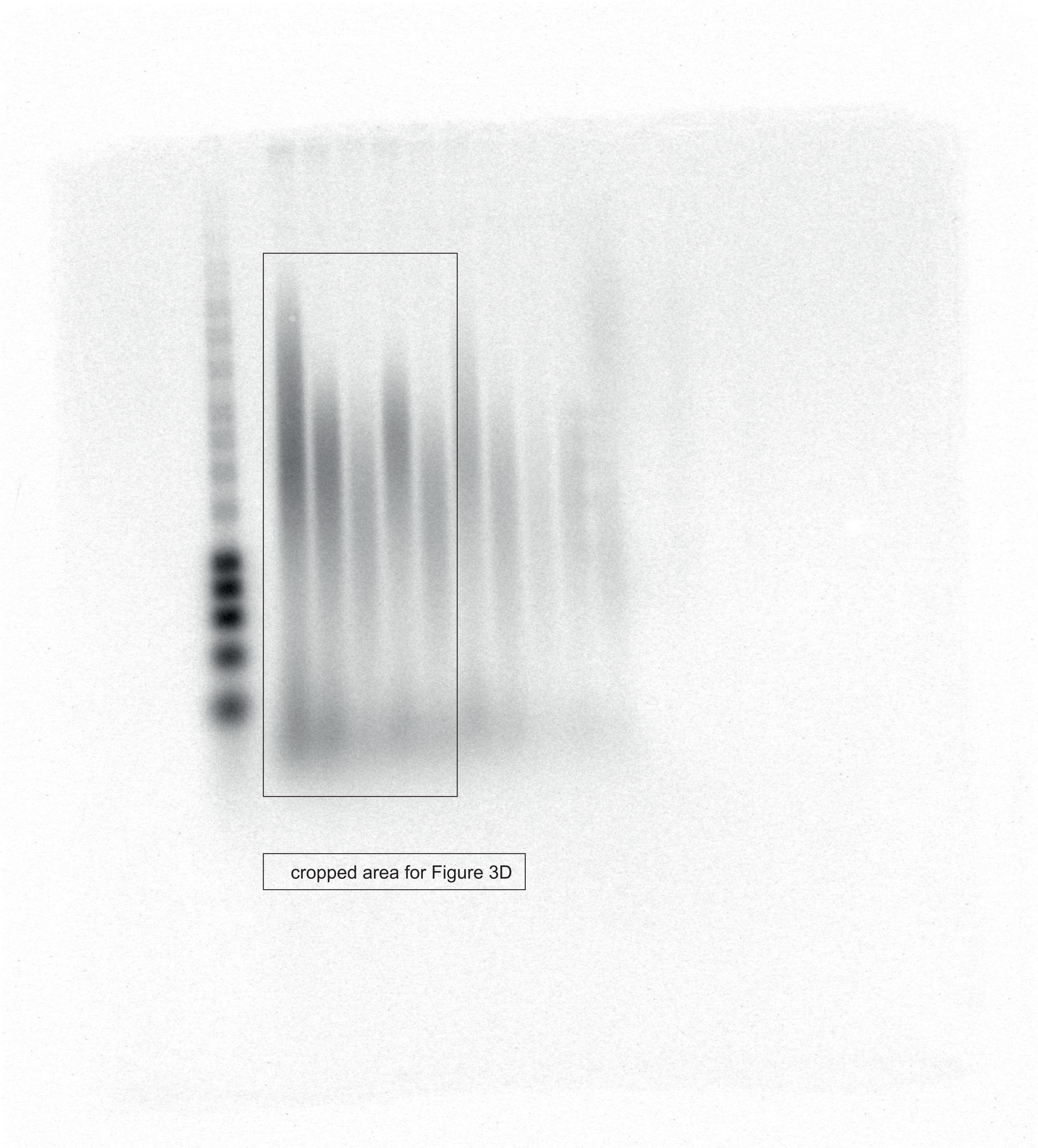

cropped area for Figure 3D

Figure 3 - source data 4.pdf

5000 x 4000

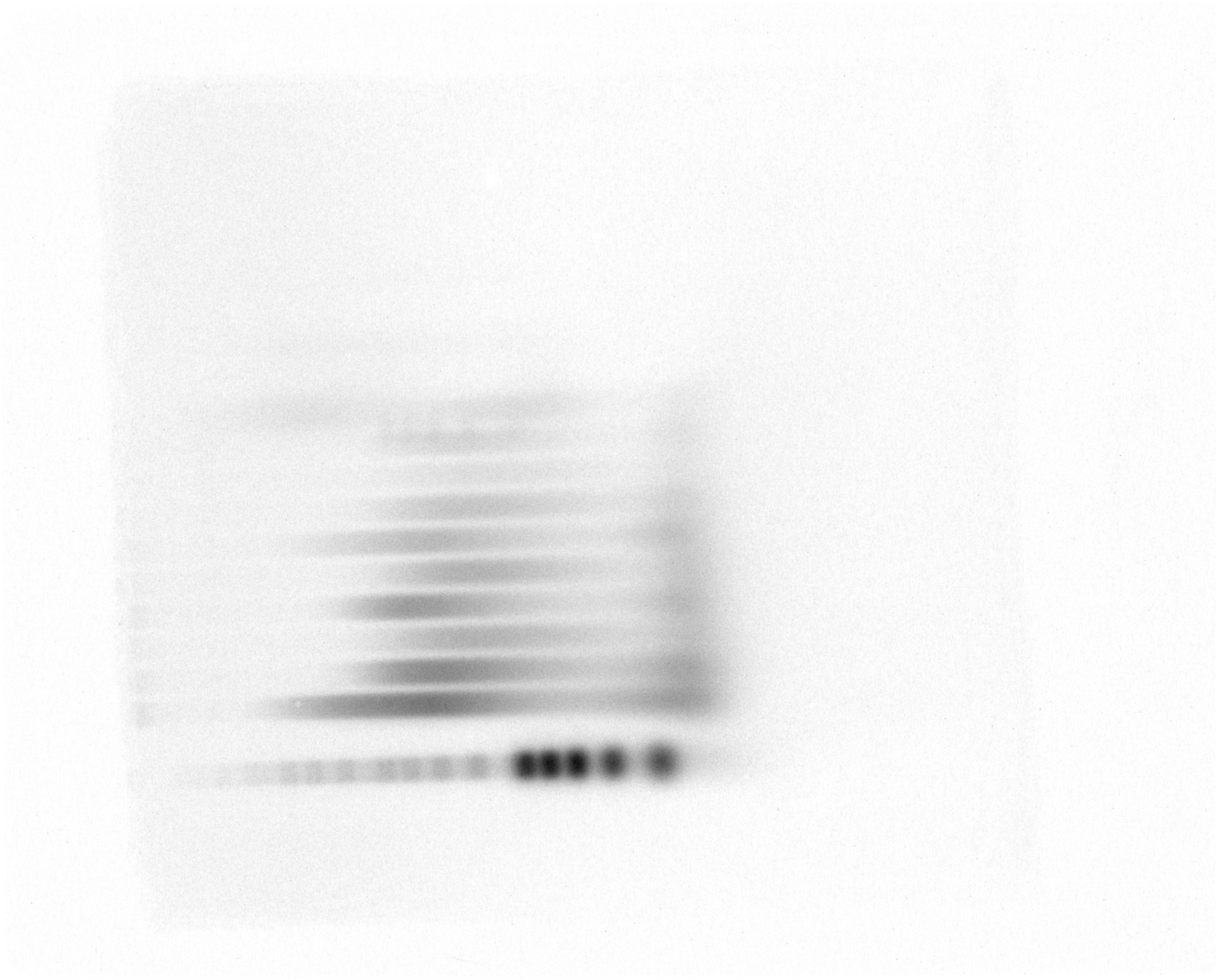

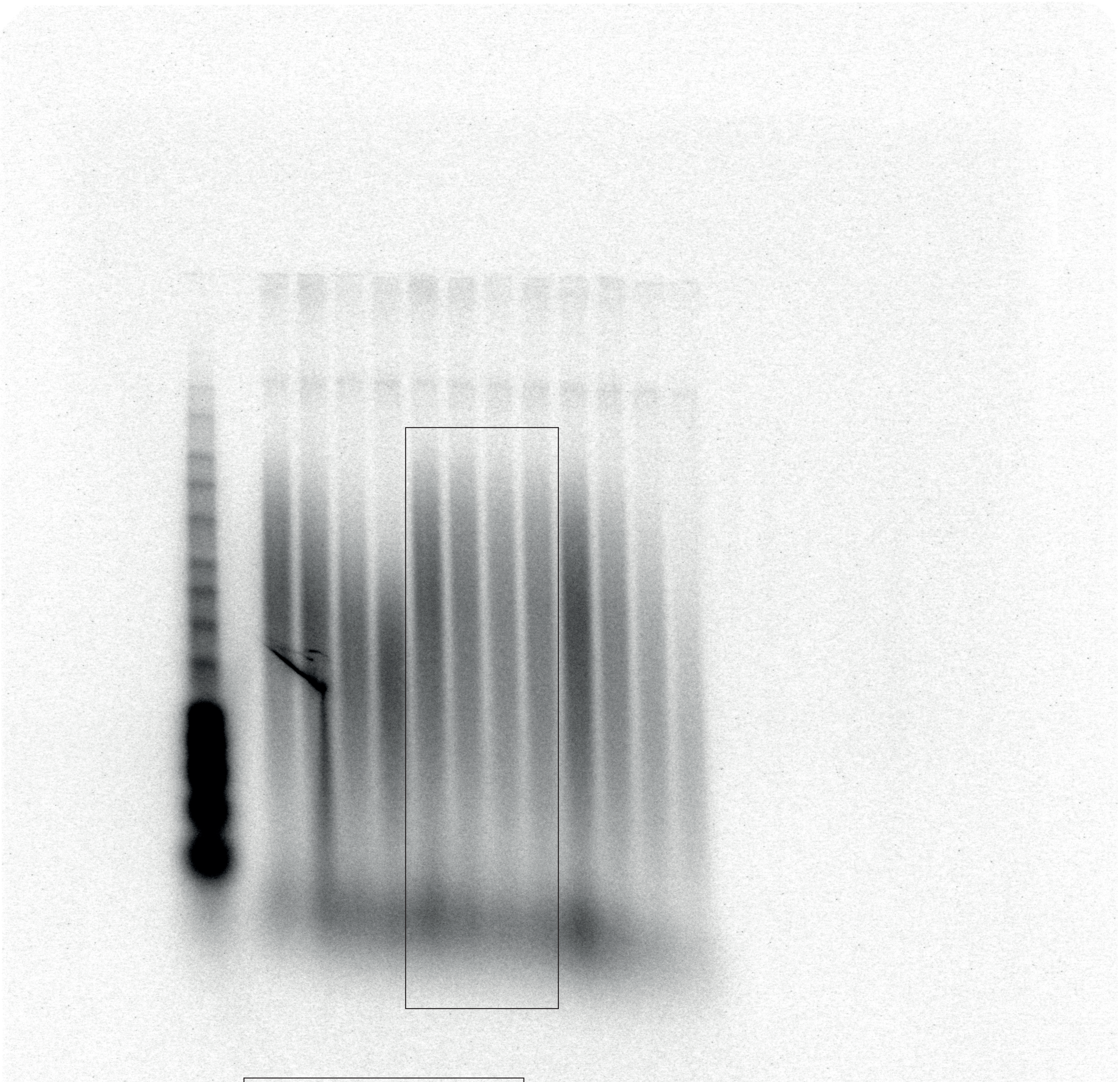

cropped area for Figure 3E

Figure 3 - source data 5.pdf
5000 x 4000

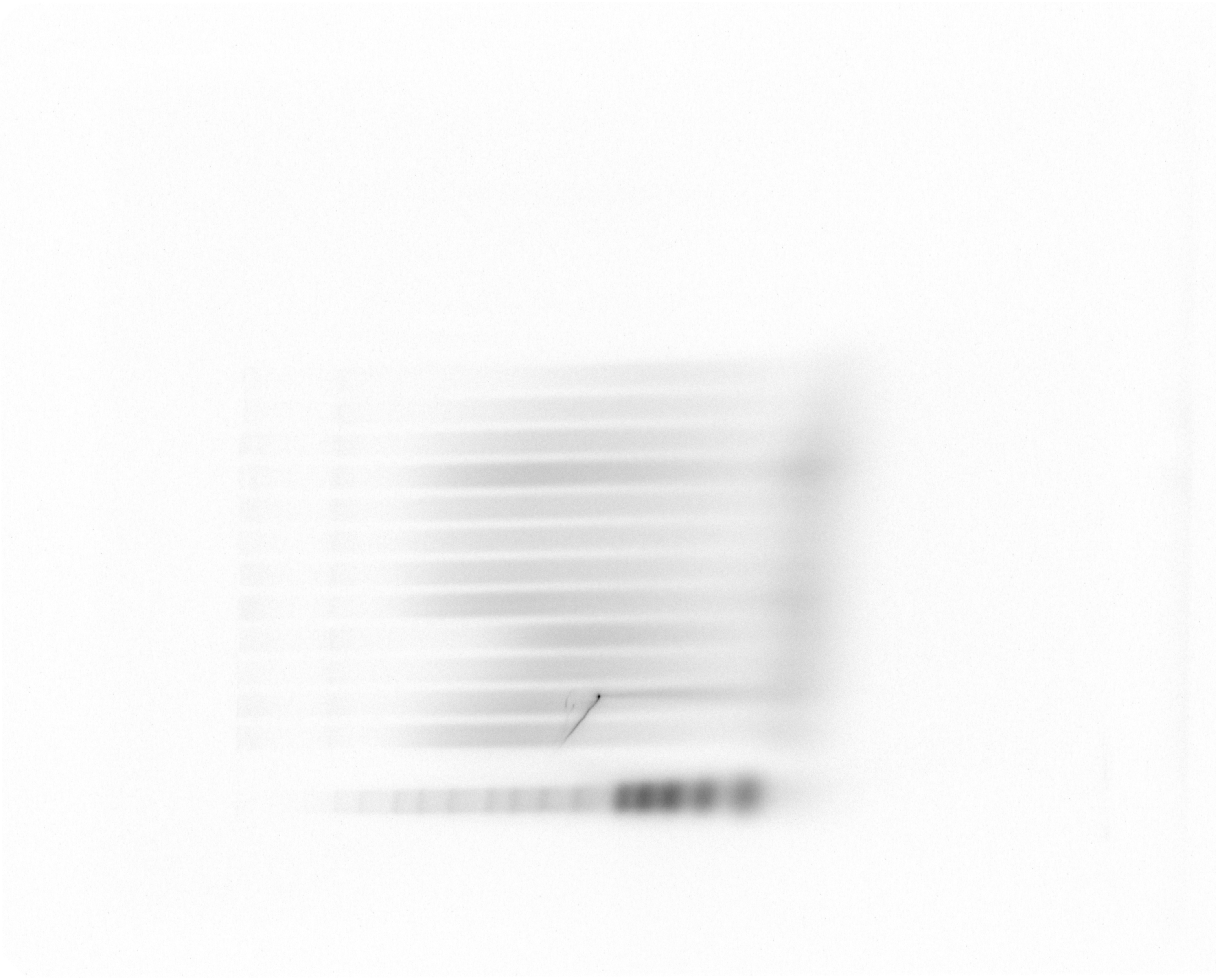

Supplement: Figure 3—source data 2. [file elife-69726-fig3-data2.pdf]
