# Supplementary material for: Rad53 checkpoint kinase regulation of DNA replication fork rate via Mrc1 phosphorylation
Source: eLife. 2021 Aug 13;10:e69726. doi: 10.7554/eLife.69726 (PMC8387023; doi:10.7554/eLife.69726)

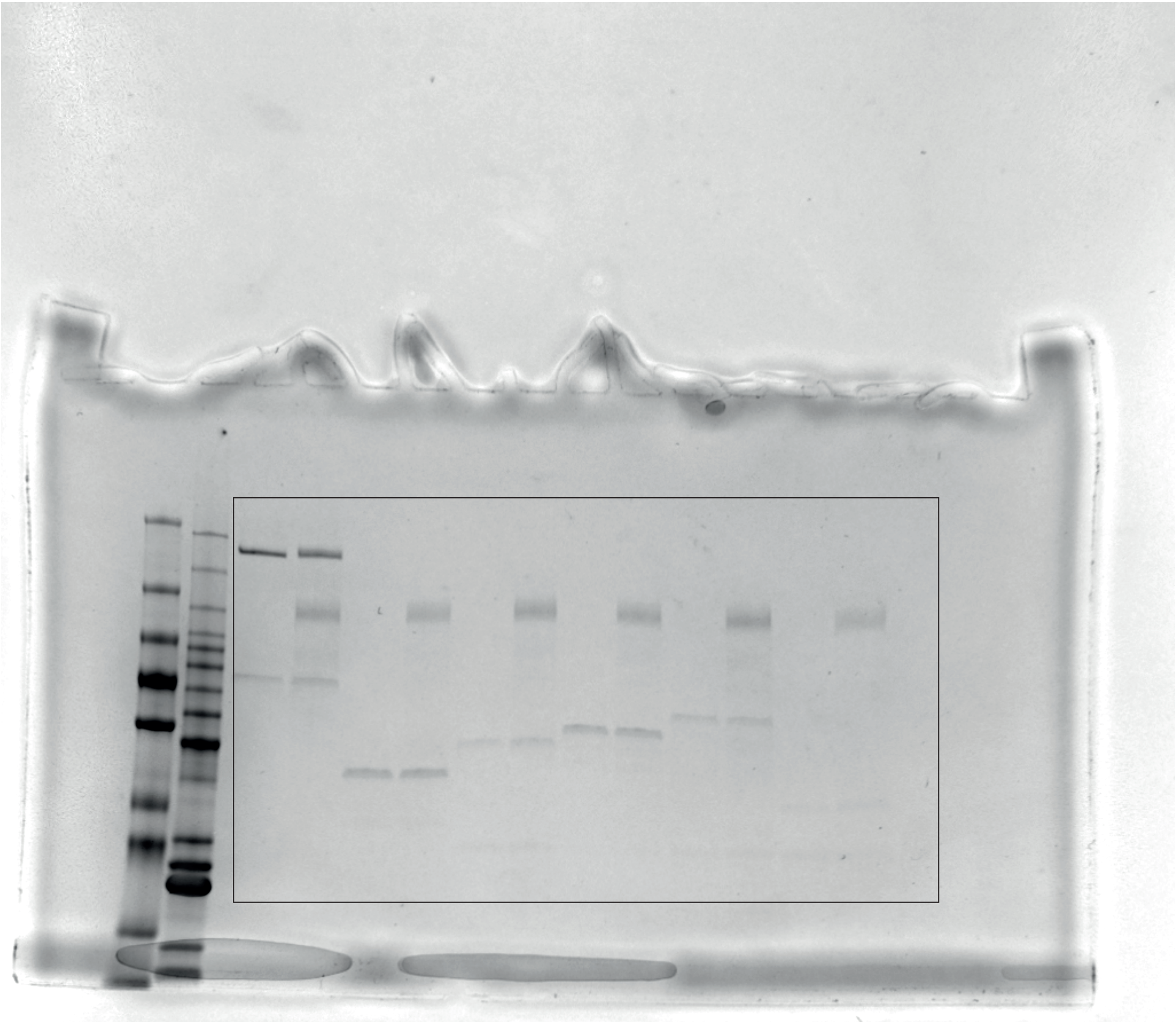

cropped area for Figure 5D top

Figure 5 - source data 4.pdf

2048 x 2816

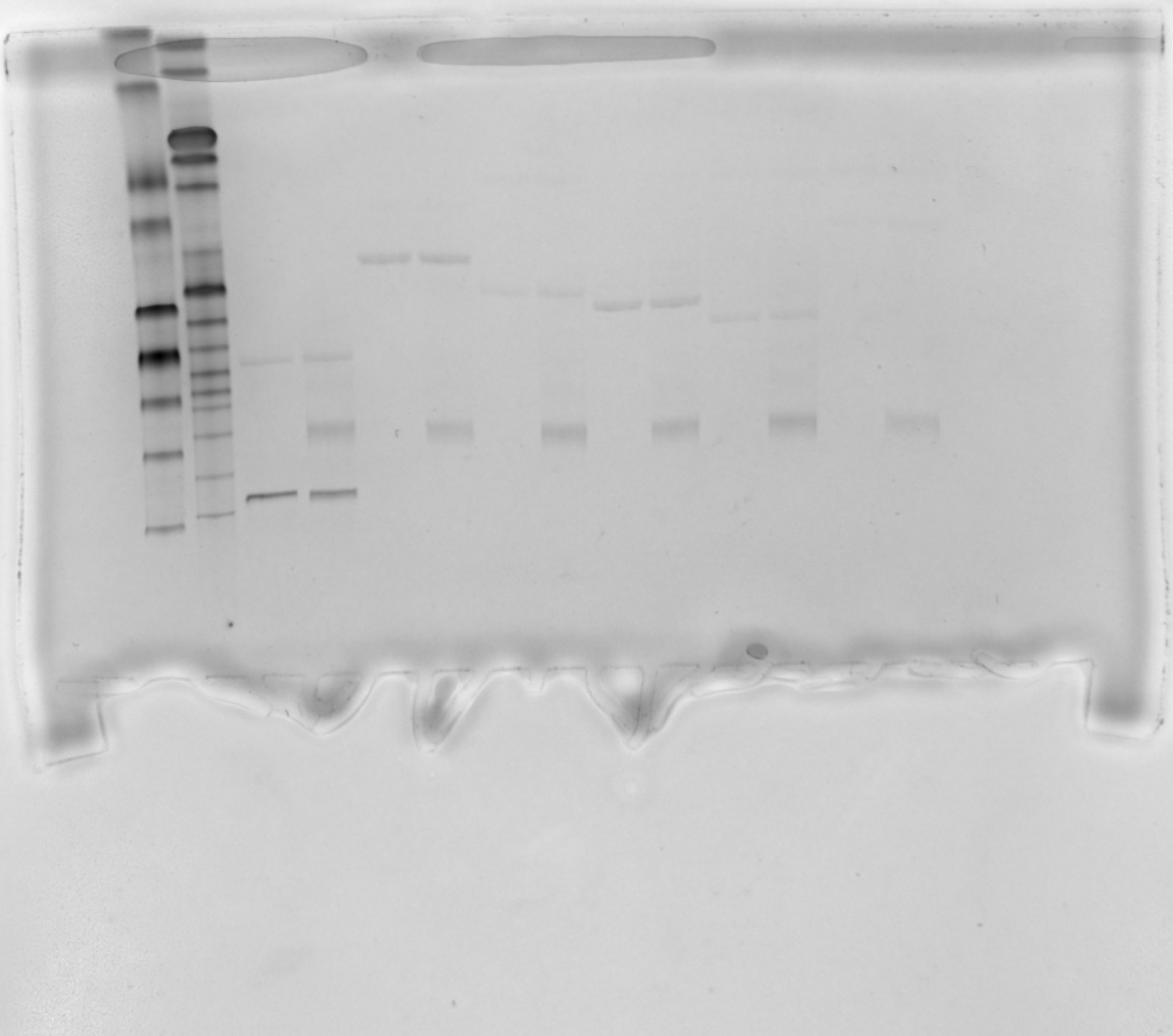

Figure 5 - source data 5.pdf

5000 x 4000

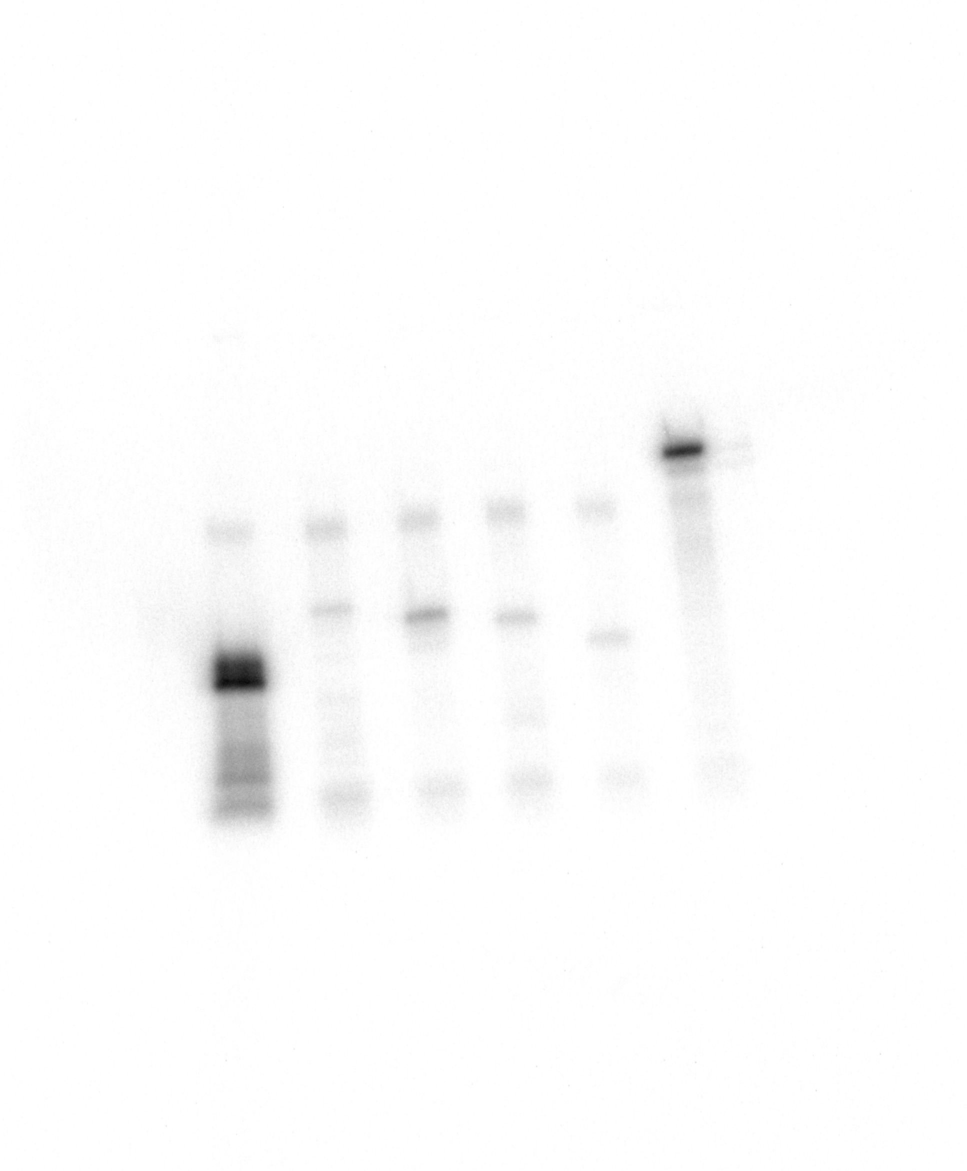

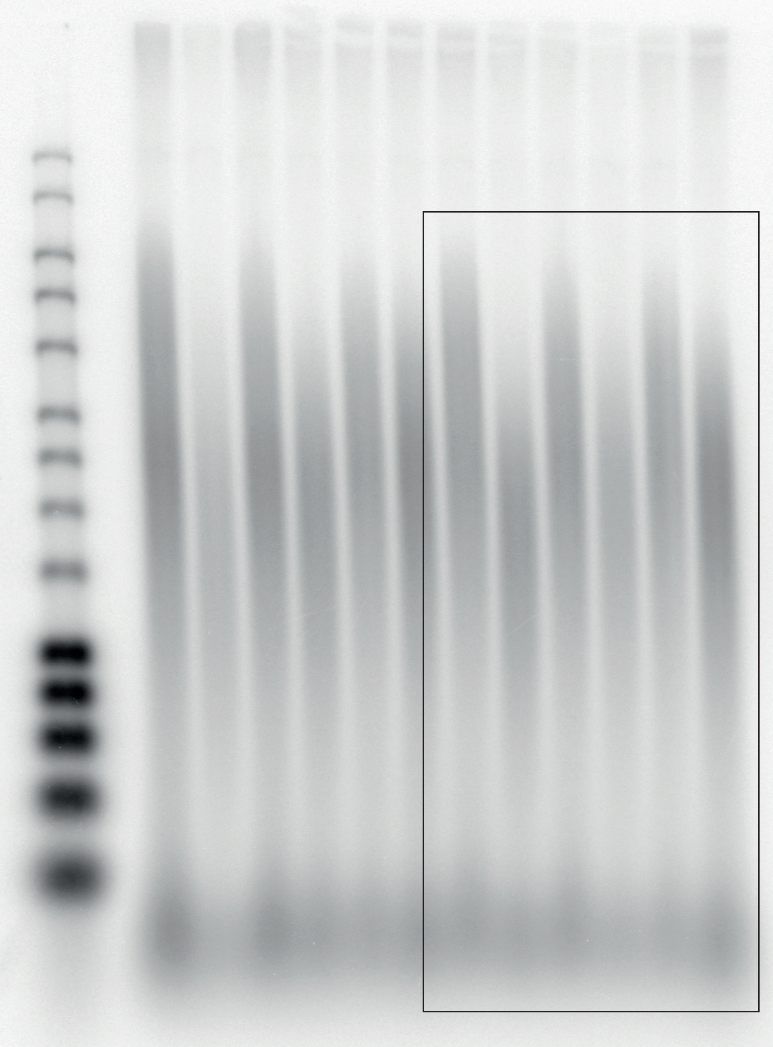

cropped area for Figure 5E

Figure 5 - source data 6.pdf

5000 x 4000

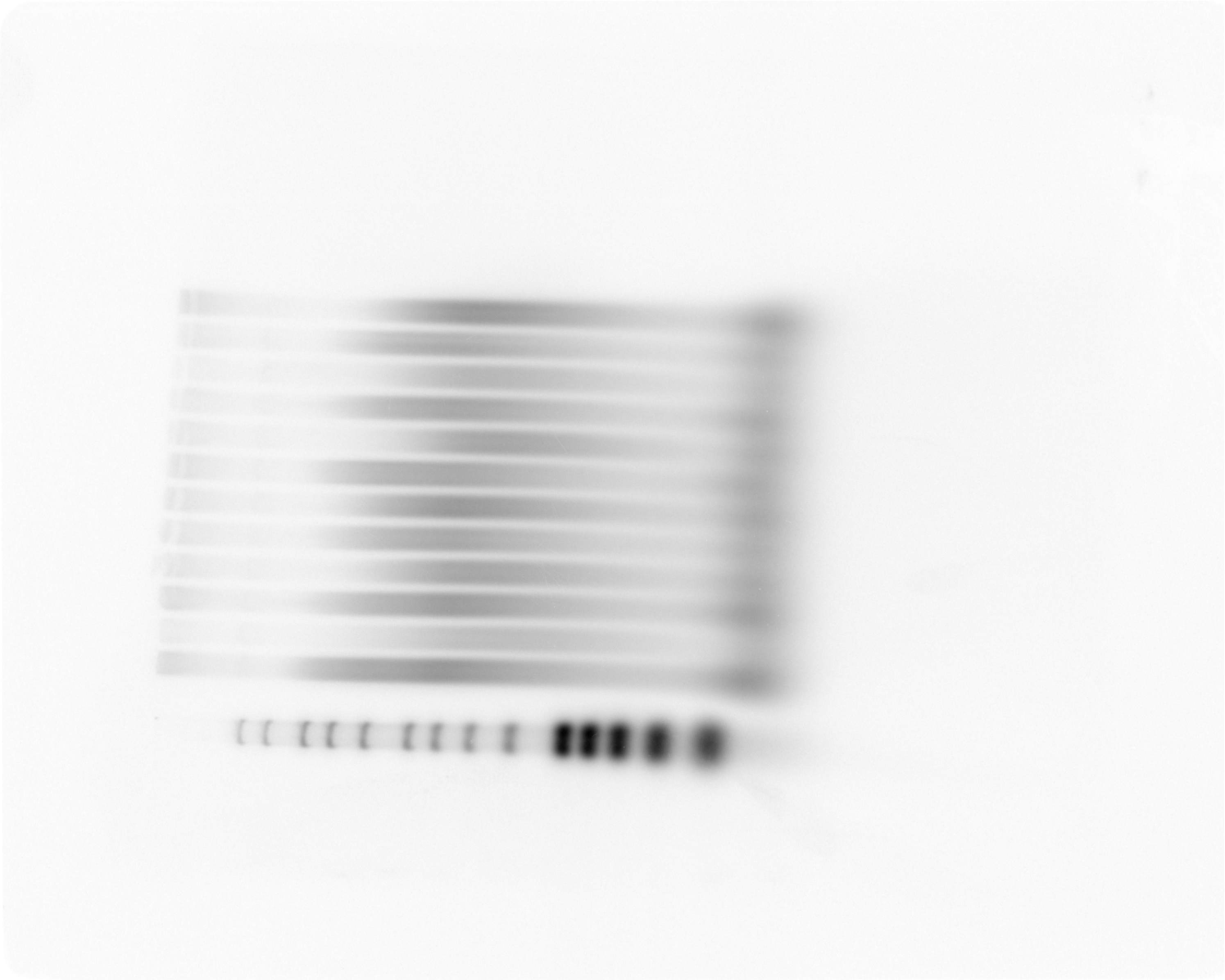

Supplement: Figure 5—source data 2. [file elife-69726-fig5-data2.pdf]
