# Supplementary material for: Rad53 checkpoint kinase regulation of DNA replication fork rate via Mrc1 phosphorylation
Source: eLife. 2021 Aug 13;10:e69726. doi: 10.7554/eLife.69726 (PMC8387023; doi:10.7554/eLife.69726)

Figure 5 - figure supplement 1 - source data 2.pdf
5000 x 4000

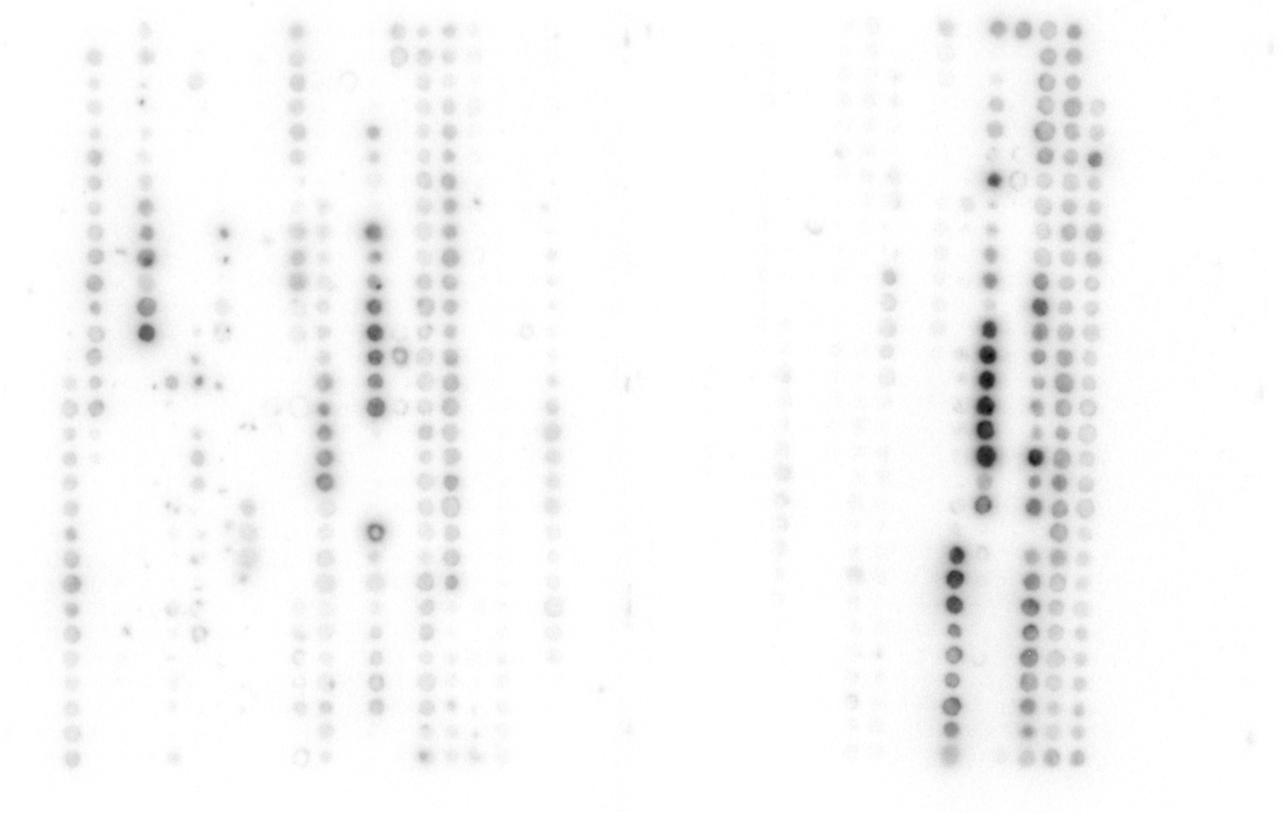

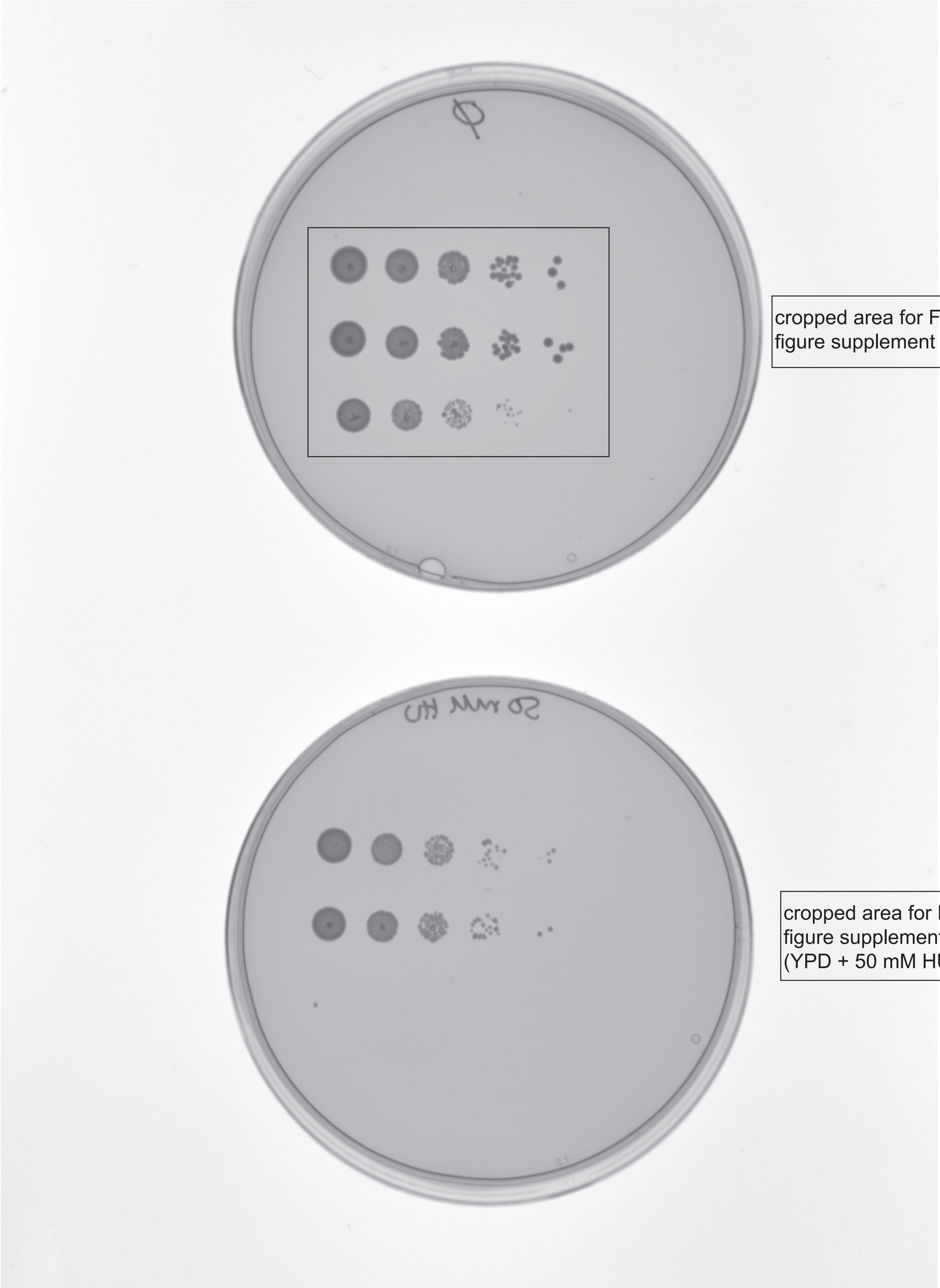

cropped area for Figure 5 - figure supplement 1C (YPD)

cropped area for Figure 5 - figure supplement 1C (YPD + 50 mM HU)

Figure 5 - figure supplement 1 - source data 4.pdf
2048 x 2816

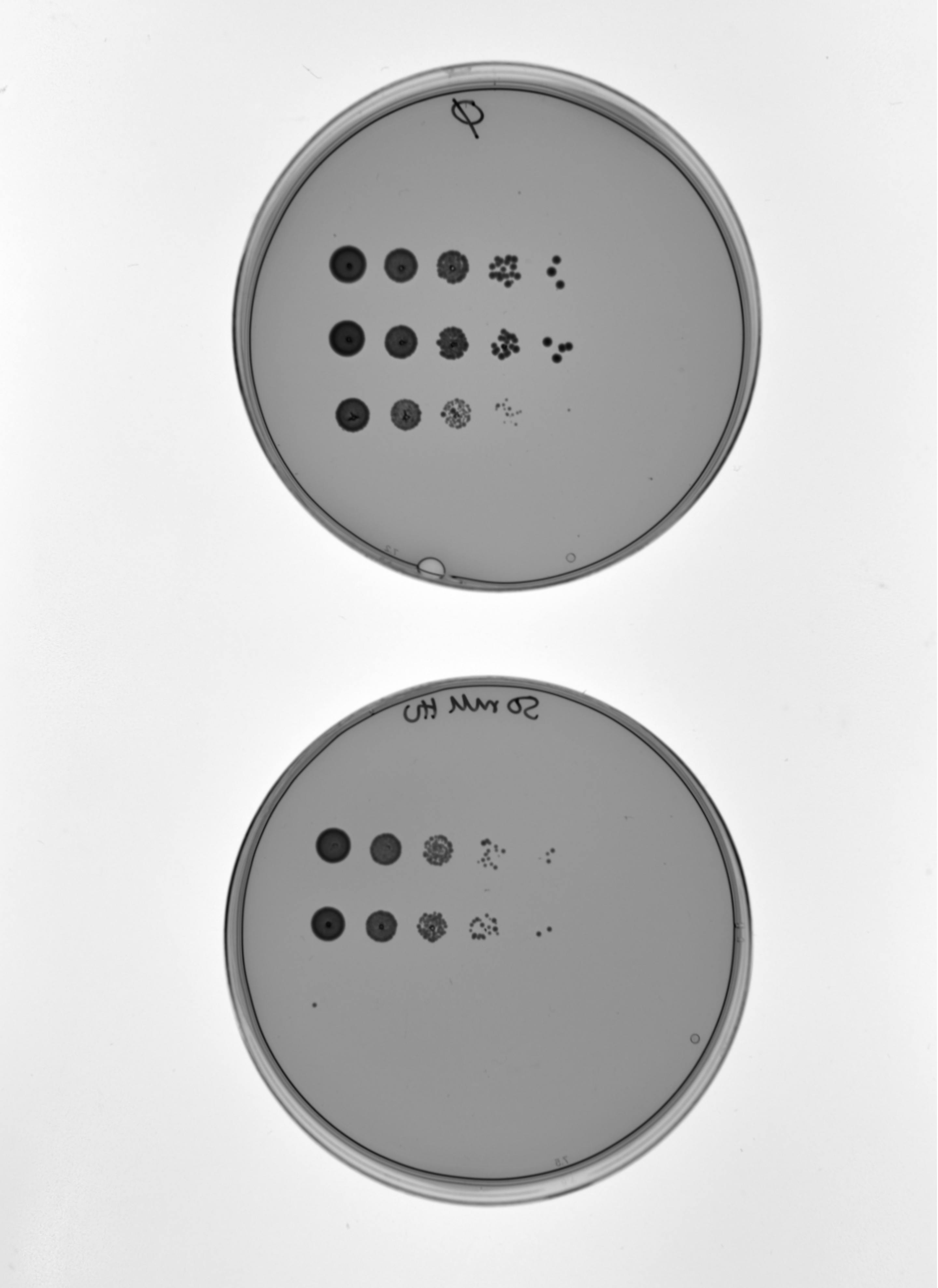
Ø
50 mM HU

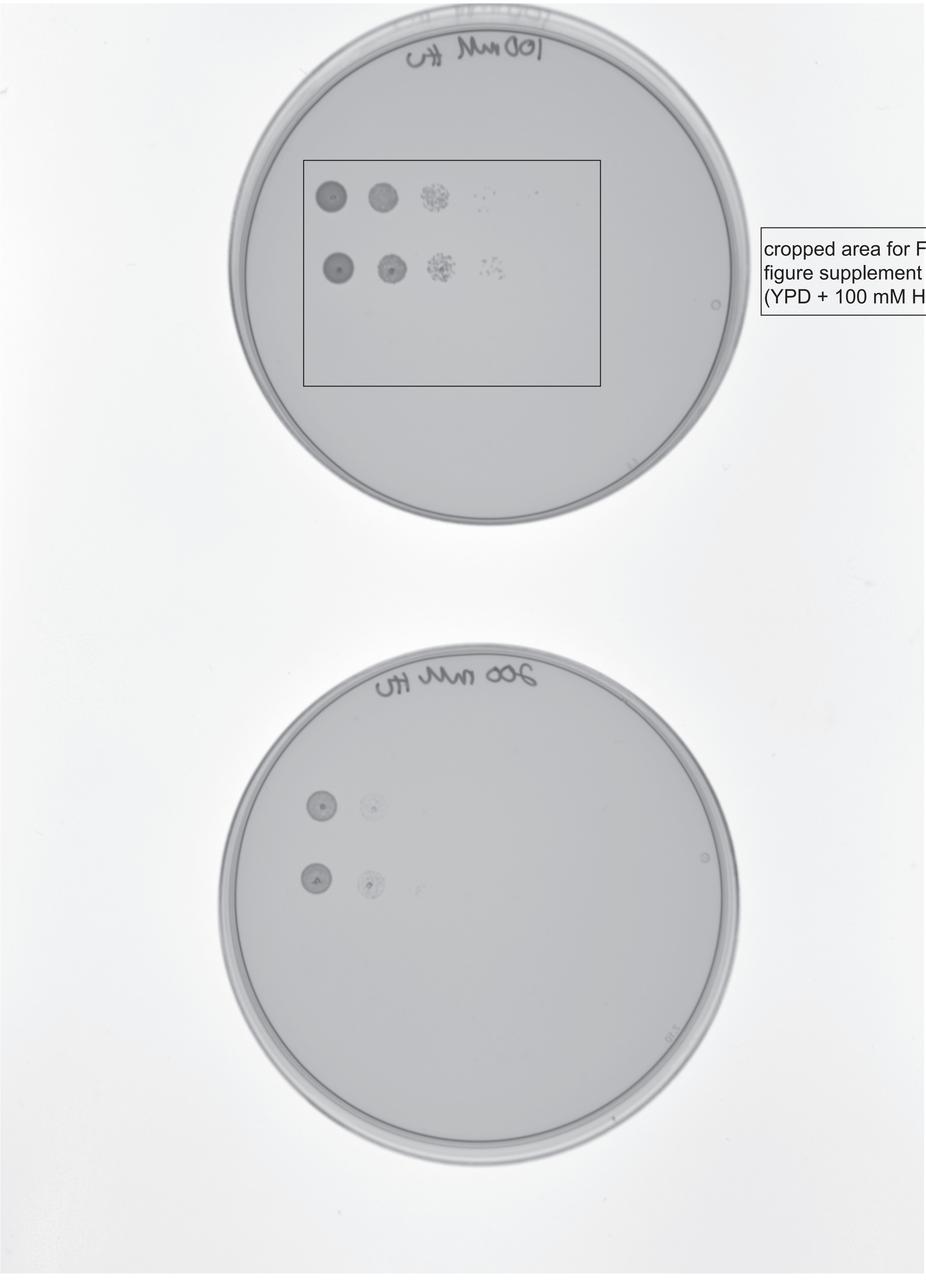

cropped area for Figure 5 -
figure supplement 1C
(YPD + 100 mM HU)

Figure 5 - figure supplement 1 - source data 5.pdf

2048 x 2816

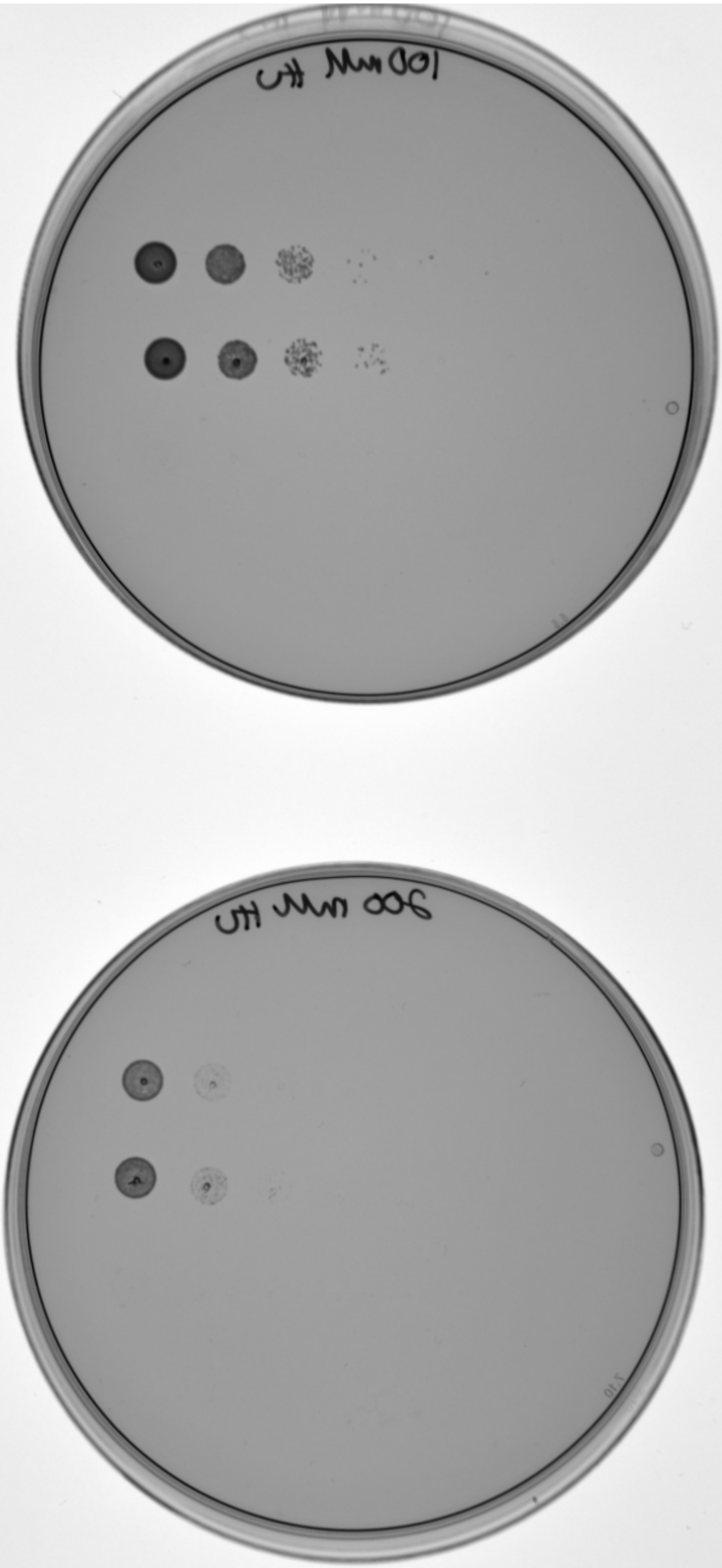
100 mM #5
200 mM #5

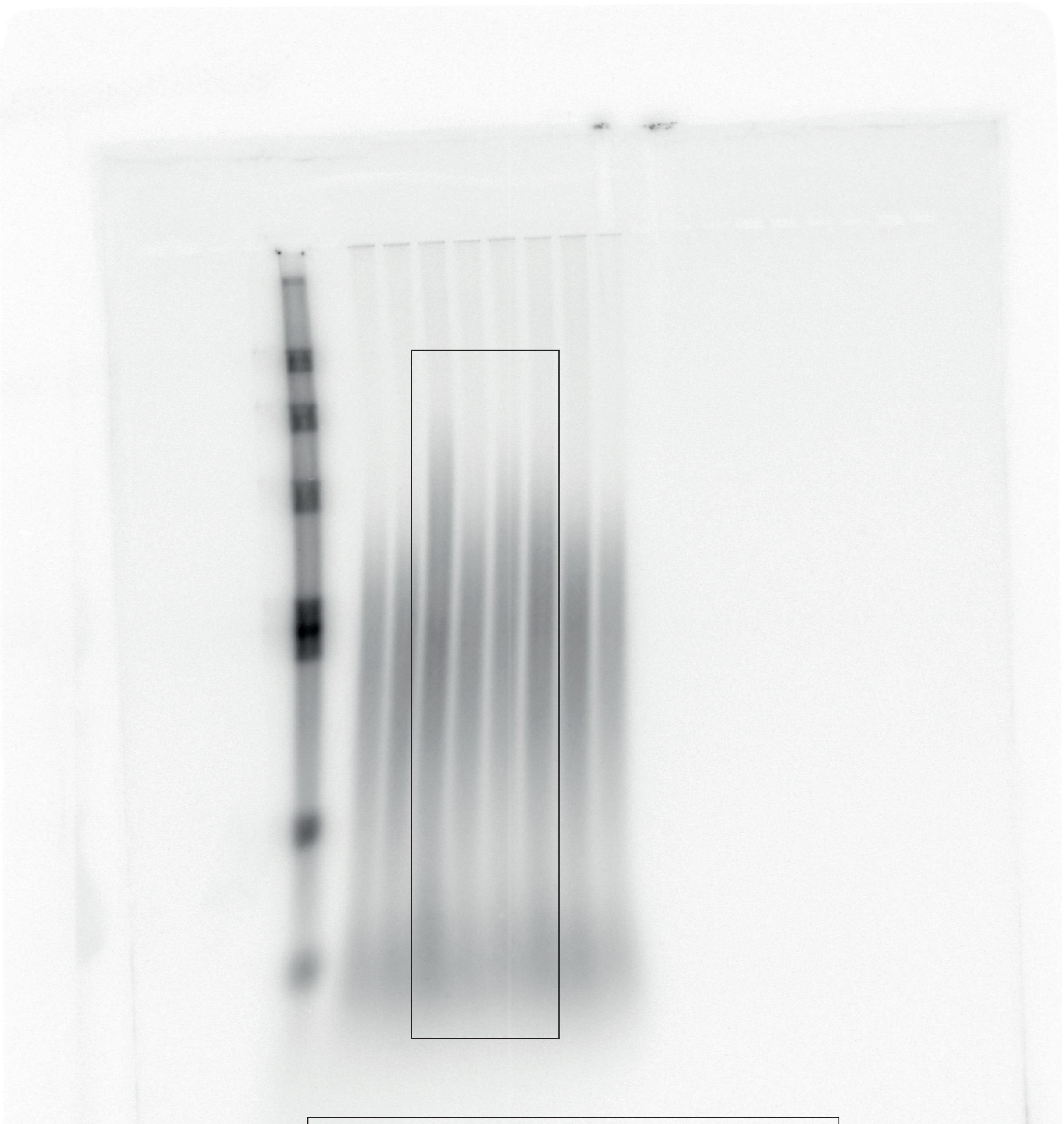

cropped area for Figure 5 - figure supplement 1D

Figure 5 - figure supplement 1 - source data 6.pdf

5000 x 4000

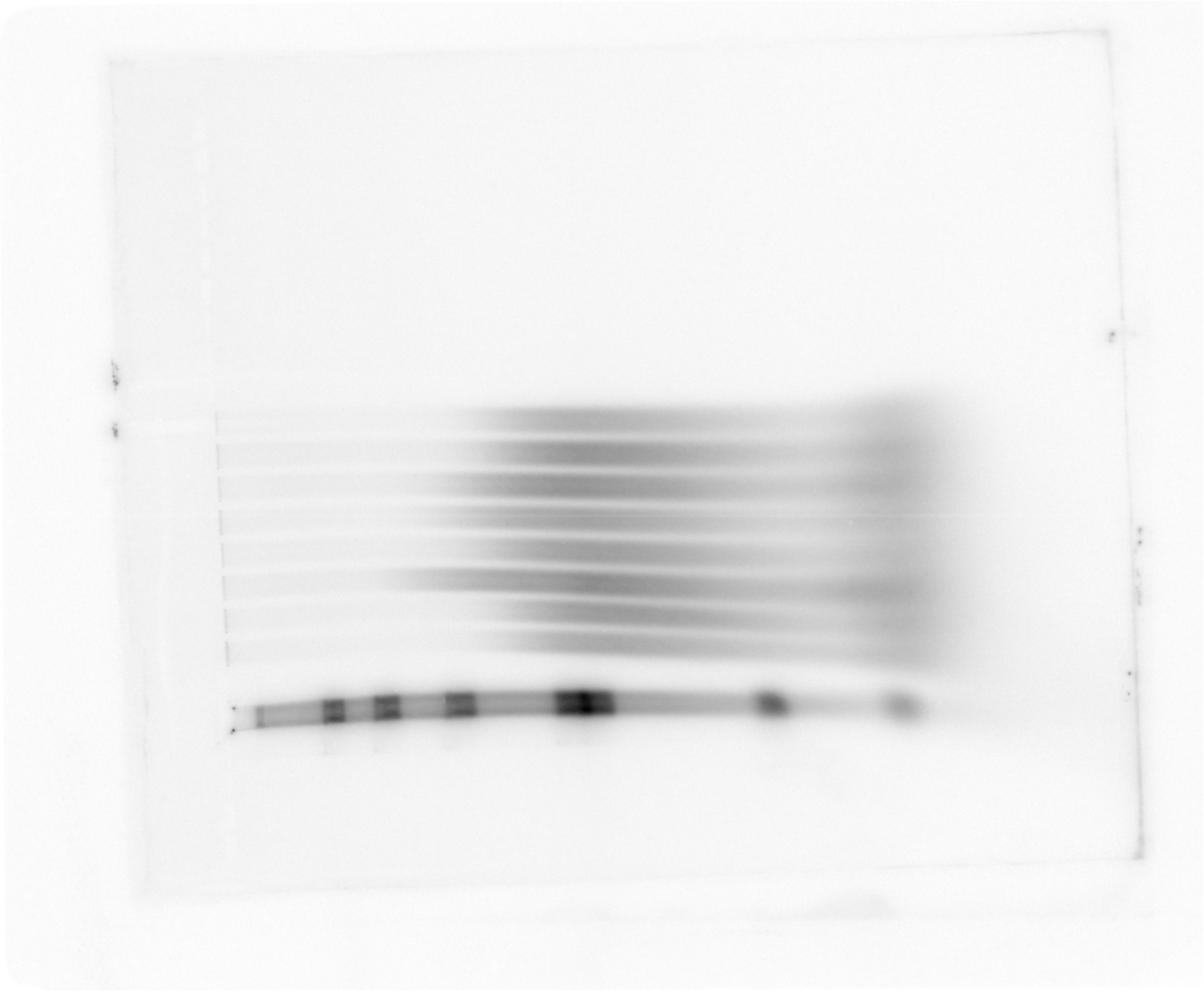

Supplement: Figure 5—figure supplement 1—source data 1. [file elife-69726-fig5-figsupp1-data1.pdf]
