# Supplementary material for: Rad53 checkpoint kinase regulation of DNA replication fork rate via Mrc1 phosphorylation
Source: eLife. 2021 Aug 13;10:e69726. doi: 10.7554/eLife.69726 (PMC8387023; doi:10.7554/eLife.69726)
Supplement: Figure 5—figure supplement 1—source data 2. [file elife-69726-fig5-figsupp1-data2.docx]

16/10/2017 - 12:49:56

peptides generated from sequence-file :

E:\Multipep Spotter09CR-UK\Users\John Diffley\MRC1 protein.SEQ

Nr. Pos. Mol.Weight Sequence Label

1 A 1 2223.8 M-D-D-A-L-H-A-L-S-S-L-T-A-K-K-R-T-T-T-Y

2 A 2 2220.8 D-D-A-L-H-A-L-S-S-L-T-A-K-K-R-T-T-T-Y-K

3 A 3 2233.9 D-A-L-H-A-L-S-S-L-T-A-K-K-R-T-T-T-Y-K-K

4 A 4 2217.9 A-L-H-A-L-S-S-L-T-A-K-K-R-T-T-T-Y-K-K-V

5 A 5 2217.9 L-H-A-L-S-S-L-T-A-K-K-R-T-T-T-Y-K-K-V-A

6 A 6 2203.8 H-A-L-S-S-L-T-A-K-K-R-T-T-T-Y-K-K-V-A-V

7 A 7 2163.8 A-L-S-S-L-T-A-K-K-R-T-T-T-Y-K-K-V-A-V-P

8 A 8 2205.9 L-S-S-L-T-A-K-K-R-T-T-T-Y-K-K-V-A-V-P-I

9 A 9 2205.9 S-S-L-T-A-K-K-R-T-T-T-Y-K-K-V-A-V-P-I-L

10 A10 2233.9 S-L-T-A-K-K-R-T-T-T-Y-K-K-V-A-V-P-I-L-D

11 A11 2275.9 L-T-A-K-K-R-T-T-T-Y-K-K-V-A-V-P-I-L-D-E

12 A12 2276.8 T-A-K-K-R-T-T-T-Y-K-K-V-A-V-P-I-L-D-E-N

13 A13 2290.8 A-K-K-R-T-T-T-Y-K-K-V-A-V-P-I-L-D-E-N-D

14 A14 2333.8 K-K-R-T-T-T-Y-K-K-V-A-V-P-I-L-D-E-N-D-N

15 A15 2306.7 K-R-T-T-T-Y-K-K-V-A-V-P-I-L-D-E-N-D-N-T

16 A16 2292.6 R-T-T-T-Y-K-K-V-A-V-P-I-L-D-E-N-D-N-T-N

17 A17 2193.5 T-T-T-Y-K-K-V-A-V-P-I-L-D-E-N-D-N-T-N-G

18 A18 2206.5 T-T-Y-K-K-V-A-V-P-I-L-D-E-N-D-N-T-N-G-N

19 A19 2162.5 T-Y-K-K-V-A-V-P-I-L-D-E-N-D-N-T-N-G-N-G

20 A20 2158.5 Y-K-K-V-A-V-P-I-L-D-E-N-D-N-T-N-G-N-G-P

21 A21 2109.4 K-K-V-A-V-P-I-L-D-E-N-D-N-T-N-G-N-G-P-N

22 A22 2096.3 K-V-A-V-P-I-L-D-E-N-D-N-T-N-G-N-G-P-N-D

23 A23 2081.3 V-A-V-P-I-L-D-E-N-D-N-T-N-G-N-G-P-N-D-I

24 A24 2097.3 A-V-P-I-L-D-E-N-D-N-T-N-G-N-G-P-N-D-I-D

25 A25 2140.3 V-P-I-L-D-E-N-D-N-T-N-G-N-G-P-N-D-I-D-N

26 A26 2138.3 P-I-L-D-E-N-D-N-T-N-G-N-G-P-N-D-I-D-N-P

27 A27 2138.3 I-L-D-E-N-D-N-T-N-G-N-G-P-N-D-I-D-N-P-P

28 A28 2154.2 L-D-E-N-D-N-T-N-G-N-G-P-N-D-I-D-N-P-P-E

29 A29 2154.2 D-E-N-D-N-T-N-G-N-G-P-N-D-I-D-N-P-P-E-L

30 A30 2140.2 E-N-D-N-T-N-G-N-G-P-N-D-I-D-N-P-P-E-L-T

31 B 1 2068.2 N-D-N-T-N-G-N-G-P-N-D-I-D-N-P-P-E-L-T-G

32 B 2 2068.2 D-N-T-N-G-N-G-P-N-D-I-D-N-P-P-E-L-T-G-N

33 B 3 2010.2 N-T-N-G-N-G-P-N-D-I-D-N-P-P-E-L-T-G-N-G

34 B 4 2043.3 T-N-G-N-G-P-N-D-I-D-N-P-P-E-L-T-G-N-G-F

35 B 5 2055.4 N-G-N-G-P-N-D-I-D-N-P-P-E-L-T-G-N-G-F-L

36 B 6 2088.5 G-N-G-P-N-D-I-D-N-P-P-E-L-T-G-N-G-F-L-F

37 B 7 2102.5 N-G-P-N-D-I-D-N-P-P-E-L-T-G-N-G-F-L-F-A

38 B 8 2102.5 G-P-N-D-I-D-N-P-P-E-L-T-G-N-G-F-L-F-A-N

39 B 9 2116.5 P-N-D-I-D-N-P-P-E-L-T-G-N-G-F-L-F-A-N-A

40 B10 2120.5 N-D-I-D-N-P-P-E-L-T-G-N-G-F-L-F-A-N-A-T

41 B11 2119.6 D-I-D-N-P-P-E-L-T-G-N-G-F-L-F-A-N-A-T-L

42 B12 2118.6 I-D-N-P-P-E-L-T-G-N-G-F-L-F-A-N-A-T-L-N

43 B13 2161.6 D-N-P-P-E-L-T-G-N-G-F-L-F-A-N-A-T-L-N-R

44 B14 2145.6 N-P-P-E-L-T-G-N-G-F-L-F-A-N-A-T-L-N-R-V

45 B15 2159.7 P-P-E-L-T-G-N-G-F-L-F-A-N-A-T-L-N-R-V-K

46 B16 2176.7 P-E-L-T-G-N-G-F-L-F-A-N-A-T-L-N-R-V-K-N

47 B17 2235.8 E-L-T-G-N-G-F-L-F-A-N-A-T-L-N-R-V-K-N-R

48 B18 2219.9 L-T-G-N-G-F-L-F-A-N-A-T-L-N-R-V-K-N-R-L

49 B19 2235.8 T-G-N-G-F-L-F-A-N-A-T-L-N-R-V-K-N-R-L-E

50 B20 2191.8 G-N-G-F-L-F-A-N-A-T-L-N-R-V-K-N-R-L-E-G

51 B21 2262.9 N-G-F-L-F-A-N-A-T-L-N-R-V-K-N-R-L-E-G-K

52 B22 2277 G-F-L-F-A-N-A-T-L-N-R-V-K-N-R-L-E-G-K-K

53 B23 2291 F-L-F-A-N-A-T-L-N-R-V-K-N-R-L-E-G-K-K-A

54 B24 2240.9 L-F-A-N-A-T-L-N-R-V-K-N-R-L-E-G-K-K-A-P

55 B25 2256.8 F-A-N-A-T-L-N-R-V-K-N-R-L-E-G-K-K-A-P-E

56 B26 2237.7 A-N-A-T-L-N-R-V-K-N-R-L-E-G-K-K-A-P-E-Q

57 B27 2280.7 N-A-T-L-N-R-V-K-N-R-L-E-G-K-K-A-P-E-Q-N

58 B28 2303.7 A-T-L-N-R-V-K-N-R-L-E-G-K-K-A-P-E-Q-N-H

59 B29 2346.7 T-L-N-R-V-K-N-R-L-E-G-K-K-A-P-E-Q-N-H-N

60 B30 2359.7 L-N-R-V-K-N-R-L-E-G-K-K-A-P-E-Q-N-H-N-N

61 C 1 2303.6 N-R-V-K-N-R-L-E-G-K-K-A-P-E-Q-N-H-N-N-G

62 C 2 2317.7 R-V-K-N-R-L-E-G-K-K-A-P-E-Q-N-H-N-N-G-K

63 C 3 2276.6 V-K-N-R-L-E-G-K-K-A-P-E-Q-N-H-N-N-G-K-D

64 C 4 2333.7 K-N-R-L-E-G-K-K-A-P-E-Q-N-H-N-N-G-K-D-R

65 C 5 2292.6 N-R-L-E-G-K-K-A-P-E-Q-N-H-N-N-G-K-D-R-S

66 C 6 2307.6 R-L-E-G-K-K-A-P-E-Q-N-H-N-N-G-K-D-R-S-E

67 C 7 2265.5 L-E-G-K-K-A-P-E-Q-N-H-N-N-G-K-D-R-S-E-N

68 C 8 2239.4 E-G-K-K-A-P-E-Q-N-H-N-N-G-K-D-R-S-E-N-S

69 C 9 2223.5 G-K-K-A-P-E-Q-N-H-N-N-G-K-D-R-S-E-N-S-L

70 C10 2263.5 K-K-A-P-E-Q-N-H-N-N-G-K-D-R-S-E-N-S-L-P

71 C11 2236.4 K-A-P-E-Q-N-H-N-N-G-K-D-R-S-E-N-S-L-P-T

72 C12 2236.3 A-P-E-Q-N-H-N-N-G-K-D-R-S-E-N-S-L-P-T-Q

73 C13 2278.4 P-E-Q-N-H-N-N-G-K-D-R-S-E-N-S-L-P-T-Q-L

74 C14 2294.5 E-Q-N-H-N-N-G-K-D-R-S-E-N-S-L-P-T-Q-L-I

75 C15 2252.5 Q-N-H-N-N-G-K-D-R-S-E-N-S-L-P-T-Q-L-I-S

76 C16 2238.5 N-H-N-N-G-K-D-R-S-E-N-S-L-P-T-Q-L-I-S-N

77 C17 2237.6 H-N-N-G-K-D-R-S-E-N-S-L-P-T-Q-L-I-S-N-L

78 C18 2263.7 N-N-G-K-D-R-S-E-N-S-L-P-T-Q-L-I-S-N-L-Y

79 C19 2264.7 N-G-K-D-R-S-E-N-S-L-P-T-Q-L-I-S-N-L-Y-D

80 C20 2207.7 G-K-D-R-S-E-N-S-L-P-T-Q-L-I-S-N-L-Y-D-G

81 C21 2207.7 K-D-R-S-E-N-S-L-P-T-Q-L-I-S-N-L-Y-D-G-G

82 C22 2208.6 D-R-S-E-N-S-L-P-T-Q-L-I-S-N-L-Y-D-G-G-E

83 C23 2222.6 R-S-E-N-S-L-P-T-Q-L-I-S-N-L-Y-D-G-G-E-E

84 C24 2179.6 S-E-N-S-L-P-T-Q-L-I-S-N-L-Y-D-G-G-E-E-L

85 C25 2221.6 E-N-S-L-P-T-Q-L-I-S-N-L-Y-D-G-G-E-E-L-E

86 C26 2220.7 N-S-L-P-T-Q-L-I-S-N-L-Y-D-G-G-E-E-L-E-K

87 C27 2193.7 S-L-P-T-Q-L-I-S-N-L-Y-D-G-G-E-E-L-E-K-S

88 C28 2235.7 L-P-T-Q-L-I-S-N-L-Y-D-G-G-E-E-L-E-K-S-E

89 C29 2221.6 P-T-Q-L-I-S-N-L-Y-D-G-G-E-E-L-E-K-S-E-V

90 C30 2252.7 T-Q-L-I-S-N-L-Y-D-G-G-E-E-L-E-K-S-E-V-K

91 D 1 2266.7 Q-L-I-S-N-L-Y-D-G-G-E-E-L-E-K-S-E-V-K-D

92 D 2 2252.7 L-I-S-N-L-Y-D-G-G-E-E-L-E-K-S-E-V-K-D-N

93 D 3 2226.6 I-S-N-L-Y-D-G-G-E-E-L-E-K-S-E-V-K-D-N-S

94 D 4 2276.6 S-N-L-Y-D-G-G-E-E-L-E-K-S-E-V-K-D-N-S-Y

95 D 5 2276.6 N-L-Y-D-G-G-E-E-L-E-K-S-E-V-K-D-N-S-Y-S

96 D 6 2291.6 L-Y-D-G-G-E-E-L-E-K-S-E-V-K-D-N-S-Y-S-E

97 D 7 2306.6 Y-D-G-G-E-E-L-E-K-S-E-V-K-D-N-S-Y-S-E-K

98 D 8 2257.5 D-G-G-E-E-L-E-K-S-E-V-K-D-N-S-Y-S-E-K-N

99 D 9 2241.5 G-G-E-E-L-E-K-S-E-V-K-D-N-S-Y-S-E-K-N-V

100 D10 2271.5 G-E-E-L-E-K-S-E-V-K-D-N-S-Y-S-E-K-N-V-S

101 D11 2301.5 E-E-L-E-K-S-E-V-K-D-N-S-Y-S-E-K-N-V-S-S

102 D12 2259.5 E-L-E-K-S-E-V-K-D-N-S-Y-S-E-K-N-V-S-S-S

103 D13 2277.6 L-E-K-S-E-V-K-D-N-S-Y-S-E-K-N-V-S-S-S-F

104 D14 2265.5 E-K-S-E-V-K-D-N-S-Y-S-E-K-N-V-S-S-S-F-T

105 D15 2264.5 K-S-E-V-K-D-N-S-Y-S-E-K-N-V-S-S-S-F-T-Q

106 D16 2237.4 S-E-V-K-D-N-S-Y-S-E-K-N-V-S-S-S-F-T-Q-T

107 D17 2278.4 E-V-K-D-N-S-Y-S-E-K-N-V-S-S-S-F-T-Q-T-Q

108 D18 2305.5 V-K-D-N-S-Y-S-E-K-N-V-S-S-S-F-T-Q-T-Q-R

109 D19 2319.6 K-D-N-S-Y-S-E-K-N-V-S-S-S-F-T-Q-T-Q-R-I

110 D20 2288.5 D-N-S-Y-S-E-K-N-V-S-S-S-F-T-Q-T-Q-R-I-P

111 D21 2272.5 N-S-Y-S-E-K-N-V-S-S-S-F-T-Q-T-Q-R-I-P-V

112 D22 2245.5 S-Y-S-E-K-N-V-S-S-S-F-T-Q-T-Q-R-I-P-V-S

113 D23 2271.6 Y-S-E-K-N-V-S-S-S-F-T-Q-T-Q-R-I-P-V-S-I

114 D24 2236.5 S-E-K-N-V-S-S-S-F-T-Q-T-Q-R-I-P-V-S-I-Q

115 D25 2277.5 E-K-N-V-S-S-S-F-T-Q-T-Q-R-I-P-V-S-I-Q-Q

116 D26 2263.5 K-N-V-S-S-S-F-T-Q-T-Q-R-I-P-V-S-I-Q-Q-D

117 D27 2263.5 N-V-S-S-S-F-T-Q-T-Q-R-I-P-V-S-I-Q-Q-D-K

118 D28 2248.5 V-S-S-S-F-T-Q-T-Q-R-I-P-V-S-I-Q-Q-D-K-V

119 D29 2296.6 S-S-S-F-T-Q-T-Q-R-I-P-V-S-I-Q-Q-D-K-V-F

120 D30 2323.6 S-S-F-T-Q-T-Q-R-I-P-V-S-I-Q-Q-D-K-V-F-N

121 E 1 2335.6 S-F-T-Q-T-Q-R-I-P-V-S-I-Q-Q-D-K-V-F-N-V

122 E 2 2345.6 F-T-Q-T-Q-R-I-P-V-S-I-Q-Q-D-K-V-F-N-V-P

123 E 3 2311.6 T-Q-T-Q-R-I-P-V-S-I-Q-Q-D-K-V-F-N-V-P-I

124 E 4 2347.6 Q-T-Q-R-I-P-V-S-I-Q-Q-D-K-V-F-N-V-P-I-H

125 E 5 2306.6 T-Q-R-I-P-V-S-I-Q-Q-D-K-V-F-N-V-P-I-H-S

126 E 6 2304.6 Q-R-I-P-V-S-I-Q-Q-D-K-V-F-N-V-P-I-H-S-V

127 E 7 2290.6 R-I-P-V-S-I-Q-Q-D-K-V-F-N-V-P-I-H-S-V-N

128 E 8 2249.5 I-P-V-S-I-Q-Q-D-K-V-F-N-V-P-I-H-S-V-N-D

129 E 9 2193.4 P-V-S-I-Q-Q-D-K-V-F-N-V-P-I-H-S-V-N-D-G

130 E10 2224.5 V-S-I-Q-Q-D-K-V-F-N-V-P-I-H-S-V-N-D-G-K

131 E11 2222.5 S-I-Q-Q-D-K-V-F-N-V-P-I-H-S-V-N-D-G-K-P

132 E12 2236.5 I-Q-Q-D-K-V-F-N-V-P-I-H-S-V-N-D-G-K-P-T

133 E13 2251.4 Q-Q-D-K-V-F-N-V-P-I-H-S-V-N-D-G-K-P-T-Q

134 E14 2236.5 Q-D-K-V-F-N-V-P-I-H-S-V-N-D-G-K-P-T-Q-L

135 E15 2221.6 D-K-V-F-N-V-P-I-H-S-V-N-D-G-K-P-T-Q-L-I

136 E16 2234.7 K-V-F-N-V-P-I-H-S-V-N-D-G-K-P-T-Q-L-I-K

137 E17 2235.6 V-F-N-V-P-I-H-S-V-N-D-G-K-P-T-Q-L-I-K-E

138 E18 2251.6 F-N-V-P-I-H-S-V-N-D-G-K-P-T-Q-L-I-K-E-D

139 E19 2161.5 N-V-P-I-H-S-V-N-D-G-K-P-T-Q-L-I-K-E-D-G

140 E20 2160.6 V-P-I-H-S-V-N-D-G-K-P-T-Q-L-I-K-E-D-G-L

141 E21 2160.6 P-I-H-S-V-N-D-G-K-P-T-Q-L-I-K-E-D-G-L-V

142 E22 2177.6 I-H-S-V-N-D-G-K-P-T-Q-L-I-K-E-D-G-L-V-N

143 E23 2193.5 H-S-V-N-D-G-K-P-T-Q-L-I-K-E-D-G-L-V-N-E

144 E24 2157.5 S-V-N-D-G-K-P-T-Q-L-I-K-E-D-G-L-V-N-E-T

145 E25 2157.5 V-N-D-G-K-P-T-Q-L-I-K-E-D-G-L-V-N-E-T-S

146 E26 2186.5 N-D-G-K-P-T-Q-L-I-K-E-D-G-L-V-N-E-T-S-Q

147 E27 2143.5 D-G-K-P-T-Q-L-I-K-E-D-G-L-V-N-E-T-S-Q-A

148 E28 2141.6 G-K-P-T-Q-L-I-K-E-D-G-L-V-N-E-T-S-Q-A-L

149 E29 2212.7 K-P-T-Q-L-I-K-E-D-G-L-V-N-E-T-S-Q-A-L-K

150 E30 2185.6 P-T-Q-L-I-K-E-D-G-L-V-N-E-T-S-Q-A-L-K-T

151 F 1 2185.6 T-Q-L-I-K-E-D-G-L-V-N-E-T-S-Q-A-L-K-T-P

152 F 2 2197.7 Q-L-I-K-E-D-G-L-V-N-E-T-S-Q-A-L-K-T-P-L

153 F 3 2170.7 L-I-K-E-D-G-L-V-N-E-T-S-Q-A-L-K-T-P-L-T

154 F 4 2158.6 I-K-E-D-G-L-V-N-E-T-S-Q-A-L-K-T-P-L-T-T

155 F 5 2102.5 K-E-D-G-L-V-N-E-T-S-Q-A-L-K-T-P-L-T-T-G

156 F 6 2130.5 E-D-G-L-V-N-E-T-S-Q-A-L-K-T-P-L-T-T-G-R

157 F 7 2098.5 D-G-L-V-N-E-T-S-Q-A-L-K-T-P-L-T-T-G-R-P

158 F 8 2040.5 G-L-V-N-E-T-S-Q-A-L-K-T-P-L-T-T-G-R-P-G

159 F 9 2054.5 L-V-N-E-T-S-Q-A-L-K-T-P-L-T-T-G-R-P-G-A

160 F10 2042.4 V-N-E-T-S-Q-A-L-K-T-P-L-T-T-G-R-P-G-A-T

161 F11 2071.4 N-E-T-S-Q-A-L-K-T-P-L-T-T-G-R-P-G-A-T-Q

162 F12 2113.5 E-T-S-Q-A-L-K-T-P-L-T-T-G-R-P-G-A-T-Q-R

163 F13 2097.6 T-S-Q-A-L-K-T-P-L-T-T-G-R-P-G-A-T-Q-R-I

164 F14 2111.6 S-Q-A-L-K-T-P-L-T-T-G-R-P-G-A-T-Q-R-I-D

165 F15 2111.6 Q-A-L-K-T-P-L-T-T-G-R-P-G-A-T-Q-R-I-D-S

166 F16 2070.6 A-L-K-T-P-L-T-T-G-R-P-G-A-T-Q-R-I-D-S-S

167 F17 2056.6 L-K-T-P-L-T-T-G-R-P-G-A-T-Q-R-I-D-S-S-G

168 F18 2014.5 K-T-P-L-T-T-G-R-P-G-A-T-Q-R-I-D-S-S-G-A

169 F19 1987.4 T-P-L-T-T-G-R-P-G-A-T-Q-R-I-D-S-S-G-A-T

170 F20 1973.4 P-L-T-T-G-R-P-G-A-T-Q-R-I-D-S-S-G-A-T-S

171 F21 2004.4 L-T-T-G-R-P-G-A-T-Q-R-I-D-S-S-G-A-T-S-Q

172 F22 1992.3 T-T-G-R-P-G-A-T-Q-R-I-D-S-S-G-A-T-S-Q-T

173 F23 2019.3 T-G-R-P-G-A-T-Q-R-I-D-S-S-G-A-T-S-Q-T-Q

174 F24 2015.3 G-R-P-G-A-T-Q-R-I-D-S-S-G-A-T-S-Q-T-Q-P

175 F25 2071.4 R-P-G-A-T-Q-R-I-D-S-S-G-A-T-S-Q-T-Q-P-I

176 F26 2043.4 P-G-A-T-Q-R-I-D-S-S-G-A-T-S-Q-T-Q-P-I-K

177 F27 2033.4 G-A-T-Q-R-I-D-S-S-G-A-T-S-Q-T-Q-P-I-K-S

178 F28 2089.5 A-T-Q-R-I-D-S-S-G-A-T-S-Q-T-Q-P-I-K-S-I

179 F29 2147.5 T-Q-R-I-D-S-S-G-A-T-S-Q-T-Q-P-I-K-S-I-E

180 F30 2143.5 Q-R-I-D-S-S-G-A-T-S-Q-T-Q-P-I-K-S-I-E-P

181 G 1 2143.5 R-I-D-S-S-G-A-T-S-Q-T-Q-P-I-K-S-I-E-P-Q

182 G 2 2074.4 I-D-S-S-G-A-T-S-Q-T-Q-P-I-K-S-I-E-P-Q-S

183 G 3 2089.3 D-S-S-G-A-T-S-Q-T-Q-P-I-K-S-I-E-P-Q-S-Q

184 G 4 2087.4 S-S-G-A-T-S-Q-T-Q-P-I-K-S-I-E-P-Q-S-Q-I

185 G 5 2113.5 S-G-A-T-S-Q-T-Q-P-I-K-S-I-E-P-Q-S-Q-I-I

186 G 6 2127.5 G-A-T-S-Q-T-Q-P-I-K-S-I-E-P-Q-S-Q-I-I-T

187 G 7 2171.5 A-T-S-Q-T-Q-P-I-K-S-I-E-P-Q-S-Q-I-I-T-T

188 G 8 2187.5 T-S-Q-T-Q-P-I-K-S-I-E-P-Q-S-Q-I-I-T-T-S

189 G 9 2173.5 S-Q-T-Q-P-I-K-S-I-E-P-Q-S-Q-I-I-T-T-S-S

190 G10 2200.5 Q-T-Q-P-I-K-S-I-E-P-Q-S-Q-I-I-T-T-S-S-N

191 G11 2209.5 T-Q-P-I-K-S-I-E-P-Q-S-Q-I-I-T-T-S-S-N-H

192 G12 2195.5 Q-P-I-K-S-I-E-P-Q-S-Q-I-I-T-T-S-S-N-H-S

193 G13 2181.5 P-I-K-S-I-E-P-Q-S-Q-I-I-T-T-S-S-N-H-S-N

194 G14 2155.5 I-K-S-I-E-P-Q-S-Q-I-I-T-T-S-S-N-H-S-N-A

195 G15 2155.5 K-S-I-E-P-Q-S-Q-I-I-T-T-S-S-N-H-S-N-A-L

196 G16 2114.4 S-I-E-P-Q-S-Q-I-I-T-T-S-S-N-H-S-N-A-L-S

197 G17 2124.4 I-E-P-Q-S-Q-I-I-T-T-S-S-N-H-S-N-A-L-S-P

198 G18 2139.4 E-P-Q-S-Q-I-I-T-T-S-S-N-H-S-N-A-L-S-P-K

199 G19 2123.5 P-Q-S-Q-I-I-T-T-S-S-N-H-S-N-A-L-S-P-K-I

200 G20 2123.5 Q-S-Q-I-I-T-T-S-S-N-H-S-N-A-L-S-P-K-I-P

201 G21 2108.6 S-Q-I-I-T-T-S-S-N-H-S-N-A-L-S-P-K-I-P-I

202 G22 2134.7 Q-I-I-T-T-S-S-N-H-S-N-A-L-S-P-K-I-P-I-I

203 G23 2103.7 I-I-T-T-S-S-N-H-S-N-A-L-S-P-K-I-P-I-I-P

204 G24 2091.6 I-T-T-S-S-N-H-S-N-A-L-S-P-K-I-P-I-I-P-T

205 G25 2107.5 T-T-S-S-N-H-S-N-A-L-S-P-K-I-P-I-I-P-T-E

206 G26 2119.6 T-S-S-N-H-S-N-A-L-S-P-K-I-P-I-I-P-T-E-L

207 G27 2131.7 S-S-N-H-S-N-A-L-S-P-K-I-P-I-I-P-T-E-L-I

208 G28 2101.7 S-N-H-S-N-A-L-S-P-K-I-P-I-I-P-T-E-L-I-G

209 G29 2115.7 N-H-S-N-A-L-S-P-K-I-P-I-I-P-T-E-L-I-G-T

210 G30 2088.7 H-S-N-A-L-S-P-K-I-P-I-I-P-T-E-L-I-G-T-S

211 H 1 2048.7 S-N-A-L-S-P-K-I-P-I-I-P-T-E-L-I-G-T-S-P

212 H 2 2074.8 N-A-L-S-P-K-I-P-I-I-P-T-E-L-I-G-T-S-P-L

213 H 3 2107.9 A-L-S-P-K-I-P-I-I-P-T-E-L-I-G-T-S-P-L-F

214 H 4 2164.9 L-S-P-K-I-P-I-I-P-T-E-L-I-G-T-S-P-L-F-Q

215 H 5 2138.8 S-P-K-I-P-I-I-P-T-E-L-I-G-T-S-P-L-F-Q-S

216 H 6 2164.9 P-K-I-P-I-I-P-T-E-L-I-G-T-S-P-L-F-Q-S-I

217 H 7 2195.9 K-I-P-I-I-P-T-E-L-I-G-T-S-P-L-F-Q-S-I-Q

218 H 8 2181.8 I-P-I-I-P-T-E-L-I-G-T-S-P-L-F-Q-S-I-Q-N

219 H 9 2224.8 P-I-I-P-T-E-L-I-G-T-S-P-L-F-Q-S-I-Q-N-R

220 H10 2184.8 I-I-P-T-E-L-I-G-T-S-P-L-F-Q-S-I-Q-N-R-G

221 H11 2168.7 I-P-T-E-L-I-G-T-S-P-L-F-Q-S-I-Q-N-R-G-P

222 H12 2170.6 P-T-E-L-I-G-T-S-P-L-F-Q-S-I-Q-N-R-G-P-D

223 H13 2174.6 T-E-L-I-G-T-S-P-L-F-Q-S-I-Q-N-R-G-P-D-T

224 H14 2201.6 E-L-I-G-T-S-P-L-F-Q-S-I-Q-N-R-G-P-D-T-Q

225 H15 2203.7 L-I-G-T-S-P-L-F-Q-S-I-Q-N-R-G-P-D-T-Q-M

226 H16 2205.6 I-G-T-S-P-L-F-Q-S-I-Q-N-R-G-P-D-T-Q-M-D

227 H17 2191.5 G-T-S-P-L-F-Q-S-I-Q-N-R-G-P-D-T-Q-M-D-V

228 H18 2231.5 T-S-P-L-F-Q-S-I-Q-N-R-G-P-D-T-Q-M-D-V-P

229 H19 2227.5 S-P-L-F-Q-S-I-Q-N-R-G-P-D-T-Q-M-D-V-P-P

230 H20 2268.5 P-L-F-Q-S-I-Q-N-R-G-P-D-T-Q-M-D-V-P-P-Q

231 H21 2272.5 L-F-Q-S-I-Q-N-R-G-P-D-T-Q-M-D-V-P-P-Q-T

232 H22 2230.4 F-Q-S-I-Q-N-R-G-P-D-T-Q-M-D-V-P-P-Q-T-A

233 H23 2220.3 Q-S-I-Q-N-R-G-P-D-T-Q-M-D-V-P-P-Q-T-A-H

234 H24 2207.3 S-I-Q-N-R-G-P-D-T-Q-M-D-V-P-P-Q-T-A-H-D

235 H25 2249.3 I-Q-N-R-G-P-D-T-Q-M-D-V-P-P-Q-T-A-H-D-E

236 H26 2251.2 Q-N-R-G-P-D-T-Q-M-D-V-P-P-Q-T-A-H-D-E-D

237 H27 2251.3 N-R-G-P-D-T-Q-M-D-V-P-P-Q-T-A-H-D-E-D-K

238 H28 2238.3 R-G-P-D-T-Q-M-D-V-P-P-Q-T-A-H-D-E-D-K-T

239 H29 2210.2 G-P-D-T-Q-M-D-V-P-P-Q-T-A-H-D-E-D-K-T-Q

240 H30 2224.2 P-D-T-Q-M-D-V-P-P-Q-T-A-H-D-E-D-K-T-Q-A

241 I 1 2240.3 D-T-Q-M-D-V-P-P-Q-T-A-H-D-E-D-K-T-Q-A-I

242 I 2 2182.3 T-Q-M-D-V-P-P-Q-T-A-H-D-E-D-K-T-Q-A-I-G

243 I 3 2194.4 Q-M-D-V-P-P-Q-T-A-H-D-E-D-K-T-Q-A-I-G-I

244 I 4 2163.4 M-D-V-P-P-Q-T-A-H-D-E-D-K-T-Q-A-I-G-I-P

245 I 5 2160.3 D-V-P-P-Q-T-A-H-D-E-D-K-T-Q-A-I-G-I-P-Q

246 I 6 2116.3 V-P-P-Q-T-A-H-D-E-D-K-T-Q-A-I-G-I-P-Q-A

247 I 7 2118.3 P-P-Q-T-A-H-D-E-D-K-T-Q-A-I-G-I-P-Q-A-T

248 I 8 2158.3 P-Q-T-A-H-D-E-D-K-T-Q-A-I-G-I-P-Q-A-T-H

249 I 9 2189.3 Q-T-A-H-D-E-D-K-T-Q-A-I-G-I-P-Q-A-T-H-Q

250 I10 2190.3 T-A-H-D-E-D-K-T-Q-A-I-G-I-P-Q-A-T-H-Q-E

251 I11 2217.3 A-H-D-E-D-K-T-Q-A-I-G-I-P-Q-A-T-H-Q-E-Q

252 I12 2274.4 H-D-E-D-K-T-Q-A-I-G-I-P-Q-A-T-H-Q-E-Q-K

253 I13 2238.4 D-E-D-K-T-Q-A-I-G-I-P-Q-A-T-H-Q-E-Q-K-T

254 I14 2251.4 E-D-K-T-Q-A-I-G-I-P-Q-A-T-H-Q-E-Q-K-T-Q

255 I15 2235.5 D-K-T-Q-A-I-G-I-P-Q-A-T-H-Q-E-Q-K-T-Q-I

256 I16 2235.5 K-T-Q-A-I-G-I-P-Q-A-T-H-Q-E-Q-K-T-Q-I-D

257 I17 2208.4 T-Q-A-I-G-I-P-Q-A-T-H-Q-E-Q-K-T-Q-I-D-T

258 I18 2206.4 Q-A-I-G-I-P-Q-A-T-H-Q-E-Q-K-T-Q-I-D-T-V

259 I19 2149.4 A-I-G-I-P-Q-A-T-H-Q-E-Q-K-T-Q-I-D-T-V-A

260 I20 2206.4 I-G-I-P-Q-A-T-H-Q-E-Q-K-T-Q-I-D-T-V-A-Q

261 I21 2194.3 G-I-P-Q-A-T-H-Q-E-Q-K-T-Q-I-D-T-V-A-Q-T

262 I22 2250.4 I-P-Q-A-T-H-Q-E-Q-K-T-Q-I-D-T-V-A-Q-T-L

263 I23 2265.3 P-Q-A-T-H-Q-E-Q-K-T-Q-I-D-T-V-A-Q-T-L-Q

264 I24 2283.3 Q-A-T-H-Q-E-Q-K-T-Q-I-D-T-V-A-Q-T-L-Q-D

265 I25 2284.3 A-T-H-Q-E-Q-K-T-Q-I-D-T-V-A-Q-T-L-Q-D-E

266 I26 2312.3 T-H-Q-E-Q-K-T-Q-I-D-T-V-A-Q-T-L-Q-D-E-V

267 I27 2308.3 H-Q-E-Q-K-T-Q-I-D-T-V-A-Q-T-L-Q-D-E-V-P

268 I28 2308.3 Q-E-Q-K-T-Q-I-D-T-V-A-Q-T-L-Q-D-E-V-P-H

269 I29 2281.3 E-Q-K-T-Q-I-D-T-V-A-Q-T-L-Q-D-E-V-P-H-T

270 I30 2265.4 Q-K-T-Q-I-D-T-V-A-Q-T-L-Q-D-E-V-P-H-T-L

271 J 1 2265.5 K-T-Q-I-D-T-V-A-Q-T-L-Q-D-E-V-P-H-T-L-K

272 J 2 2250.5 T-Q-I-D-T-V-A-Q-T-L-Q-D-E-V-P-H-T-L-K-I

273 J 3 2305.6 Q-I-D-T-V-A-Q-T-L-Q-D-E-V-P-H-T-L-K-I-R

274 J 4 2306.6 I-D-T-V-A-Q-T-L-Q-D-E-V-P-H-T-L-K-I-R-E

275 J 5 2306.6 D-T-V-A-Q-T-L-Q-D-E-V-P-H-T-L-K-I-R-E-I

276 J 6 2319.6 T-V-A-Q-T-L-Q-D-E-V-P-H-T-L-K-I-R-E-I-Q

277 J 7 2305.6 V-A-Q-T-L-Q-D-E-V-P-H-T-L-K-I-R-E-I-Q-S

278 J 8 2335.6 A-Q-T-L-Q-D-E-V-P-H-T-L-K-I-R-E-I-Q-S-E

279 J 9 2377.7 Q-T-L-Q-D-E-V-P-H-T-L-K-I-R-E-I-Q-S-E-L

280 J10 2320.7 T-L-Q-D-E-V-P-H-T-L-K-I-R-E-I-Q-S-E-L-A

281 J11 2306.7 L-Q-D-E-V-P-H-T-L-K-I-R-E-I-Q-S-E-L-A-S

282 J12 2322.6 Q-D-E-V-P-H-T-L-K-I-R-E-I-Q-S-E-L-A-S-E

283 J13 2309.6 D-E-V-P-H-T-L-K-I-R-E-I-Q-S-E-L-A-S-E-D

284 J14 2281.6 E-V-P-H-T-L-K-I-R-E-I-Q-S-E-L-A-S-E-D-S

285 J15 2280.7 V-P-H-T-L-K-I-R-E-I-Q-S-E-L-A-S-E-D-S-K

286 J16 2337.8 P-H-T-L-K-I-R-E-I-Q-S-E-L-A-S-E-D-S-K-R

287 J17 2369.8 H-T-L-K-I-R-E-I-Q-S-E-L-A-S-E-D-S-K-R-E

288 J18 2360.9 T-L-K-I-R-E-I-Q-S-E-L-A-S-E-D-S-K-R-E-K

289 J19 2330.9 L-K-I-R-E-I-Q-S-E-L-A-S-E-D-S-K-R-E-K-A

290 J20 2373.9 K-I-R-E-I-Q-S-E-L-A-S-E-D-S-K-R-E-K-A-R

291 J21 2359.8 I-R-E-I-Q-S-E-L-A-S-E-D-S-K-R-E-K-A-R-N

292 J22 2345.7 R-E-I-Q-S-E-L-A-S-E-D-S-K-R-E-K-A-R-N-V

293 J23 2318.6 E-I-Q-S-E-L-A-S-E-D-S-K-R-E-K-A-R-N-V-E

294 J24 2352.7 I-Q-S-E-L-A-S-E-D-S-K-R-E-K-A-R-N-V-E-Y

295 J25 2367.7 Q-S-E-L-A-S-E-D-S-K-R-E-K-A-R-N-V-E-Y-K

296 J26 2367.8 S-E-L-A-S-E-D-S-K-R-E-K-A-R-N-V-E-Y-K-K

297 J27 2377.8 E-L-A-S-E-D-S-K-R-E-K-A-R-N-V-E-Y-K-K-P

298 J28 2376.8 L-A-S-E-D-S-K-R-E-K-A-R-N-V-E-Y-K-K-P-Q

299 J29 2391.8 A-S-E-D-S-K-R-E-K-A-R-N-V-E-Y-K-K-P-Q-K

300 J30 2417.8 S-E-D-S-K-R-E-K-A-R-N-V-E-Y-K-K-P-Q-K-P

301 K 1 2443.9 E-D-S-K-R-E-K-A-R-N-V-E-Y-K-K-P-Q-K-P-I

302 K 2 2411.9 D-S-K-R-E-K-A-R-N-V-E-Y-K-K-P-Q-K-P-I-P

303 K 3 2397.9 S-K-R-E-K-A-R-N-V-E-Y-K-K-P-Q-K-P-I-P-T

304 K 4 2439 K-R-E-K-A-R-N-V-E-Y-K-K-P-Q-K-P-I-P-T-K

305 K 5 2439 R-E-K-A-R-N-V-E-Y-K-K-P-Q-K-P-I-P-T-K-K

306 K 6 2430 E-K-A-R-N-V-E-Y-K-K-P-Q-K-P-I-P-T-K-K-F

307 K 7 2448.1 K-A-R-N-V-E-Y-K-K-P-Q-K-P-I-P-T-K-K-F-F

308 K 8 2407 A-R-N-V-E-Y-K-K-P-Q-K-P-I-P-T-K-K-F-F-S

309 K 9 2464.1 R-N-V-E-Y-K-K-P-Q-K-P-I-P-T-K-K-F-F-S-K

310 K10 2437 N-V-E-Y-K-K-P-Q-K-P-I-P-T-K-K-F-F-S-K-E

311 K11 2410 V-E-Y-K-K-P-Q-K-P-I-P-T-K-K-F-F-S-K-E-S

312 K12 2458.1 E-Y-K-K-P-Q-K-P-I-P-T-K-K-F-F-S-K-E-S-F

313 K13 2442.2 Y-K-K-P-Q-K-P-I-P-T-K-K-F-F-S-K-E-S-F-L

314 K14 2350.1 K-K-P-Q-K-P-I-P-T-K-K-F-F-S-K-E-S-F-L-A

315 K15 2337 K-P-Q-K-P-I-P-T-K-K-F-F-S-K-E-S-F-L-A-D

316 K16 2356 P-Q-K-P-I-P-T-K-K-F-F-S-K-E-S-F-L-A-D-F

317 K17 2374 Q-K-P-I-P-T-K-K-F-F-S-K-E-S-F-L-A-D-F-D

318 K18 2361 K-P-I-P-T-K-K-F-F-S-K-E-S-F-L-A-D-F-D-D

319 K19 2319.9 P-I-P-T-K-K-F-F-S-K-E-S-F-L-A-D-F-D-D-S

320 K20 2309.9 I-P-T-K-K-F-F-S-K-E-S-F-L-A-D-F-D-D-S-S

321 K21 2283.8 P-T-K-K-F-F-S-K-E-S-F-L-A-D-F-D-D-S-S-S

322 K22 2300.8 T-K-K-F-F-S-K-E-S-F-L-A-D-F-D-D-S-S-S-N

323 K23 2328.8 K-K-F-F-S-K-E-S-F-L-A-D-F-D-D-S-S-S-N-E

324 K24 2315.7 K-F-F-S-K-E-S-F-L-A-D-F-D-D-S-S-S-N-E-D

325 K25 2302.6 F-F-S-K-E-S-F-L-A-D-F-D-D-S-S-S-N-E-D-D

326 K26 2270.5 F-S-K-E-S-F-L-A-D-F-D-D-S-S-S-N-E-D-D-D

327 K27 2236.5 S-K-E-S-F-L-A-D-F-D-D-S-S-S-N-E-D-D-D-I

328 K28 2277.6 K-E-S-F-L-A-D-F-D-D-S-S-S-N-E-D-D-D-I-K

329 K29 2262.6 E-S-F-L-A-D-F-D-D-S-S-S-N-E-D-D-D-I-K-L

330 K30 2262.6 S-F-L-A-D-F-D-D-S-S-S-N-E-D-D-D-I-K-L-E

331 L 1 2289.6 F-L-A-D-F-D-D-S-S-S-N-E-D-D-D-I-K-L-E-N

332 L 2 2213.5 L-A-D-F-D-D-S-S-S-N-E-D-D-D-I-K-L-E-N-A

333 L 3 2237.4 A-D-F-D-D-S-S-S-N-E-D-D-D-I-K-L-E-N-A-H

334 L 4 2263.4 D-F-D-D-S-S-S-N-E-D-D-D-I-K-L-E-N-A-H-P

335 L 5 2276.5 F-D-D-S-S-S-N-E-D-D-D-I-K-L-E-N-A-H-P-K

336 L 6 2226.4 D-D-S-S-S-N-E-D-D-D-I-K-L-E-N-A-H-P-K-P

337 L 7 2210.4 D-S-S-S-N-E-D-D-D-I-K-L-E-N-A-H-P-K-P-V

338 L 8 2223.4 S-S-S-N-E-D-D-D-I-K-L-E-N-A-H-P-K-P-V-Q

339 L 9 2250.4 S-S-N-E-D-D-D-I-K-L-E-N-A-H-P-K-P-V-Q-N

340 L10 2278.4 S-N-E-D-D-D-I-K-L-E-N-A-H-P-K-P-V-Q-N-D

341 L11 2306.4 N-E-D-D-D-I-K-L-E-N-A-H-P-K-P-V-Q-N-D-D

342 L12 2321.4 E-D-D-D-I-K-L-E-N-A-H-P-K-P-V-Q-N-D-D-E

343 L13 2305.5 D-D-D-I-K-L-E-N-A-H-P-K-P-V-Q-N-D-D-E-L

344 L14 2327.5 D-D-I-K-L-E-N-A-H-P-K-P-V-Q-N-D-D-E-L-H

345 L15 2341.5 D-I-K-L-E-N-A-H-P-K-P-V-Q-N-D-D-E-L-H-E

346 L16 2340.5 I-K-L-E-N-A-H-P-K-P-V-Q-N-D-D-E-L-H-E-N

347 L17 2355.5 K-L-E-N-A-H-P-K-P-V-Q-N-D-D-E-L-H-E-N-K

348 L18 2314.4 L-E-N-A-H-P-K-P-V-Q-N-D-D-E-L-H-E-N-K-S

349 L19 2300.3 E-N-A-H-P-K-P-V-Q-N-D-D-E-L-H-E-N-K-S-V

350 L20 2300.3 N-A-H-P-K-P-V-Q-N-D-D-E-L-H-E-N-K-S-V-E

351 L21 2299.4 A-H-P-K-P-V-Q-N-D-D-E-L-H-E-N-K-S-V-E-L

352 L22 2342.4 H-P-K-P-V-Q-N-D-D-E-L-H-E-N-K-S-V-E-L-N

353 L23 2318.5 P-K-P-V-Q-N-D-D-E-L-H-E-N-K-S-V-E-L-N-L

354 L24 2322.5 K-P-V-Q-N-D-D-E-L-H-E-N-K-S-V-E-L-N-L-T

355 L25 2309.4 P-V-Q-N-D-D-E-L-H-E-N-K-S-V-E-L-N-L-T-D

356 L26 2341.4 V-Q-N-D-D-E-L-H-E-N-K-S-V-E-L-N-L-T-D-E

357 L27 2343.4 Q-N-D-D-E-L-H-E-N-K-S-V-E-L-N-L-T-D-E-T

358 L28 2371.5 N-D-D-E-L-H-E-N-K-S-V-E-L-N-L-T-D-E-T-R

359 L29 2370.6 D-D-E-L-H-E-N-K-S-V-E-L-N-L-T-D-E-T-R-I

360 L30 2369.6 D-E-L-H-E-N-K-S-V-E-L-N-L-T-D-E-T-R-I-N

361 M 1 2383.6 E-L-H-E-N-K-S-V-E-L-N-L-T-D-E-T-R-I-N-E

362 M 2 2382.7 L-H-E-N-K-S-V-E-L-N-L-T-D-E-T-R-I-N-E-K

363 M 3 2425.7 H-E-N-K-S-V-E-L-N-L-T-D-E-T-R-I-N-E-K-R

364 M 4 2387.7 E-N-K-S-V-E-L-N-L-T-D-E-T-R-I-N-E-K-R-V

365 M 5 2355.7 N-K-S-V-E-L-N-L-T-D-E-T-R-I-N-E-K-R-V-P

366 M 6 2354.8 K-S-V-E-L-N-L-T-D-E-T-R-I-N-E-K-R-V-P-L

367 M 7 2339.8 S-V-E-L-N-L-T-D-E-T-R-I-N-E-K-R-V-P-L-L

368 M 8 2339.8 V-E-L-N-L-T-D-E-T-R-I-N-E-K-R-V-P-L-L-S

369 M 9 2327.8 E-L-N-L-T-D-E-T-R-I-N-E-K-R-V-P-L-L-S-S

370 M10 2361.9 L-N-L-T-D-E-T-R-I-N-E-K-R-V-P-L-L-S-S-Y

371 M11 2319.8 N-L-T-D-E-T-R-I-N-E-K-R-V-P-L-L-S-S-Y-A

372 M12 2319.8 L-T-D-E-T-R-I-N-E-K-R-V-P-L-L-S-S-Y-A-N

373 M13 2320.7 T-D-E-T-R-I-N-E-K-R-V-P-L-L-S-S-Y-A-N-N

374 M14 2332.8 D-E-T-R-I-N-E-K-R-V-P-L-L-S-S-Y-A-N-N-L

375 M15 2345.9 E-T-R-I-N-E-K-R-V-P-L-L-S-S-Y-A-N-N-L-K

376 M16 2373 T-R-I-N-E-K-R-V-P-L-L-S-S-Y-A-N-N-L-K-R

377 M17 2401 R-I-N-E-K-R-V-P-L-L-S-S-Y-A-N-N-L-K-R-E

378 M18 2358 I-N-E-K-R-V-P-L-L-S-S-Y-A-N-N-L-K-R-E-I

379 M19 2359.9 N-E-K-R-V-P-L-L-S-S-Y-A-N-N-L-K-R-E-I-D

380 M20 2332.9 E-K-R-V-P-L-L-S-S-Y-A-N-N-L-K-R-E-I-D-S

381 M21 2290.9 K-R-V-P-L-L-S-S-Y-A-N-N-L-K-R-E-I-D-S-S

382 M22 2290.9 R-V-P-L-L-S-S-Y-A-N-N-L-K-R-E-I-D-S-S-K

383 M23 2237.8 V-P-L-L-S-S-Y-A-N-N-L-K-R-E-I-D-S-S-K-C

384 M24 2251.9 P-L-L-S-S-Y-A-N-N-L-K-R-E-I-D-S-S-K-C-I

385 M25 2255.9 L-L-S-S-Y-A-N-N-L-K-R-E-I-D-S-S-K-C-I-T

386 M26 2255.9 L-S-S-Y-A-N-N-L-K-R-E-I-D-S-S-K-C-I-T-L

387 M27 2257.8 S-S-Y-A-N-N-L-K-R-E-I-D-S-S-K-C-I-T-L-D

388 M28 2283.9 S-Y-A-N-N-L-K-R-E-I-D-S-S-K-C-I-T-L-D-L

389 M29 2311.9 Y-A-N-N-L-K-R-E-I-D-S-S-K-C-I-T-L-D-L-D

390 M30 2235.8 A-N-N-L-K-R-E-I-D-S-S-K-C-I-T-L-D-L-D-S

391 N 1 2279.8 N-N-L-K-R-E-I-D-S-S-K-C-I-T-L-D-L-D-S-D

392 N 2 2252.8 N-L-K-R-E-I-D-S-S-K-C-I-T-L-D-L-D-S-D-S

393 N 3 2253.8 L-K-R-E-I-D-S-S-K-C-I-T-L-D-L-D-S-D-S-D

394 N 4 2269.7 K-R-E-I-D-S-S-K-C-I-T-L-D-L-D-S-D-S-D-E

395 N 5 2304.7 R-E-I-D-S-S-K-C-I-T-L-D-L-D-S-D-S-D-E-Y

396 N 6 2205.6 E-I-D-S-S-K-C-I-T-L-D-L-D-S-D-S-D-E-Y-G

397 N 7 2191.6 I-D-S-S-K-C-I-T-L-D-L-D-S-D-S-D-E-Y-G-D

398 N 8 2193.5 D-S-S-K-C-I-T-L-D-L-D-S-D-S-D-E-Y-G-D-D

399 N 9 2193.5 S-S-K-C-I-T-L-D-L-D-S-D-S-D-E-Y-G-D-D-D

400 N10 2237.6 S-K-C-I-T-L-D-L-D-S-D-S-D-E-Y-G-D-D-D-M

401 N11 2265.6 K-C-I-T-L-D-L-D-S-D-S-D-E-Y-G-D-D-D-M-D

402 N12 2224.5 C-I-T-L-D-L-D-S-D-S-D-E-Y-G-D-D-D-M-D-S

403 N13 2234.6 I-T-L-D-L-D-S-D-S-D-E-Y-G-D-D-D-M-D-S-I

404 N14 2249.6 T-L-D-L-D-S-D-S-D-E-Y-G-D-D-D-M-D-S-I-K

405 N15 2261.7 L-D-L-D-S-D-S-D-E-Y-G-D-D-D-M-D-S-I-K-L

406 N16 2235.6 D-L-D-S-D-S-D-E-Y-G-D-D-D-M-D-S-I-K-L-S

407 N17 2248.7 L-D-S-D-S-D-E-Y-G-D-D-D-M-D-S-I-K-L-S-K

408 N18 2250.6 D-S-D-S-D-E-Y-G-D-D-D-M-D-S-I-K-L-S-K-D

409 N19 2264.6 S-D-S-D-E-Y-G-D-D-D-M-D-S-I-K-L-S-K-D-E

410 N20 2264.6 D-S-D-E-Y-G-D-D-D-M-D-S-I-K-L-S-K-D-E-S

411 N21 2248.6 S-D-E-Y-G-D-D-D-M-D-S-I-K-L-S-K-D-E-S-V

412 N22 2274.7 D-E-Y-G-D-D-D-M-D-S-I-K-L-S-K-D-E-S-V-L

413 N23 2256.7 E-Y-G-D-D-D-M-D-S-I-K-L-S-K-D-E-S-V-L-P

414 N24 2240.8 Y-G-D-D-D-M-D-S-I-K-L-S-K-D-E-S-V-L-P-I

415 N25 2164.7 G-D-D-D-M-D-S-I-K-L-S-K-D-E-S-V-L-P-I-S

416 N26 2235.7 D-D-D-M-D-S-I-K-L-S-K-D-E-S-V-L-P-I-S-Q

417 N27 2233.8 D-D-M-D-S-I-K-L-S-K-D-E-S-V-L-P-I-S-Q-L

418 N28 2205.8 D-M-D-S-I-K-L-S-K-D-E-S-V-L-P-I-S-Q-L-S

419 N29 2218.9 M-D-S-I-K-L-S-K-D-E-S-V-L-P-I-S-Q-L-S-K

420 N30 2158.8 D-S-I-K-L-S-K-D-E-S-V-L-P-I-S-Q-L-S-K-A

421 O 1 2144.8 S-I-K-L-S-K-D-E-S-V-L-P-I-S-Q-L-S-K-A-T

422 O 2 2170.9 I-K-L-S-K-D-E-S-V-L-P-I-S-Q-L-S-K-A-T-I

423 O 3 2170.9 K-L-S-K-D-E-S-V-L-P-I-S-Q-L-S-K-A-T-I-L

424 O 4 2156.8 L-S-K-D-E-S-V-L-P-I-S-Q-L-S-K-A-T-I-L-N

425 O 5 2156.8 S-K-D-E-S-V-L-P-I-S-Q-L-S-K-A-T-I-L-N-L

426 O 6 2197.9 K-D-E-S-V-L-P-I-S-Q-L-S-K-A-T-I-L-N-L-K

427 O 7 2140.8 D-E-S-V-L-P-I-S-Q-L-S-K-A-T-I-L-N-L-K-A

428 O 8 2181.9 E-S-V-L-P-I-S-Q-L-S-K-A-T-I-L-N-L-K-A-R

429 O 9 2166 S-V-L-P-I-S-Q-L-S-K-A-T-I-L-N-L-K-A-R-L

430 O10 2166 V-L-P-I-S-Q-L-S-K-A-T-I-L-N-L-K-A-R-L-S

431 O11 2195.1 L-P-I-S-Q-L-S-K-A-T-I-L-N-L-K-A-R-L-S-K

432 O12 2210 P-I-S-Q-L-S-K-A-T-I-L-N-L-K-A-R-L-S-K-Q

433 O13 2227 I-S-Q-L-S-K-A-T-I-L-N-L-K-A-R-L-S-K-Q-N

434 O14 2241.9 S-Q-L-S-K-A-T-I-L-N-L-K-A-R-L-S-K-Q-N-Q

435 O15 2283 Q-L-S-K-A-T-I-L-N-L-K-A-R-L-S-K-Q-N-Q-K

436 O16 2268.1 L-S-K-A-T-I-L-N-L-K-A-R-L-S-K-Q-N-Q-K-L

437 O17 2242 S-K-A-T-I-L-N-L-K-A-R-L-S-K-Q-N-Q-K-L-S

438 O18 2283 K-A-T-I-L-N-L-K-A-R-L-S-K-Q-N-Q-K-L-S-Q

439 O19 2311 A-T-I-L-N-L-K-A-R-L-S-K-Q-N-Q-K-L-S-Q-R

440 O20 2337 T-I-L-N-L-K-A-R-L-S-K-Q-N-Q-K-L-S-Q-R-P

441 O21 2350 I-L-N-L-K-A-R-L-S-K-Q-N-Q-K-L-S-Q-R-P-N

442 O22 2365 L-N-L-K-A-R-L-S-K-Q-N-Q-K-L-S-Q-R-P-N-K

443 O23 2338.9 N-L-K-A-R-L-S-K-Q-N-Q-K-L-S-Q-R-P-N-K-S

444 O24 2353 L-K-A-R-L-S-K-Q-N-Q-K-L-S-Q-R-P-N-K-S-K

445 O25 2354.9 K-A-R-L-S-K-Q-N-Q-K-L-S-Q-R-P-N-K-S-K-D

446 O26 2323.8 A-R-L-S-K-Q-N-Q-K-L-S-Q-R-P-N-K-S-K-D-P

447 O27 2380.9 R-L-S-K-Q-N-Q-K-L-S-Q-R-P-N-K-S-K-D-P-K

448 O28 2323.8 L-S-K-Q-N-Q-K-L-S-Q-R-P-N-K-S-K-D-P-K-V

449 O29 2325.7 S-K-Q-N-Q-K-L-S-Q-R-P-N-K-S-K-D-P-K-V-D

450 O30 2375.7 K-Q-N-Q-K-L-S-Q-R-P-N-K-S-K-D-P-K-V-D-H

451 P 1 2361.6 Q-N-Q-K-L-S-Q-R-P-N-K-S-K-D-P-K-V-D-H-N

452 P 2 2332.6 N-Q-K-L-S-Q-R-P-N-K-S-K-D-P-K-V-D-H-N-V

453 P 3 2331.7 Q-K-L-S-Q-R-P-N-K-S-K-D-P-K-V-D-H-N-V-L

454 P 4 2316.8 K-L-S-Q-R-P-N-K-S-K-D-P-K-V-D-H-N-V-L-L

455 P 5 2302.7 L-S-Q-R-P-N-K-S-K-D-P-K-V-D-H-N-V-L-L-N

456 P 6 2290.6 S-Q-R-P-N-K-S-K-D-P-K-V-D-H-N-V-L-L-N-T

457 P 7 2316.7 Q-R-P-N-K-S-K-D-P-K-V-D-H-N-V-L-L-N-T-L

458 P 8 2344.8 R-P-N-K-S-K-D-P-K-V-D-H-N-V-L-L-N-T-L-R

459 P 9 2316.8 P-N-K-S-K-D-P-K-V-D-H-N-V-L-L-N-T-L-R-K

460 P10 2290.8 N-K-S-K-D-P-K-V-D-H-N-V-L-L-N-T-L-R-K-A

461 P11 2263.8 K-S-K-D-P-K-V-D-H-N-V-L-L-N-T-L-R-K-A-S

462 P12 2291.8 S-K-D-P-K-V-D-H-N-V-L-L-N-T-L-R-K-A-S-R

463 P13 2332.9 K-D-P-K-V-D-H-N-V-L-L-N-T-L-R-K-A-S-R-K

464 P14 2332.8 D-P-K-V-D-H-N-V-L-L-N-T-L-R-K-A-S-R-K-Q

465 P15 2330.9 P-K-V-D-H-N-V-L-L-N-T-L-R-K-A-S-R-K-Q-I

466 P16 2347 K-V-D-H-N-V-L-L-N-T-L-R-K-A-S-R-K-Q-I-L

467 P17 2333.9 V-D-H-N-V-L-L-N-T-L-R-K-A-S-R-K-Q-I-L-D

468 P18 2371.9 D-H-N-V-L-L-N-T-L-R-K-A-S-R-K-Q-I-L-D-H

469 P19 2384.9 H-N-V-L-L-N-T-L-R-K-A-S-R-K-Q-I-L-D-H-Q

470 P20 2376 N-V-L-L-N-T-L-R-K-A-S-R-K-Q-I-L-D-H-Q-K

471 P21 2391 V-L-L-N-T-L-R-K-A-S-R-K-Q-I-L-D-H-Q-K-E

472 P22 2391 L-L-N-T-L-R-K-A-S-R-K-Q-I-L-D-H-Q-K-E-V

473 P23 2391 L-N-T-L-R-K-A-S-R-K-Q-I-L-D-H-Q-K-E-V-I

474 P24 2406.9 N-T-L-R-K-A-S-R-K-Q-I-L-D-H-Q-K-E-V-I-E

475 P25 2393.9 T-L-R-K-A-S-R-K-Q-I-L-D-H-Q-K-E-V-I-E-T

476 P26 2421 L-R-K-A-S-R-K-Q-I-L-D-H-Q-K-E-V-I-E-T-K

477 P27 2364.9 R-K-A-S-R-K-Q-I-L-D-H-Q-K-E-V-I-E-T-K-G

478 P28 2321.9 K-A-S-R-K-Q-I-L-D-H-Q-K-E-V-I-E-T-K-G-L

479 P29 2321.9 A-S-R-K-Q-I-L-D-H-Q-K-E-V-I-E-T-K-G-L-K

480 P30 2364 S-R-K-Q-I-L-D-H-Q-K-E-V-I-E-T-K-G-L-K-L

481 Q 1 2406 R-K-Q-I-L-D-H-Q-K-E-V-I-E-T-K-G-L-K-L-E

482 Q 2 2364.9 K-Q-I-L-D-H-Q-K-E-V-I-E-T-K-G-L-K-L-E-D

483 Q 3 2367.9 Q-I-L-D-H-Q-K-E-V-I-E-T-K-G-L-K-L-E-D-M

484 Q 4 2310.9 I-L-D-H-Q-K-E-V-I-E-T-K-G-L-K-L-E-D-M-A

485 Q 5 2325.9 L-D-H-Q-K-E-V-I-E-T-K-G-L-K-L-E-D-M-A-K

486 Q 6 2341.8 D-H-Q-K-E-V-I-E-T-K-G-L-K-L-E-D-M-A-K-E

487 Q 7 2354.9 H-Q-K-E-V-I-E-T-K-G-L-K-L-E-D-M-A-K-E-K

488 Q 8 2346.9 Q-K-E-V-I-E-T-K-G-L-K-L-E-D-M-A-K-E-K-E

489 Q 9 2332 K-E-V-I-E-T-K-G-L-K-L-E-D-M-A-K-E-K-E-I

490 Q10 2302.9 E-V-I-E-T-K-G-L-K-L-E-D-M-A-K-E-K-E-I-V

491 Q11 2302.9 V-I-E-T-K-G-L-K-L-E-D-M-A-K-E-K-E-I-V-E

492 Q12 2317.9 I-E-T-K-G-L-K-L-E-D-M-A-K-E-K-E-I-V-E-N

493 Q13 2317.9 E-T-K-G-L-K-L-E-D-M-A-K-E-K-E-I-V-E-N-L

494 Q14 2302 T-K-G-L-K-L-E-D-M-A-K-E-K-E-I-V-E-N-L-L

495 Q15 2330 K-G-L-K-L-E-D-M-A-K-E-K-E-I-V-E-N-L-L-E

496 Q16 2329.9 G-L-K-L-E-D-M-A-K-E-K-E-I-V-E-N-L-L-E-Q

497 Q17 2401.9 L-K-L-E-D-M-A-K-E-K-E-I-V-E-N-L-L-E-Q-E

498 Q18 2401.9 K-L-E-D-M-A-K-E-K-E-I-V-E-N-L-L-E-Q-E-I

499 Q19 2386.9 L-E-D-M-A-K-E-K-E-I-V-E-N-L-L-E-Q-E-I-L

500 Q20 2429.9 E-D-M-A-K-E-K-E-I-V-E-N-L-L-E-Q-E-I-L-R

501 Q21 2414.9 D-M-A-K-E-K-E-I-V-E-N-L-L-E-Q-E-I-L-R-N

502 Q22 2428 M-A-K-E-K-E-I-V-E-N-L-L-E-Q-E-I-L-R-N-K

503 Q23 2453 A-K-E-K-E-I-V-E-N-L-L-E-Q-E-I-L-R-N-K-R

504 Q24 2495.1 K-E-K-E-I-V-E-N-L-L-E-Q-E-I-L-R-N-K-R-I

505 Q25 2523.1 E-K-E-I-V-E-N-L-L-E-Q-E-I-L-R-N-K-R-I-R

506 Q26 2522.1 K-E-I-V-E-N-L-L-E-Q-E-I-L-R-N-K-R-I-R-Q

507 Q27 2522.1 E-I-V-E-N-L-L-E-Q-E-I-L-R-N-K-R-I-R-Q-K

508 Q28 2522.1 I-V-E-N-L-L-E-Q-E-I-L-R-N-K-R-I-R-Q-K-E

509 Q29 2537.1 V-E-N-L-L-E-Q-E-I-L-R-N-K-R-I-R-Q-K-E-K

510 Q30 2594.2 E-N-L-L-E-Q-E-I-L-R-N-K-R-I-R-Q-K-E-K-R

511 R 1 2621.3 N-L-L-E-Q-E-I-L-R-N-K-R-I-R-Q-K-E-K-R-R

512 R 2 2636.3 L-L-E-Q-E-I-L-R-N-K-R-I-R-Q-K-E-K-R-R-E

513 R 3 2651.3 L-E-Q-E-I-L-R-N-K-R-I-R-Q-K-E-K-R-R-E-K

514 R 4 2651.3 E-Q-E-I-L-R-N-K-R-I-R-Q-K-E-K-R-R-E-K-L

515 R 5 2651.3 Q-E-I-L-R-N-K-R-I-R-Q-K-E-K-R-R-E-K-L-E

516 R 6 2652.3 E-I-L-R-N-K-R-I-R-Q-K-E-K-R-R-E-K-L-E-E

517 R 7 2637.3 I-L-R-N-K-R-I-R-Q-K-E-K-R-R-E-K-L-E-E-N

518 R 8 2639.2 L-R-N-K-R-I-R-Q-K-E-K-R-R-E-K-L-E-E-N-D

519 R 9 2673.2 R-N-K-R-I-R-Q-K-E-K-R-R-E-K-L-E-E-N-D-F

520 R10 2645.1 N-K-R-I-R-Q-K-E-K-R-R-E-K-L-E-E-N-D-F-Q

521 R11 2644.2 K-R-I-R-Q-K-E-K-R-R-E-K-L-E-E-N-D-F-Q-L

522 R12 2630.1 R-I-R-Q-K-E-K-R-R-E-K-L-E-E-N-D-F-Q-L-N

523 R13 2545 I-R-Q-K-E-K-R-R-E-K-L-E-E-N-D-F-Q-L-N-A

524 R14 2568.9 R-Q-K-E-K-R-R-E-K-L-E-E-N-D-F-Q-L-N-A-H

525 R15 2527.8 Q-K-E-K-R-R-E-K-L-E-E-N-D-F-Q-L-N-A-H-D

526 R16 2486.8 K-E-K-R-R-E-K-L-E-E-N-D-F-Q-L-N-A-H-D-S

527 R17 2415.7 E-K-R-R-E-K-L-E-E-N-D-F-Q-L-N-A-H-D-S-G

528 R18 2373.7 K-R-R-E-K-L-E-E-N-D-F-Q-L-N-A-H-D-S-G-S

529 R19 2360.6 R-R-E-K-L-E-E-N-D-F-Q-L-N-A-H-D-S-G-S-D

530 R20 2291.5 R-E-K-L-E-E-N-D-F-Q-L-N-A-H-D-S-G-S-D-S

531 R21 2192.4 E-K-L-E-E-N-D-F-Q-L-N-A-H-D-S-G-S-D-S-G

532 R22 2150.4 K-L-E-E-N-D-F-Q-L-N-A-H-D-S-G-S-D-S-G-S

533 R23 2151.3 L-E-E-N-D-F-Q-L-N-A-H-D-S-G-S-D-S-G-S-E

534 R24 2125.2 E-E-N-D-F-Q-L-N-A-H-D-S-G-S-D-S-G-S-E-S

535 R25 2083.2 E-N-D-F-Q-L-N-A-H-D-S-G-S-D-S-G-S-E-S-S

536 R26 2011.2 N-D-F-Q-L-N-A-H-D-S-G-S-D-S-G-S-E-S-S-G

537 R27 2044.3 D-F-Q-L-N-A-H-D-S-G-S-D-S-G-S-E-S-S-G-F

538 R28 2000.3 F-Q-L-N-A-H-D-S-G-S-D-S-G-S-E-S-S-G-F-A

539 R29 1966.3 Q-L-N-A-H-D-S-G-S-D-S-G-S-E-S-S-G-F-A-L

540 R30 1925.3 L-N-A-H-D-S-G-S-D-S-G-S-E-S-S-G-F-A-L-S

541 S 1 1869.2 N-A-H-D-S-G-S-D-S-G-S-E-S-S-G-F-A-L-S-G

542 S 2 1869.2 A-H-D-S-G-S-D-S-G-S-E-S-S-G-F-A-L-S-G-N

543 S 3 1927.2 H-D-S-G-S-D-S-G-S-E-S-S-G-F-A-L-S-G-N-E

544 S 4 1903.3 D-S-G-S-D-S-G-S-E-S-S-G-F-A-L-S-G-N-E-I

545 S 5 1859.3 S-G-S-D-S-G-S-E-S-S-G-F-A-L-S-G-N-E-I-A

546 S 6 1887.3 G-S-D-S-G-S-E-S-S-G-F-A-L-S-G-N-E-I-A-D

547 S 7 1993.4 S-D-S-G-S-E-S-S-G-F-A-L-S-G-N-E-I-A-D-Y

548 S 8 2035.4 D-S-G-S-E-S-S-G-F-A-L-S-G-N-E-I-A-D-Y-E

549 S 9 2007.4 S-G-S-E-S-S-G-F-A-L-S-G-N-E-I-A-D-Y-E-S

550 S10 2007.4 G-S-E-S-S-G-F-A-L-S-G-N-E-I-A-D-Y-E-S-S

551 S11 2007.4 S-E-S-S-G-F-A-L-S-G-N-E-I-A-D-Y-E-S-S-G

552 S12 2007.4 E-S-S-G-F-A-L-S-G-N-E-I-A-D-Y-E-S-S-G-S

553 S13 2007.4 S-S-G-F-A-L-S-G-N-E-I-A-D-Y-E-S-S-G-S-E

554 S14 2034.4 S-G-F-A-L-S-G-N-E-I-A-D-Y-E-S-S-G-S-E-N

555 S15 2062.4 G-F-A-L-S-G-N-E-I-A-D-Y-E-S-S-G-S-E-N-D

556 S16 2119.4 F-A-L-S-G-N-E-I-A-D-Y-E-S-S-G-S-E-N-D-N

557 S17 2128.4 A-L-S-G-N-E-I-A-D-Y-E-S-S-G-S-E-N-D-N-R

558 S18 2213.5 L-S-G-N-E-I-A-D-Y-E-S-S-G-S-E-N-D-N-R-R

559 S19 2229.4 S-G-N-E-I-A-D-Y-E-S-S-G-S-E-N-D-N-R-R-E

560 S20 2229.4 G-N-E-I-A-D-Y-E-S-S-G-S-E-N-D-N-R-R-E-S

561 S21 2287.4 N-E-I-A-D-Y-E-S-S-G-S-E-N-D-N-R-R-E-S-D

562 S22 2260.4 E-I-A-D-Y-E-S-S-G-S-E-N-D-N-R-R-E-S-D-S

563 S23 2260.4 I-A-D-Y-E-S-S-G-S-E-N-D-N-R-R-E-S-D-S-E

564 S24 2275.4 A-D-Y-E-S-S-G-S-E-N-D-N-R-R-E-S-D-S-E-K

565 S25 2333.4 D-Y-E-S-S-G-S-E-N-D-N-R-R-E-S-D-S-E-K-E

566 S26 2333.4 Y-E-S-S-G-S-E-N-D-N-R-R-E-S-D-S-E-K-E-D

567 S27 2285.3 E-S-S-G-S-E-N-D-N-R-R-E-S-D-S-E-K-E-D-D

568 S28 2285.3 S-S-G-S-E-N-D-N-R-R-E-S-D-S-E-K-E-D-D-E

569 S29 2311.4 S-G-S-E-N-D-N-R-R-E-S-D-S-E-K-E-D-D-E-I

570 S30 2337.5 G-S-E-N-D-N-R-R-E-S-D-S-E-K-E-D-D-E-I-I

571 T 1 2393.6 S-E-N-D-N-R-R-E-S-D-S-E-K-E-D-D-E-I-I-L

572 T 2 2434.7 E-N-D-N-R-R-E-S-D-S-E-K-E-D-D-E-I-I-L-K

573 T 3 2433.7 N-D-N-R-R-E-S-D-S-E-K-E-D-D-E-I-I-L-K-Q

574 T 4 2447.8 D-N-R-R-E-S-D-S-E-K-E-D-D-E-I-I-L-K-Q-K

575 T 5 2460.9 N-R-R-E-S-D-S-E-K-E-D-D-E-I-I-L-K-Q-K-K

576 T 6 2433.9 R-R-E-S-D-S-E-K-E-D-D-E-I-I-L-K-Q-K-K-S

577 T 7 2414.8 R-E-S-D-S-E-K-E-D-D-E-I-I-L-K-Q-K-K-S-H

578 T 8 2395.7 E-S-D-S-E-K-E-D-D-E-I-I-L-K-Q-K-K-S-H-H

579 T 9 2365.7 S-D-S-E-K-E-D-D-E-I-I-L-K-Q-K-K-S-H-H-V

580 T10 2406.8 D-S-E-K-E-D-D-E-I-I-L-K-Q-K-K-S-H-H-V-K

581 T11 2428.8 S-E-K-E-D-D-E-I-I-L-K-Q-K-K-S-H-H-V-K-H

582 T12 2454.9 E-K-E-D-D-E-I-I-L-K-Q-K-K-S-H-H-V-K-H-I

583 T13 2439 K-E-D-D-E-I-I-L-K-Q-K-K-S-H-H-V-K-H-I-I

584 T14 2424.9 E-D-D-E-I-I-L-K-Q-K-K-S-H-H-V-K-H-I-I-N

585 T15 2424.9 D-D-E-I-I-L-K-Q-K-K-S-H-H-V-K-H-I-I-N-E

586 T16 2396.9 D-E-I-I-L-K-Q-K-K-S-H-H-V-K-H-I-I-N-E-S

587 T17 2396.9 E-I-I-L-K-Q-K-K-S-H-H-V-K-H-I-I-N-E-S-D

588 T18 2354.9 I-I-L-K-Q-K-K-S-H-H-V-K-H-I-I-N-E-S-D-S

589 T19 2356.8 I-L-K-Q-K-K-S-H-H-V-K-H-I-I-N-E-S-D-S-D

590 T20 2344.7 L-K-Q-K-K-S-H-H-V-K-H-I-I-N-E-S-D-S-D-T

591 T21 2360.6 K-Q-K-K-S-H-H-V-K-H-I-I-N-E-S-D-S-D-T-E

592 T22 2331.5 Q-K-K-S-H-H-V-K-H-I-I-N-E-S-D-S-D-T-E-V

593 T23 2332.5 K-K-S-H-H-V-K-H-I-I-N-E-S-D-S-D-T-E-V-E

594 T24 2275.4 K-S-H-H-V-K-H-I-I-N-E-S-D-S-D-T-E-V-E-A

595 T25 2275.4 S-H-H-V-K-H-I-I-N-E-S-D-S-D-T-E-V-E-A-K

596 T26 2285.4 H-H-V-K-H-I-I-N-E-S-D-S-D-T-E-V-E-A-K-P

597 T27 2276.5 H-V-K-H-I-I-N-E-S-D-S-D-T-E-V-E-A-K-P-K

598 T28 2268.5 V-K-H-I-I-N-E-S-D-S-D-T-E-V-E-A-K-P-K-E

599 T29 2297.6 K-H-I-I-N-E-S-D-S-D-T-E-V-E-A-K-P-K-E-K

600 T30 2240.5 H-I-I-N-E-S-D-S-D-T-E-V-E-A-K-P-K-E-K-A

601 A 1 2218.5 I-I-N-E-S-D-S-D-T-E-V-E-A-K-P-K-E-K-A-D

602 A 2 2234.4 I-N-E-S-D-S-D-T-E-V-E-A-K-P-K-E-K-A-D-E

603 A 3 2208.3 N-E-S-D-S-D-T-E-V-E-A-K-P-K-E-K-A-D-E-S

604 A 4 2207.4 E-S-D-S-D-T-E-V-E-A-K-P-K-E-K-A-D-E-S-L

605 A 5 2175.4 S-D-S-D-T-E-V-E-A-K-P-K-E-K-A-D-E-S-L-P

606 A 6 2216.5 D-S-D-T-E-V-E-A-K-P-K-E-K-A-D-E-S-L-P-K

607 A 7 2257.6 S-D-T-E-V-E-A-K-P-K-E-K-A-D-E-S-L-P-K-R

608 A 8 2283.7 D-T-E-V-E-A-K-P-K-E-K-A-D-E-S-L-P-K-R-I

609 A 9 2239.7 T-E-V-E-A-K-P-K-E-K-A-D-E-S-L-P-K-R-I-A

610 A10 2251.8 E-V-E-A-K-P-K-E-K-A-D-E-S-L-P-K-R-I-A-I

611 A11 2236.8 V-E-A-K-P-K-E-K-A-D-E-S-L-P-K-R-I-A-I-N

612 A12 2250.9 E-A-K-P-K-E-K-A-D-E-S-L-P-K-R-I-A-I-N-L

613 A13 2178.9 A-K-P-K-E-K-A-D-E-S-L-P-K-R-I-A-I-N-L-G

614 A14 2244.9 K-P-K-E-K-A-D-E-S-L-P-K-R-I-A-I-N-L-G-H

615 A15 2279.9 P-K-E-K-A-D-E-S-L-P-K-R-I-A-I-N-L-G-H-Y

616 A16 2239.9 K-E-K-A-D-E-S-L-P-K-R-I-A-I-N-L-G-H-Y-G

617 A17 2226.8 E-K-A-D-E-S-L-P-K-R-I-A-I-N-L-G-H-Y-G-D

618 A18 2211.8 K-A-D-E-S-L-P-K-R-I-A-I-N-L-G-H-Y-G-D-N

619 A19 2196.8 A-D-E-S-L-P-K-R-I-A-I-N-L-G-H-Y-G-D-N-I

620 A20 2182.8 D-E-S-L-P-K-R-I-A-I-N-L-G-H-Y-G-D-N-I-G

621 A21 2196.8 E-S-L-P-K-R-I-A-I-N-L-G-H-Y-G-D-N-I-G-E

622 A22 2182.8 S-L-P-K-R-I-A-I-N-L-G-H-Y-G-D-N-I-G-E-D

623 A23 2196.8 L-P-K-R-I-A-I-N-L-G-H-Y-G-D-N-I-G-E-D-T

624 A24 2198.7 P-K-R-I-A-I-N-L-G-H-Y-G-D-N-I-G-E-D-T-D

625 A25 2229.8 K-R-I-A-I-N-L-G-H-Y-G-D-N-I-G-E-D-T-D-K

626 A26 2248.8 R-I-A-I-N-L-G-H-Y-G-D-N-I-G-E-D-T-D-K-F

627 A27 2220.7 I-A-I-N-L-G-H-Y-G-D-N-I-G-E-D-T-D-K-F-Q

628 A28 2236.6 A-I-N-L-G-H-Y-G-D-N-I-G-E-D-T-D-K-F-Q-E

629 A29 2266.6 I-N-L-G-H-Y-G-D-N-I-G-E-D-T-D-K-F-Q-E-T

630 A30 2267.5 N-L-G-H-Y-G-D-N-I-G-E-D-T-D-K-F-Q-E-T-N

631 B 1 2252.5 L-G-H-Y-G-D-N-I-G-E-D-T-D-K-F-Q-E-T-N-V

632 B 2 2252.5 G-H-Y-G-D-N-I-G-E-D-T-D-K-F-Q-E-T-N-V-L

633 B 3 2310.5 H-Y-G-D-N-I-G-E-D-T-D-K-F-Q-E-T-N-V-L-D

634 B 4 2274.5 Y-G-D-N-I-G-E-D-T-D-K-F-Q-E-T-N-V-L-D-T

635 B 5 2239.4 G-D-N-I-G-E-D-T-D-K-F-Q-E-T-N-V-L-D-T-Q

636 B 6 2296.4 D-N-I-G-E-D-T-D-K-F-Q-E-T-N-V-L-D-T-Q-N

637 B 7 2294.5 N-I-G-E-D-T-D-K-F-Q-E-T-N-V-L-D-T-Q-N-I

638 B 8 2309.5 I-G-E-D-T-D-K-F-Q-E-T-N-V-L-D-T-Q-N-I-E

639 B 9 2325.4 G-E-D-T-D-K-F-Q-E-T-N-V-L-D-T-Q-N-I-E-E

640 B10 2367.4 E-D-T-D-K-F-Q-E-T-N-V-L-D-T-Q-N-I-E-E-V

641 B11 2369.5 D-T-D-K-F-Q-E-T-N-V-L-D-T-Q-N-I-E-E-V-M

642 B12 2325.5 T-D-K-F-Q-E-T-N-V-L-D-T-Q-N-I-E-E-V-M-A

643 B13 2353.5 D-K-F-Q-E-T-N-V-L-D-T-Q-N-I-E-E-V-M-A-E

644 B14 2394.6 K-F-Q-E-T-N-V-L-D-T-Q-N-I-E-E-V-M-A-E-R

645 B15 2380.5 F-Q-E-T-N-V-L-D-T-Q-N-I-E-E-V-M-A-E-R-N

646 B16 2334.4 Q-E-T-N-V-L-D-T-Q-N-I-E-E-V-M-A-E-R-N-T

647 B17 2319.5 E-T-N-V-L-D-T-Q-N-I-E-E-V-M-A-E-R-N-T-I

648 B18 2319.5 T-N-V-L-D-T-Q-N-I-E-E-V-M-A-E-R-N-T-I-E

649 B19 2332.5 N-V-L-D-T-Q-N-I-E-E-V-M-A-E-R-N-T-I-E-N

650 B20 2347.5 V-L-D-T-Q-N-I-E-E-V-M-A-E-R-N-T-I-E-N-E

651 B21 2347.5 L-D-T-Q-N-I-E-E-V-M-A-E-R-N-T-I-E-N-E-V

652 B22 2362.5 D-T-Q-N-I-E-E-V-M-A-E-R-N-T-I-E-N-E-V-K

653 B23 2362.5 T-Q-N-I-E-E-V-M-A-E-R-N-T-I-E-N-E-V-K-D

654 B24 2376.5 Q-N-I-E-E-V-M-A-E-R-N-T-I-E-N-E-V-K-D-D

655 B25 2347.5 N-I-E-E-V-M-A-E-R-N-T-I-E-N-E-V-K-D-D-V

656 B26 2396.6 I-E-E-V-M-A-E-R-N-T-I-E-N-E-V-K-D-D-V-Y

657 B27 2382.5 E-E-V-M-A-E-R-N-T-I-E-N-E-V-K-D-D-V-Y-V

658 B28 2367.5 E-V-M-A-E-R-N-T-I-E-N-E-V-K-D-D-V-Y-V-N

659 B29 2367.5 V-M-A-E-R-N-T-I-E-N-E-V-K-D-D-V-Y-V-N-E

660 B30 2397.5 M-A-E-R-N-T-I-E-N-E-V-K-D-D-V-Y-V-N-E-E

661 C 1 2337.4 A-E-R-N-T-I-E-N-E-V-K-D-D-V-Y-V-N-E-E-A

662 C 2 2381.4 E-R-N-T-I-E-N-E-V-K-D-D-V-Y-V-N-E-E-A-D

663 C 3 2381.4 R-N-T-I-E-N-E-V-K-D-D-V-Y-V-N-E-E-A-D-E

664 C 4 2296.3 N-T-I-E-N-E-V-K-D-D-V-Y-V-N-E-E-A-D-E-A

665 C 5 2295.4 T-I-E-N-E-V-K-D-D-V-Y-V-N-E-E-A-D-E-A-I

666 C 6 2350.5 I-E-N-E-V-K-D-D-V-Y-V-N-E-E-A-D-E-A-I-R

667 C 7 2393.5 E-N-E-V-K-D-D-V-Y-V-N-E-E-A-D-E-A-I-R-R

668 C 8 2392.5 N-E-V-K-D-D-V-Y-V-N-E-E-A-D-E-A-I-R-R-Q

669 C 9 2391.6 E-V-K-D-D-V-Y-V-N-E-E-A-D-E-A-I-R-R-Q-L

670 C10 2375.7 V-K-D-D-V-Y-V-N-E-E-A-D-E-A-I-R-R-Q-L-I

671 C11 2391.7 K-D-D-V-Y-V-N-E-E-A-D-E-A-I-R-R-Q-L-I-D

672 C12 2391.7 D-D-V-Y-V-N-E-E-A-D-E-A-I-R-R-Q-L-I-D-K

673 C13 2405.7 D-V-Y-V-N-E-E-A-D-E-A-I-R-R-Q-L-I-D-K-E

674 C14 2418.8 V-Y-V-N-E-E-A-D-E-A-I-R-R-Q-L-I-D-K-E-K

675 C15 2432.9 Y-V-N-E-E-A-D-E-A-I-R-R-Q-L-I-D-K-E-K-L

676 C16 2397.8 V-N-E-E-A-D-E-A-I-R-R-Q-L-I-D-K-E-K-L-Q

677 C17 2411.9 N-E-E-A-D-E-A-I-R-R-Q-L-I-D-K-E-K-L-Q-L

678 C18 2426 E-E-A-D-E-A-I-R-R-Q-L-I-D-K-E-K-L-Q-L-K

679 C19 2425 E-A-D-E-A-I-R-R-Q-L-I-D-K-E-K-L-Q-L-K-Q

680 C20 2424.1 A-D-E-A-I-R-R-Q-L-I-D-K-E-K-L-Q-L-K-Q-K

681 C21 2482.1 D-E-A-I-R-R-Q-L-I-D-K-E-K-L-Q-L-K-Q-K-E

682 C22 2495.2 E-A-I-R-R-Q-L-I-D-K-E-K-L-Q-L-K-Q-K-E-K

683 C23 2495.2 A-I-R-R-Q-L-I-D-K-E-K-L-Q-L-K-Q-K-E-K-E

684 C24 2561.2 I-R-R-Q-L-I-D-K-E-K-L-Q-L-K-Q-K-E-K-E-H

685 C25 2577.1 R-R-Q-L-I-D-K-E-K-L-Q-L-K-Q-K-E-K-E-H-E

686 C26 2492 R-Q-L-I-D-K-E-K-L-Q-L-K-Q-K-E-K-E-H-E-A

687 C27 2464 Q-L-I-D-K-E-K-L-Q-L-K-Q-K-E-K-E-H-E-A-K

688 C28 2449.1 L-I-D-K-E-K-L-Q-L-K-Q-K-E-K-E-H-E-A-K-I

689 C29 2464.1 I-D-K-E-K-L-Q-L-K-Q-K-E-K-E-H-E-A-K-I-K

690 C30 2480 D-K-E-K-L-Q-L-K-Q-K-E-K-E-H-E-A-K-I-K-E

691 D 1 2478.1 K-E-K-L-Q-L-K-Q-K-E-K-E-H-E-A-K-I-K-E-L

692 D 2 2478.1 E-K-L-Q-L-K-Q-K-E-K-E-H-E-A-K-I-K-E-L-K

693 D 3 2477.2 K-L-Q-L-K-Q-K-E-K-E-H-E-A-K-I-K-E-L-K-K

694 D 4 2505.2 L-Q-L-K-Q-K-E-K-E-H-E-A-K-I-K-E-L-K-K-R

695 D 5 2449.1 Q-L-K-Q-K-E-K-E-H-E-A-K-I-K-E-L-K-K-R-G

696 D 6 2420.1 L-K-Q-K-E-K-E-H-E-A-K-I-K-E-L-K-K-R-G-V

697 D 7 2408 K-Q-K-E-K-E-H-E-A-K-I-K-E-L-K-K-R-G-V-T

698 D 8 2393.9 Q-K-E-K-E-H-E-A-K-I-K-E-L-K-K-R-G-V-T-N

699 D 9 2413 K-E-K-E-H-E-A-K-I-K-E-L-K-K-R-G-V-T-N-F

700 D10 2432 E-K-E-H-E-A-K-I-K-E-L-K-K-R-G-V-T-N-F-F

701 D11 2432 K-E-H-E-A-K-I-K-E-L-K-K-R-G-V-T-N-F-F-E

702 D12 2435 E-H-E-A-K-I-K-E-L-K-K-R-G-V-T-N-F-F-E-M

703 D13 2435 H-E-A-K-I-K-E-L-K-K-R-G-V-T-N-F-F-E-M-E

704 D14 2369 E-A-K-I-K-E-L-K-K-R-G-V-T-N-F-F-E-M-E-A

705 D15 2369 A-K-I-K-E-L-K-K-R-G-V-T-N-F-F-E-M-E-A-E

706 D16 2427 K-I-K-E-L-K-K-R-G-V-T-N-F-F-E-M-E-A-E-E

707 D17 2385.9 I-K-E-L-K-K-R-G-V-T-N-F-F-E-M-E-A-E-E-S

708 D18 2401.8 K-E-L-K-K-R-G-V-T-N-F-F-E-M-E-A-E-E-S-E

709 D19 2388.7 E-L-K-K-R-G-V-T-N-F-F-E-M-E-A-E-E-S-E-D

710 D20 2388.7 L-K-K-R-G-V-T-N-F-F-E-M-E-A-E-E-S-E-D-E

711 D21 2461.7 K-K-R-G-V-T-N-F-F-E-M-E-A-E-E-S-E-D-E-W

712 D22 2470.6 K-R-G-V-T-N-F-F-E-M-E-A-E-E-S-E-D-E-W-H

713 D23 2399.5 R-G-V-T-N-F-F-E-M-E-A-E-E-S-E-D-E-W-H-G

714 D24 2356.5 G-V-T-N-F-F-E-M-E-A-E-E-S-E-D-E-W-H-G-I

715 D25 2356.5 V-T-N-F-F-E-M-E-A-E-E-S-E-D-E-W-H-G-I-G

716 D26 2314.5 T-N-F-F-E-M-E-A-E-E-S-E-D-E-W-H-G-I-G-G

717 D27 2284.5 N-F-F-E-M-E-A-E-E-S-E-D-E-W-H-G-I-G-G-A

718 D28 2285.5 F-F-E-M-E-A-E-E-S-E-D-E-W-H-G-I-G-G-A-D

719 D29 2195.4 F-E-M-E-A-E-E-S-E-D-E-W-H-G-I-G-G-A-D-G

720 D30 2177.3 E-M-E-A-E-E-S-E-D-E-W-H-G-I-G-G-A-D-G-E

721 E 1 2105.3 M-E-A-E-E-S-E-D-E-W-H-G-I-G-G-A-D-G-E-G

722 E 2 2061.2 E-A-E-E-S-E-D-E-W-H-G-I-G-G-A-D-G-E-G-S

723 E 3 2047.2 A-E-E-S-E-D-E-W-H-G-I-G-G-A-D-G-E-G-S-D

724 E 4 2091.2 E-E-S-E-D-E-W-H-G-I-G-G-A-D-G-E-G-S-D-D

725 E 5 2125.3 E-S-E-D-E-W-H-G-I-G-G-A-D-G-E-G-S-D-D-Y

726 E 6 2111.3 S-E-D-E-W-H-G-I-G-G-A-D-G-E-G-S-D-D-Y-D

727 E 7 2111.3 E-D-E-W-H-G-I-G-G-A-D-G-E-G-S-D-D-Y-D-S

728 E 8 2097.3 D-E-W-H-G-I-G-G-A-D-G-E-G-S-D-D-Y-D-S-D

729 E 9 2095.4 E-W-H-G-I-G-G-A-D-G-E-G-S-D-D-Y-D-S-D-L

730 E10 2095.4 W-H-G-I-G-G-A-D-G-E-G-S-D-D-Y-D-S-D-L-E

731 E11 2037.4 H-G-I-G-G-A-D-G-E-G-S-D-D-Y-D-S-D-L-E-K

732 E12 2031.5 G-I-G-G-A-D-G-E-G-S-D-D-Y-D-S-D-L-E-K-M

733 E13 2087.6 I-G-G-A-D-G-E-G-S-D-D-Y-D-S-D-L-E-K-M-I

734 E14 2089.5 G-G-A-D-G-E-G-S-D-D-Y-D-S-D-L-E-K-M-I-D

735 E15 2147.5 G-A-D-G-E-G-S-D-D-Y-D-S-D-L-E-K-M-I-D-D

736 E16 2253.6 A-D-G-E-G-S-D-D-Y-D-S-D-L-E-K-M-I-D-D-Y

737 E17 2269.6 D-G-E-G-S-D-D-Y-D-S-D-L-E-K-M-I-D-D-Y-S

738 E18 2282.7 G-E-G-S-D-D-Y-D-S-D-L-E-K-M-I-D-D-Y-S-K

739 E19 2339.7 E-G-S-D-D-Y-D-S-D-L-E-K-M-I-D-D-Y-S-K-N

740 E20 2324.7 G-S-D-D-Y-D-S-D-L-E-K-M-I-D-D-Y-S-K-N-N

741 E21 2414.8 S-D-D-Y-D-S-D-L-E-K-M-I-D-D-Y-S-K-N-N-F

742 E22 2441.8 D-D-Y-D-S-D-L-E-K-M-I-D-D-Y-S-K-N-N-F-N

743 E23 2423.8 D-Y-D-S-D-L-E-K-M-I-D-D-Y-S-K-N-N-F-N-P

744 E24 2445.8 Y-D-S-D-L-E-K-M-I-D-D-Y-S-K-N-N-F-N-P-H

745 E25 2411.7 D-S-D-L-E-K-M-I-D-D-Y-S-K-N-N-F-N-P-H-E

746 E26 2409.8 S-D-L-E-K-M-I-D-D-Y-S-K-N-N-F-N-P-H-E-I

747 E27 2478.9 D-L-E-K-M-I-D-D-Y-S-K-N-N-F-N-P-H-E-I-R

748 E28 2492.9 L-E-K-M-I-D-D-Y-S-K-N-N-F-N-P-H-E-I-R-E

749 E29 2510.9 E-K-M-I-D-D-Y-S-K-N-N-F-N-P-H-E-I-R-E-M

750 E30 2495 K-M-I-D-D-Y-S-K-N-N-F-N-P-H-E-I-R-E-M-L

751 F 1 2437.9 M-I-D-D-Y-S-K-N-N-F-N-P-H-E-I-R-E-M-L-A

752 F 2 2377.8 I-D-D-Y-S-K-N-N-F-N-P-H-E-I-R-E-M-L-A-A

753 F 3 2393.7 D-D-Y-S-K-N-N-F-N-P-H-E-I-R-E-M-L-A-A-E

754 F 4 2392.7 D-Y-S-K-N-N-F-N-P-H-E-I-R-E-M-L-A-A-E-N

755 F 5 2405.8 Y-S-K-N-N-F-N-P-H-E-I-R-E-M-L-A-A-E-N-K

756 F 6 2371.7 S-K-N-N-F-N-P-H-E-I-R-E-M-L-A-A-E-N-K-E

757 F 7 2415.8 K-N-N-F-N-P-H-E-I-R-E-M-L-A-A-E-N-K-E-M

758 F 8 2402.7 N-N-F-N-P-H-E-I-R-E-M-L-A-A-E-N-K-E-M-D

759 F 9 2401.8 N-F-N-P-H-E-I-R-E-M-L-A-A-E-N-K-E-M-D-I

760 F10 2415.9 F-N-P-H-E-I-R-E-M-L-A-A-E-N-K-E-M-D-I-K

761 F11 2399.9 N-P-H-E-I-R-E-M-L-A-A-E-N-K-E-M-D-I-K-M

762 F12 2399 P-H-E-I-R-E-M-L-A-A-E-N-K-E-M-D-I-K-M-I

763 F13 2416 H-E-I-R-E-M-L-A-A-E-N-K-E-M-D-I-K-M-I-N

764 F14 2407.1 E-I-R-E-M-L-A-A-E-N-K-E-M-D-I-K-M-I-N-K

765 F15 2391.2 I-R-E-M-L-A-A-E-N-K-E-M-D-I-K-M-I-N-K-I

766 F16 2391.2 R-E-M-L-A-A-E-N-K-E-M-D-I-K-M-I-N-K-I-L

767 F17 2398.2 E-M-L-A-A-E-N-K-E-M-D-I-K-M-I-N-K-I-L-Y

768 F18 2384.2 M-L-A-A-E-N-K-E-M-D-I-K-M-I-N-K-I-L-Y-D

769 F19 2366.2 L-A-A-E-N-K-E-M-D-I-K-M-I-N-K-I-L-Y-D-I

770 F20 2381.2 A-A-E-N-K-E-M-D-I-K-M-I-N-K-I-L-Y-D-I-K

771 F21 2424.2 A-E-N-K-E-M-D-I-K-M-I-N-K-I-L-Y-D-I-K-N

772 F22 2410.2 E-N-K-E-M-D-I-K-M-I-N-K-I-L-Y-D-I-K-N-G

773 F23 2338.2 N-K-E-M-D-I-K-M-I-N-K-I-L-Y-D-I-K-N-G-G

774 F24 2371.3 K-E-M-D-I-K-M-I-N-K-I-L-Y-D-I-K-N-G-G-F

775 F25 2399.3 E-M-D-I-K-M-I-N-K-I-L-Y-D-I-K-N-G-G-F-R

776 F26 2384.3 M-D-I-K-M-I-N-K-I-L-Y-D-I-K-N-G-G-F-R-N

777 F27 2381.3 D-I-K-M-I-N-K-I-L-Y-D-I-K-N-G-G-F-R-N-K

778 F28 2422.4 I-K-M-I-N-K-I-L-Y-D-I-K-N-G-G-F-R-N-K-R

779 F29 2380.3 K-M-I-N-K-I-L-Y-D-I-K-N-G-G-F-R-N-K-R-A

780 F30 2380.3 M-I-N-K-I-L-Y-D-I-K-N-G-G-F-R-N-K-R-A-K

781 G 1 2363.2 I-N-K-I-L-Y-D-I-K-N-G-G-F-R-N-K-R-A-K-N

782 G 2 2337.1 N-K-I-L-Y-D-I-K-N-G-G-F-R-N-K-R-A-K-N-S

783 G 3 2336.2 K-I-L-Y-D-I-K-N-G-G-F-R-N-K-R-A-K-N-S-L

784 G 4 2337.1 I-L-Y-D-I-K-N-G-G-F-R-N-K-R-A-K-N-S-L-E

785 G 5 2337.1 L-Y-D-I-K-N-G-G-F-R-N-K-R-A-K-N-S-L-E-L

786 G 6 2353 Y-D-I-K-N-G-G-F-R-N-K-R-A-K-N-S-L-E-L-E

787 G 7 2303 D-I-K-N-G-G-F-R-N-K-R-A-K-N-S-L-E-L-E-L

788 G 8 2275 I-K-N-G-G-F-R-N-K-R-A-K-N-S-L-E-L-E-L-S

789 G 9 2276.9 K-N-G-G-F-R-N-K-R-A-K-N-S-L-E-L-E-L-S-D

790 G10 2263.8 N-G-G-F-R-N-K-R-A-K-N-S-L-E-L-E-L-S-D-D

791 G11 2264.8 G-G-F-R-N-K-R-A-K-N-S-L-E-L-E-L-S-D-D-D

792 G12 2336.8 G-F-R-N-K-R-A-K-N-S-L-E-L-E-L-S-D-D-D-E

793 G13 2394.8 F-R-N-K-R-A-K-N-S-L-E-L-E-L-S-D-D-D-E-D

794 G14 2362.7 R-N-K-R-A-K-N-S-L-E-L-E-L-S-D-D-D-E-D-D

795 G15 2305.6 N-K-R-A-K-N-S-L-E-L-E-L-S-D-D-D-E-D-D-V

796 G16 2304.7 K-R-A-K-N-S-L-E-L-E-L-S-D-D-D-E-D-D-V-L

797 G17 2304.6 R-A-K-N-S-L-E-L-E-L-S-D-D-D-E-D-D-V-L-Q

798 G18 2276.5 A-K-N-S-L-E-L-E-L-S-D-D-D-E-D-D-V-L-Q-Q

799 G19 2368.6 K-N-S-L-E-L-E-L-S-D-D-D-E-D-D-V-L-Q-Q-Y

800 G20 2396.6 N-S-L-E-L-E-L-S-D-D-D-E-D-D-V-L-Q-Q-Y-R

801 G21 2395.7 S-L-E-L-E-L-S-D-D-D-E-D-D-V-L-Q-Q-Y-R-L

802 G22 2436.8 L-E-L-E-L-S-D-D-D-E-D-D-V-L-Q-Q-Y-R-L-K

803 G23 2479.8 E-L-E-L-S-D-D-D-E-D-D-V-L-Q-Q-Y-R-L-K-R

804 G24 2506.9 L-E-L-S-D-D-D-E-D-D-V-L-Q-Q-Y-R-L-K-R-R

805 G25 2522.8 E-L-S-D-D-D-E-D-D-V-L-Q-Q-Y-R-L-K-R-R-E

806 G26 2506.9 L-S-D-D-D-E-D-D-V-L-Q-Q-Y-R-L-K-R-R-E-L

807 G27 2524.9 S-D-D-D-E-D-D-V-L-Q-Q-Y-R-L-K-R-R-E-L-M

808 G28 2594 D-D-D-E-D-D-V-L-Q-Q-Y-R-L-K-R-R-E-L-M-R

809 G29 2607.1 D-D-E-D-D-V-L-Q-Q-Y-R-L-K-R-R-E-L-M-R-K

810 G30 2648.2 D-E-D-D-V-L-Q-Q-Y-R-L-K-R-R-E-L-M-R-K-R

811 H 1 2689.3 E-D-D-V-L-Q-Q-Y-R-L-K-R-R-E-L-M-R-K-R-R

812 H 2 2673.4 D-D-V-L-Q-Q-Y-R-L-K-R-R-E-L-M-R-K-R-R-L

813 H 3 2687.4 D-V-L-Q-Q-Y-R-L-K-R-R-E-L-M-R-K-R-R-L-E

814 H 4 2685.5 V-L-Q-Q-Y-R-L-K-R-R-E-L-M-R-K-R-R-L-E-I

815 H 5 2643.5 L-Q-Q-Y-R-L-K-R-R-E-L-M-R-K-R-R-L-E-I-G

816 H 6 2645.4 Q-Q-Y-R-L-K-R-R-E-L-M-R-K-R-R-L-E-I-G-D

817 H 7 2632.4 Q-Y-R-L-K-R-R-E-L-M-R-K-R-R-L-E-I-G-D-D

818 H 8 2575.4 Y-R-L-K-R-R-E-L-M-R-K-R-R-L-E-I-G-D-D-A

819 H 9 2540.4 R-L-K-R-R-E-L-M-R-K-R-R-L-E-I-G-D-D-A-K

820 H10 2497.4 L-K-R-R-E-L-M-R-K-R-R-L-E-I-G-D-D-A-K-L

821 H11 2483.3 K-R-R-E-L-M-R-K-R-R-L-E-I-G-D-D-A-K-L-V

822 H12 2483.3 R-R-E-L-M-R-K-R-R-L-E-I-G-D-D-A-K-L-V-K

823 H13 2441.2 R-E-L-M-R-K-R-R-L-E-I-G-D-D-A-K-L-V-K-N

824 H14 2382.1 E-L-M-R-K-R-R-L-E-I-G-D-D-A-K-L-V-K-N-P

825 H15 2381.2 L-M-R-K-R-R-L-E-I-G-D-D-A-K-L-V-K-N-P-K

826 H16 2355.1 M-R-K-R-R-L-E-I-G-D-D-A-K-L-V-K-N-P-K-S

827 H17 2311 R-K-R-R-L-E-I-G-D-D-A-K-L-V-K-N-P-K-S-S

828 H18 2225.9 K-R-R-L-E-I-G-D-D-A-K-L-V-K-N-P-K-S-S-A

829 H19 2244.9 R-R-L-E-I-G-D-D-A-K-L-V-K-N-P-K-S-S-A-F

830 H20 2235.9 R-L-E-I-G-D-D-A-K-L-V-K-N-P-K-S-S-A-F-F

831 H21 2208.8 L-E-I-G-D-D-A-K-L-V-K-N-P-K-S-S-A-F-F-E

832 H22 2182.7 E-I-G-D-D-A-K-L-V-K-N-P-K-S-S-A-F-F-E-S

833 H23 2184.8 I-G-D-D-A-K-L-V-K-N-P-K-S-S-A-F-F-E-S-M

834 H24 2170.7 G-D-D-A-K-L-V-K-N-P-K-S-S-A-F-F-E-S-M-V

835 H25 2242.7 D-D-A-K-L-V-K-N-P-K-S-S-A-F-F-E-S-M-V-E

836 H26 2242.7 D-A-K-L-V-K-N-P-K-S-S-A-F-F-E-S-M-V-E-D

837 H27 2240.8 A-K-L-V-K-N-P-K-S-S-A-F-F-E-S-M-V-E-D-I

838 H28 2282.9 K-L-V-K-N-P-K-S-S-A-F-F-E-S-M-V-E-D-I-I

839 H29 2283.8 L-V-K-N-P-K-S-S-A-F-F-E-S-M-V-E-D-I-I-E

840 H30 2333.8 V-K-N-P-K-S-S-A-F-F-E-S-M-V-E-D-I-I-E-Y

841 I 1 2362.9 K-N-P-K-S-S-A-F-F-E-S-M-V-E-D-I-I-E-Y-K

842 I 2 2348.8 N-P-K-S-S-A-F-F-E-S-M-V-E-D-I-I-E-Y-K-N

843 I 3 2331.8 P-K-S-S-A-F-F-E-S-M-V-E-D-I-I-E-Y-K-N-P

844 I 4 2381.9 K-S-S-A-F-F-E-S-M-V-E-D-I-I-E-Y-K-N-P-F

845 I 5 2310.8 S-S-A-F-F-E-S-M-V-E-D-I-I-E-Y-K-N-P-F-G

846 I 6 2294.8 S-A-F-F-E-S-M-V-E-D-I-I-E-Y-K-N-P-F-G-A

847 I 7 2336.8 A-F-F-E-S-M-V-E-D-I-I-E-Y-K-N-P-F-G-A-E

848 I 8 2394.8 F-F-E-S-M-V-E-D-I-I-E-Y-K-N-P-F-G-A-E-E

849 I 9 2376.7 F-E-S-M-V-E-D-I-I-E-Y-K-N-P-F-G-A-E-E-E

850 I10 2392.7 E-S-M-V-E-D-I-I-E-Y-K-N-P-F-G-A-E-E-E-Y

851 I11 2377.7 S-M-V-E-D-I-I-E-Y-K-N-P-F-G-A-E-E-E-Y-N

852 I12 2403.8 M-V-E-D-I-I-E-Y-K-N-P-F-G-A-E-E-E-Y-N-L

853 I13 2387.7 V-E-D-I-I-E-Y-K-N-P-F-G-A-E-E-E-Y-N-L-D

854 I14 2401.8 E-D-I-I-E-Y-K-N-P-F-G-A-E-E-E-Y-N-L-D-I

855 I15 2373.8 D-I-I-E-Y-K-N-P-F-G-A-E-E-E-Y-N-L-D-I-T

856 I16 2345.8 I-I-E-Y-K-N-P-F-G-A-E-E-E-Y-N-L-D-I-T-S

857 I17 2333.7 I-E-Y-K-N-P-F-G-A-E-E-E-Y-N-L-D-I-T-S-T

858 I18 2291.6 E-Y-K-N-P-F-G-A-E-E-E-Y-N-L-D-I-T-S-T-A

859 I19 2263.6 Y-K-N-P-F-G-A-E-E-E-Y-N-L-D-I-T-S-T-A-T

860 I20 2215.5 K-N-P-F-G-A-E-E-E-Y-N-L-D-I-T-S-T-A-T-D

861 I21 2200.5 N-P-F-G-A-E-E-E-Y-N-L-D-I-T-S-T-A-T-D-L

862 I22 2201.5 P-F-G-A-E-E-E-Y-N-L-D-I-T-S-T-A-T-D-L-D

863 I23 2205.5 F-G-A-E-E-E-Y-N-L-D-I-T-S-T-A-T-D-L-D-T

864 I24 2186.4 G-A-E-E-E-Y-N-L-D-I-T-S-T-A-T-D-L-D-T-Q

865 I25 2244.4 A-E-E-E-Y-N-L-D-I-T-S-T-A-T-D-L-D-T-Q-D

866 I26 2287.4 E-E-E-Y-N-L-D-I-T-S-T-A-T-D-L-D-T-Q-D-N

867 I27 2245.4 E-E-Y-N-L-D-I-T-S-T-A-T-D-L-D-T-Q-D-N-S

868 I28 2229.5 E-Y-N-L-D-I-T-S-T-A-T-D-L-D-T-Q-D-N-S-I

869 I29 2214.5 Y-N-L-D-I-T-S-T-A-T-D-L-D-T-Q-D-N-S-I-N

870 I30 2150.4 N-L-D-I-T-S-T-A-T-D-L-D-T-Q-D-N-S-I-N-V

871 J 1 2093.4 L-D-I-T-S-T-A-T-D-L-D-T-Q-D-N-S-I-N-V-G

872 J 2 2095.3 D-I-T-S-T-A-T-D-L-D-T-Q-D-N-S-I-N-V-G-D

873 J 3 2094.3 I-T-S-T-A-T-D-L-D-T-Q-D-N-S-I-N-V-G-D-N

874 J 4 2082.2 T-S-T-A-T-D-L-D-T-Q-D-N-S-I-N-V-G-D-N-T

875 J 5 2038.2 S-T-A-T-D-L-D-T-Q-D-N-S-I-N-V-G-D-N-T-G

876 J 6 2065.2 T-A-T-D-L-D-T-Q-D-N-S-I-N-V-G-D-N-T-G-N

877 J 7 2078.2 A-T-D-L-D-T-Q-D-N-S-I-N-V-G-D-N-T-G-N-N

878 J 8 2136.2 T-D-L-D-T-Q-D-N-S-I-N-V-G-D-N-T-G-N-N-E

879 J 9 2163.2 D-L-D-T-Q-D-N-S-I-N-V-G-D-N-T-G-N-N-E-Q

880 J10 2176.3 L-D-T-Q-D-N-S-I-N-V-G-D-N-T-G-N-N-E-Q-K

881 J11 2160.2 D-T-Q-D-N-S-I-N-V-G-D-N-T-G-N-N-E-Q-K-P

882 J12 2144.2 T-Q-D-N-S-I-N-V-G-D-N-T-G-N-N-E-Q-K-P-V

883 J13 2158.2 Q-D-N-S-I-N-V-G-D-N-T-G-N-N-E-Q-K-P-V-D

884 J14 2158.2 D-N-S-I-N-V-G-D-N-T-G-N-N-E-Q-K-P-V-D-Q

885 J15 2171.3 N-S-I-N-V-G-D-N-T-G-N-N-E-Q-K-P-V-D-Q-K

886 J16 2171.3 S-I-N-V-G-D-N-T-G-N-N-E-Q-K-P-V-D-Q-K-N

887 J17 2212.4 I-N-V-G-D-N-T-G-N-N-E-Q-K-P-V-D-Q-K-N-K

888 J18 2227.4 N-V-G-D-N-T-G-N-N-E-Q-K-P-V-D-Q-K-N-K-K

889 J19 2212.4 V-G-D-N-T-G-N-N-E-Q-K-P-V-D-Q-K-N-K-K-V

890 J20 2226.5 G-D-N-T-G-N-N-E-Q-K-P-V-D-Q-K-N-K-K-V-I

891 J21 2282.6 D-N-T-G-N-N-E-Q-K-P-V-D-Q-K-N-K-K-V-I-I

892 J22 2254.6 N-T-G-N-N-E-Q-K-P-V-D-Q-K-N-K-K-V-I-I-S

893 J23 2269.6 T-G-N-N-E-Q-K-P-V-D-Q-K-N-K-K-V-I-I-S-E

894 J24 2283.6 G-N-N-E-Q-K-P-V-D-Q-K-N-K-K-V-I-I-S-E-D

895 J25 2373.7 N-N-E-Q-K-P-V-D-Q-K-N-K-K-V-I-I-S-E-D-F

896 J26 2358.7 N-E-Q-K-P-V-D-Q-K-N-K-K-V-I-I-S-E-D-F-V

897 J27 2372.7 E-Q-K-P-V-D-Q-K-N-K-K-V-I-I-S-E-D-F-V-Q

898 J28 2371.8 Q-K-P-V-D-Q-K-N-K-K-V-I-I-S-E-D-F-V-Q-K

899 J29 2330.8 K-P-V-D-Q-K-N-K-K-V-I-I-S-E-D-F-V-Q-K-S

900 J30 2315.8 P-V-D-Q-K-N-K-K-V-I-I-S-E-D-F-V-Q-K-S-L

901 K 1 2305.8 V-D-Q-K-N-K-K-V-I-I-S-E-D-F-V-Q-K-S-L-S

902 K 2 2353.9 D-Q-K-N-K-K-V-I-I-S-E-D-F-V-Q-K-S-L-S-F

903 K 3 2352 Q-K-N-K-K-V-I-I-S-E-D-F-V-Q-K-S-L-S-F-L

904 K 4 2352.1 K-N-K-K-V-I-I-S-E-D-F-V-Q-K-S-L-S-F-L-K

905 K 5 2311 N-K-K-V-I-I-S-E-D-F-V-Q-K-S-L-S-F-L-K-S

906 K 6 2311 K-K-V-I-I-S-E-D-F-V-Q-K-S-L-S-F-L-K-S-N

907 K 7 2296.9 K-V-I-I-S-E-D-F-V-Q-K-S-L-S-F-L-K-S-N-N

908 K 8 2331.9 V-I-I-S-E-D-F-V-Q-K-S-L-S-F-L-K-S-N-N-Y

909 K 9 2361.9 I-I-S-E-D-F-V-Q-K-S-L-S-F-L-K-S-N-N-Y-E

910 K10 2363.8 I-S-E-D-F-V-Q-K-S-L-S-F-L-K-S-N-N-Y-E-D

911 K11 2397.8 S-E-D-F-V-Q-K-S-L-S-F-L-K-S-N-N-Y-E-D-F

912 K12 2439.8 E-D-F-V-Q-K-S-L-S-F-L-K-S-N-N-Y-E-D-F-E

913 K13 2411.8 D-F-V-Q-K-S-L-S-F-L-K-S-N-N-Y-E-D-F-E-T

914 K14 2411.8 F-V-Q-K-S-L-S-F-L-K-S-N-N-Y-E-D-F-E-T-D

915 K15 2392.8 V-Q-K-S-L-S-F-L-K-S-N-N-Y-E-D-F-E-T-D-K

916 K16 2422.8 Q-K-S-L-S-F-L-K-S-N-N-Y-E-D-F-E-T-D-K-E

917 K17 2407.9 K-S-L-S-F-L-K-S-N-N-Y-E-D-F-E-T-D-K-E-L

918 K18 2366.8 S-L-S-F-L-K-S-N-N-Y-E-D-F-E-T-D-K-E-L-S

919 K19 2435.9 L-S-F-L-K-S-N-N-Y-E-D-F-E-T-D-K-E-L-S-R

920 K20 2435.9 S-F-L-K-S-N-N-Y-E-D-F-E-T-D-K-E-L-S-R-I

921 K21 2476.9 F-L-K-S-N-N-Y-E-D-F-E-T-D-K-E-L-S-R-I-Q

922 K22 2466.8 L-K-S-N-N-Y-E-D-F-E-T-D-K-E-L-S-R-I-Q-H

923 K23 2410.7 K-S-N-N-Y-E-D-F-E-T-D-K-E-L-S-R-I-Q-H-G

924 K24 2396.6 S-N-N-Y-E-D-F-E-T-D-K-E-L-S-R-I-Q-H-G-N

925 K25 2424.6 N-N-Y-E-D-F-E-T-D-K-E-L-S-R-I-Q-H-G-N-D

926 K26 2439.6 N-Y-E-D-F-E-T-D-K-E-L-S-R-I-Q-H-G-N-D-E

927 K27 2396.6 Y-E-D-F-E-T-D-K-E-L-S-R-I-Q-H-G-N-D-E-A

928 K28 2346.6 E-D-F-E-T-D-K-E-L-S-R-I-Q-H-G-N-D-E-A-I

929 K29 2346.6 D-F-E-T-D-K-E-L-S-R-I-Q-H-G-N-D-E-A-I-E

930 K30 2346.6 F-E-T-D-K-E-L-S-R-I-Q-H-G-N-D-E-A-I-E-D

931 L 1 2312.6 E-T-D-K-E-L-S-R-I-Q-H-G-N-D-E-A-I-E-D-L

932 L 2 2346.7 T-D-K-E-L-S-R-I-Q-H-G-N-D-E-A-I-E-D-L-Y

933 L 3 2346.7 D-K-E-L-S-R-I-Q-H-G-N-D-E-A-I-E-D-L-Y-T

934 L 4 2344.8 K-E-L-S-R-I-Q-H-G-N-D-E-A-I-E-D-L-Y-T-L

935 L 5 2344.8 E-L-S-R-I-Q-H-G-N-D-E-A-I-E-D-L-Y-T-L-K

936 L 6 2343.8 L-S-R-I-Q-H-G-N-D-E-A-I-E-D-L-Y-T-L-K-Q

937 L 7 2344.7 S-R-I-Q-H-G-N-D-E-A-I-E-D-L-Y-T-L-K-Q-N

938 L 8 2344.7 R-I-Q-H-G-N-D-E-A-I-E-D-L-Y-T-L-K-Q-N-S

939 L 9 2275.6 I-Q-H-G-N-D-E-A-I-E-D-L-Y-T-L-K-Q-N-S-S

940 L10 2275.6 Q-H-G-N-D-E-A-I-E-D-L-Y-T-L-K-Q-N-S-S-I

941 L11 2275.7 H-G-N-D-E-A-I-E-D-L-Y-T-L-K-Q-N-S-S-I-K

942 L12 2225.7 G-N-D-E-A-I-E-D-L-Y-T-L-K-Q-N-S-S-I-K-S

943 L13 2315.8 N-D-E-A-I-E-D-L-Y-T-L-K-Q-N-S-S-I-K-S-F

944 L14 2302.8 D-E-A-I-E-D-L-Y-T-L-K-Q-N-S-S-I-K-S-F-T

945 L15 2301.8 E-A-I-E-D-L-Y-T-L-K-Q-N-S-S-I-K-S-F-T-N

946 L16 2259.8 A-I-E-D-L-Y-T-L-K-Q-N-S-S-I-K-S-F-T-N-S

947 L17 2316.8 I-E-D-L-Y-T-L-K-Q-N-S-S-I-K-S-F-T-N-S-Q

948 L18 2304.7 E-D-L-Y-T-L-K-Q-N-S-S-I-K-S-F-T-N-S-Q-T

949 L19 2290.7 D-L-Y-T-L-K-Q-N-S-S-I-K-S-F-T-N-S-Q-T-D

950 L20 2262.7 L-Y-T-L-K-Q-N-S-S-I-K-S-F-T-N-S-Q-T-D-S

951 L21 2250.6 Y-T-L-K-Q-N-S-S-I-K-S-F-T-N-S-Q-T-D-S-T

952 L22 2188.5 T-L-K-Q-N-S-S-I-K-S-F-T-N-S-Q-T-D-S-T-T

953 L23 2174.5 L-K-Q-N-S-S-I-K-S-F-T-N-S-Q-T-D-S-T-T-S

954 L24 2189.5 K-Q-N-S-S-I-K-S-F-T-N-S-Q-T-D-S-T-T-S-K

955 L25 2162.4 Q-N-S-S-I-K-S-F-T-N-S-Q-T-D-S-T-T-S-K-T

956 L26 2133.4 N-S-S-I-K-S-F-T-N-S-Q-T-D-S-T-T-S-K-T-V

957 L27 2133.4 S-S-I-K-S-F-T-N-S-Q-T-D-S-T-T-S-K-T-V-N

958 L28 2147.4 S-I-K-S-F-T-N-S-Q-T-D-S-T-T-S-K-T-V-N-T

959 L29 2173.5 I-K-S-F-T-N-S-Q-T-D-S-T-T-S-K-T-V-N-T-I

960 L30 2173.5 K-S-F-T-N-S-Q-T-D-S-T-T-S-K-T-V-N-T-I-I

961 M 1 2160.4 S-F-T-N-S-Q-T-D-S-T-T-S-K-T-V-N-T-I-I-D

962 M 2 2186.5 F-T-N-S-Q-T-D-S-T-T-S-K-T-V-N-T-I-I-D-L

963 M 3 2168.4 T-N-S-Q-T-D-S-T-T-S-K-T-V-N-T-I-I-D-L-E

964 M 4 2195.5 N-S-Q-T-D-S-T-T-S-K-T-V-N-T-I-I-D-L-E-K

965 M 5 2237.6 S-Q-T-D-S-T-T-S-K-T-V-N-T-I-I-D-L-E-K-R

966 M 6 2247.6 Q-T-D-S-T-T-S-K-T-V-N-T-I-I-D-L-E-K-R-P

967 M 7 2248.6 T-D-S-T-T-S-K-T-V-N-T-I-I-D-L-E-K-R-P-E

968 M 8 2262.6 D-S-T-T-S-K-T-V-N-T-I-I-D-L-E-K-R-P-E-D

969 M 9 2276.6 S-T-T-S-K-T-V-N-T-I-I-D-L-E-K-R-P-E-D-E

970 M10 2304.6 T-T-S-K-T-V-N-T-I-I-D-L-E-K-R-P-E-D-E-D

971 M11 2332.6 T-S-K-T-V-N-T-I-I-D-L-E-K-R-P-E-D-E-D-E

972 M12 2330.6 S-K-T-V-N-T-I-I-D-L-E-K-R-P-E-D-E-D-E-V

973 M13 2372.6 K-T-V-N-T-I-I-D-L-E-K-R-P-E-D-E-D-E-V-E

974 M14 2358.5 T-V-N-T-I-I-D-L-E-K-R-P-E-D-E-D-E-V-E-N

975 M15 2314.5 V-N-T-I-I-D-L-E-K-R-P-E-D-E-D-E-V-E-N-G

976 M16 2330.5 N-T-I-I-D-L-E-K-R-P-E-D-E-D-E-V-E-N-G-D

977 M17 2317.5 T-I-I-D-L-E-K-R-P-E-D-E-D-E-V-E-N-G-D-T

978 M18 2303.5 I-I-D-L-E-K-R-P-E-D-E-D-E-V-E-N-G-D-T-S

979 M19 2303.5 I-D-L-E-K-R-P-E-D-E-D-E-V-E-N-G-D-T-S-L

980 M20 2289.4 D-L-E-K-R-P-E-D-E-D-E-V-E-N-G-D-T-S-L-V

981 M21 2231.4 L-E-K-R-P-E-D-E-D-E-V-E-N-G-D-T-S-L-V-G

982 M22 2217.3 E-K-R-P-E-D-E-D-E-V-E-N-G-D-T-S-L-V-G-V

983 M23 2235.4 K-R-P-E-D-E-D-E-V-E-N-G-D-T-S-L-V-G-V-F

984 M24 2235.4 R-P-E-D-E-D-E-V-E-N-G-D-T-S-L-V-G-V-F-K

985 M25 2216.3 P-E-D-E-D-E-V-E-N-G-D-T-S-L-V-G-V-F-K-H

986 M26 2216.3 E-D-E-D-E-V-E-N-G-D-T-S-L-V-G-V-F-K-H-P

987 M27 2174.3 D-E-D-E-V-E-N-G-D-T-S-L-V-G-V-F-K-H-P-S

988 M28 2172.4 E-D-E-V-E-N-G-D-T-S-L-V-G-V-F-K-H-P-S-I

989 M29 2156.5 D-E-V-E-N-G-D-T-S-L-V-G-V-F-K-H-P-S-I-I

990 M30 2169.6 E-V-E-N-G-D-T-S-L-V-G-V-F-K-H-P-S-I-I-K

991 N 1 2127.6 V-E-N-G-D-T-S-L-V-G-V-F-K-H-P-S-I-I-K-S

992 N 2 2175.7 E-N-G-D-T-S-L-V-G-V-F-K-H-P-S-I-I-K-S-F

993 N 3 2117.7 N-G-D-T-S-L-V-G-V-F-K-H-P-S-I-I-K-S-F-A

994 N 4 2090.7 G-D-T-S-L-V-G-V-F-K-H-P-S-I-I-K-S-F-A-S

995 N 5 2189.8 D-T-S-L-V-G-V-F-K-H-P-S-I-I-K-S-F-A-S-R

996 N 6 2175.8 T-S-L-V-G-V-F-K-H-P-S-I-I-K-S-F-A-S-R-T

997 N 7 2189.8 S-L-V-G-V-F-K-H-P-S-I-I-K-S-F-A-S-R-T-D

998 N 8 2215.9 L-V-G-V-F-K-H-P-S-I-I-K-S-F-A-S-R-T-D-I

999 N 9 2216.8 V-G-V-F-K-H-P-S-I-I-K-S-F-A-S-R-T-D-I-N

1000 N10 2232.8 G-V-F-K-H-P-S-I-I-K-S-F-A-S-R-T-D-I-N-D

1001 N11 2303.9 V-F-K-H-P-S-I-I-K-S-F-A-S-R-T-D-I-N-D-K

1002 N12 2352 F-K-H-P-S-I-I-K-S-F-A-S-R-T-D-I-N-D-K-F

1003 N13 2333 K-H-P-S-I-I-K-S-F-A-S-R-T-D-I-N-D-K-F-K

1004 N14 2333.9 H-P-S-I-I-K-S-F-A-S-R-T-D-I-N-D-K-F-K-E

1005 N15 2253.9 P-S-I-I-K-S-F-A-S-R-T-D-I-N-D-K-F-K-E-G

1006 N16 2270.9 S-I-I-K-S-F-A-S-R-T-D-I-N-D-K-F-K-E-G-N

1007 N17 2312 I-I-K-S-F-A-S-R-T-D-I-N-D-K-F-K-E-G-N-K

1008 N18 2299.9 I-K-S-F-A-S-R-T-D-I-N-D-K-F-K-E-G-N-K-T

1009 N19 2285.8 K-S-F-A-S-R-T-D-I-N-D-K-F-K-E-G-N-K-T-V

1010 N20 2285.8 S-F-A-S-R-T-D-I-N-D-K-F-K-E-G-N-K-T-V-K

1011 N21 2311.9 F-A-S-R-T-D-I-N-D-K-F-K-E-G-N-K-T-V-K-I

1012 N22 2277.9 A-S-R-T-D-I-N-D-K-F-K-E-G-N-K-T-V-K-I-L

1013 N23 2335 S-R-T-D-I-N-D-K-F-K-E-G-N-K-T-V-K-I-L-K

1014 N24 2335 R-T-D-I-N-D-K-F-K-E-G-N-K-T-V-K-I-L-K-S

1015 N25 2342 T-D-I-N-D-K-F-K-E-G-N-K-T-V-K-I-L-K-S-Y

1016 N26 2369.1 D-I-N-D-K-F-K-E-G-N-K-T-V-K-I-L-K-S-Y-K

1017 N27 2355.1 I-N-D-K-F-K-E-G-N-K-T-V-K-I-L-K-S-Y-K-T

1018 N28 2341 N-D-K-F-K-E-G-N-K-T-V-K-I-L-K-S-Y-K-T-V

1019 N29 2284 D-K-F-K-E-G-N-K-T-V-K-I-L-K-S-Y-K-T-V-G

1020 N30 2256 K-F-K-E-G-N-K-T-V-K-I-L-K-S-Y-K-T-V-G-S

1021 O 1 2214.9 F-K-E-G-N-K-T-V-K-I-L-K-S-Y-K-T-V-G-S-S

1022 O 2 2195.9 K-E-G-N-K-T-V-K-I-L-K-S-Y-K-T-V-G-S-S-K

1023 O 3 2138.8 E-G-N-K-T-V-K-I-L-K-S-Y-K-T-V-G-S-S-K-A

1024 O 4 2096.8 G-N-K-T-V-K-I-L-K-S-Y-K-T-V-G-S-S-K-A-S

1025 O 5 2152.9 N-K-T-V-K-I-L-K-S-Y-K-T-V-G-S-S-K-A-S-I

1026 O 6 2139.9 K-T-V-K-I-L-K-S-Y-K-T-V-G-S-S-K-A-S-I-T

1027 O 7 2174.9 T-V-K-I-L-K-S-Y-K-T-V-G-S-S-K-A-S-I-T-Y

1028 O 8 2205 V-K-I-L-K-S-Y-K-T-V-G-S-S-K-A-S-I-T-Y-M

1029 O 9 2163 K-I-L-K-S-Y-K-T-V-G-S-S-K-A-S-I-T-Y-M-G

1030 O10 2163 I-L-K-S-Y-K-T-V-G-S-S-K-A-S-I-T-Y-M-G-K

1031 O11 2150.9 L-K-S-Y-K-T-V-G-S-S-K-A-S-I-T-Y-M-G-K-T

1032 O12 2193.9 K-S-Y-K-T-V-G-S-S-K-A-S-I-T-Y-M-G-K-T-R

1033 O13 2193.9 S-Y-K-T-V-G-S-S-K-A-S-I-T-Y-M-G-K-T-R-K

1034 O14 2220 Y-K-T-V-G-S-S-K-A-S-I-T-Y-M-G-K-T-R-K-L

1035 O15 2170 K-T-V-G-S-S-K-A-S-I-T-Y-M-G-K-T-R-K-L-I

1036 O16 2112.9 T-V-G-S-S-K-A-S-I-T-Y-M-G-K-T-R-K-L-I-A

1037 O17 2108.9 V-G-S-S-K-A-S-I-T-Y-M-G-K-T-R-K-L-I-A-P

1038 O18 2138 G-S-S-K-A-S-I-T-Y-M-G-K-T-R-K-L-I-A-P-K

1039 O19 2237.1 S-S-K-A-S-I-T-Y-M-G-K-T-R-K-L-I-A-P-K-R

1040 O20 2278.2 S-K-A-S-I-T-Y-M-G-K-T-R-K-L-I-A-P-K-R-K

1041 O21 2292.2 K-A-S-I-T-Y-M-G-K-T-R-K-L-I-A-P-K-R-K-T

1042 O22 2293.1 A-S-I-T-Y-M-G-K-T-R-K-L-I-A-P-K-R-K-T-E

1043 O23 2279.1 S-I-T-Y-M-G-K-T-R-K-L-I-A-P-K-R-K-T-E-G

1044 O24 2279.1 I-T-Y-M-G-K-T-R-K-L-I-A-P-K-R-K-T-E-G-S

1045 O25 2303 T-Y-M-G-K-T-R-K-L-I-A-P-K-R-K-T-E-G-S-H

1046 O26 2358.1 Y-M-G-K-T-R-K-L-I-A-P-K-R-K-T-E-G-S-H-R

1047 O27 2358.1 M-G-K-T-R-K-L-I-A-P-K-R-K-T-E-G-S-H-R-Y

1048 O28 2364 G-K-T-R-K-L-I-A-P-K-R-K-T-E-G-S-H-R-Y-H

1049 O29 2444 K-T-R-K-L-I-A-P-K-R-K-T-E-G-S-H-R-Y-H-H

1050 O30 2430.9 T-R-K-L-I-A-P-K-R-K-T-E-G-S-H-R-Y-H-H-D

1051 P 1 2466.9 R-K-L-I-A-P-K-R-K-T-E-G-S-H-R-Y-H-H-D-H

1052 P 2 2447.8 K-L-I-A-P-K-R-K-T-E-G-S-H-R-Y-H-H-D-H-H

1053 P 3 2433.7 L-I-A-P-K-R-K-T-E-G-S-H-R-Y-H-H-D-H-H-N

1054 P 4 2448.7 I-A-P-K-R-K-T-E-G-S-H-R-Y-H-H-D-H-H-N-K

1055 P 5 2463.7 A-P-K-R-K-T-E-G-S-H-R-Y-H-H-D-H-H-N-K-K

1056 P 6 2523.8 P-K-R-K-T-E-G-S-H-R-Y-H-H-D-H-H-N-K-K-M

1057 P 7 2554.9 K-R-K-T-E-G-S-H-R-Y-H-H-D-H-H-N-K-K-M-K

1058 P 8 2557.9 R-K-T-E-G-S-H-R-Y-H-H-D-H-H-N-K-K-M-K-M

1059 P 9 2529.9 K-T-E-G-S-H-R-Y-H-H-D-H-H-N-K-K-M-K-M-K

1060 P10 2502.8 T-E-G-S-H-R-Y-H-H-D-H-H-N-K-K-M-K-M-K-T

1061 P11 2529.9 E-G-S-H-R-Y-H-H-D-H-H-N-K-K-M-K-M-K-T-K

1062 P12 2501.9 G-S-H-R-Y-H-H-D-H-H-N-K-K-M-K-M-K-T-K-T

1063 P13 2573 S-H-R-Y-H-H-D-H-H-N-K-K-M-K-M-K-T-K-T-K

1064 P14 2573 H-R-Y-H-H-D-H-H-N-K-K-M-K-M-K-T-K-T-K-S

1065 P15 2550 R-Y-H-H-D-H-H-N-K-K-M-K-M-K-T-K-T-K-S-N

1066 P16 2522 Y-H-H-D-H-H-N-K-K-M-K-M-K-T-K-T-K-S-N-K

1067 P17 2472 H-H-D-H-H-N-K-K-M-K-M-K-T-K-T-K-S-N-K-L

1068 P18 2482.1 H-D-H-H-N-K-K-M-K-M-K-T-K-T-K-S-N-K-L-F

1069 P19 2474.1 D-H-H-N-K-K-M-K-M-K-T-K-T-K-S-N-K-L-F-E

1070 P20 2446.1 H-H-N-K-K-M-K-M-K-T-K-T-K-S-N-K-L-F-E-S

1071 P21 2366.1 H-N-K-K-M-K-M-K-T-K-T-K-S-N-K-L-F-E-S-G

1072 P22 2357.1 N-K-K-M-K-M-K-T-K-T-K-S-N-K-L-F-E-S-G-Q

1073 P23 2358.1 K-K-M-K-M-K-T-K-T-K-S-N-K-L-F-E-S-G-Q-D

1074 P24 2317 K-M-K-M-K-T-K-T-K-S-N-K-L-F-E-S-G-Q-D-S

1075 P25 2336 M-K-M-K-T-K-T-K-S-N-K-L-F-E-S-G-Q-D-S-F

1076 P26 2319.9 K-M-K-T-K-T-K-S-N-K-L-F-E-S-G-Q-D-S-F-D

1077 P27 2305.8 M-K-T-K-T-K-S-N-K-L-F-E-S-G-Q-D-S-F-D-N
