# Supplementary material for: Rad53 checkpoint kinase regulation of DNA replication fork rate via Mrc1 phosphorylation
Source: eLife. 2021 Aug 13;10:e69726. doi: 10.7554/eLife.69726 (PMC8387023; doi:10.7554/eLife.69726)

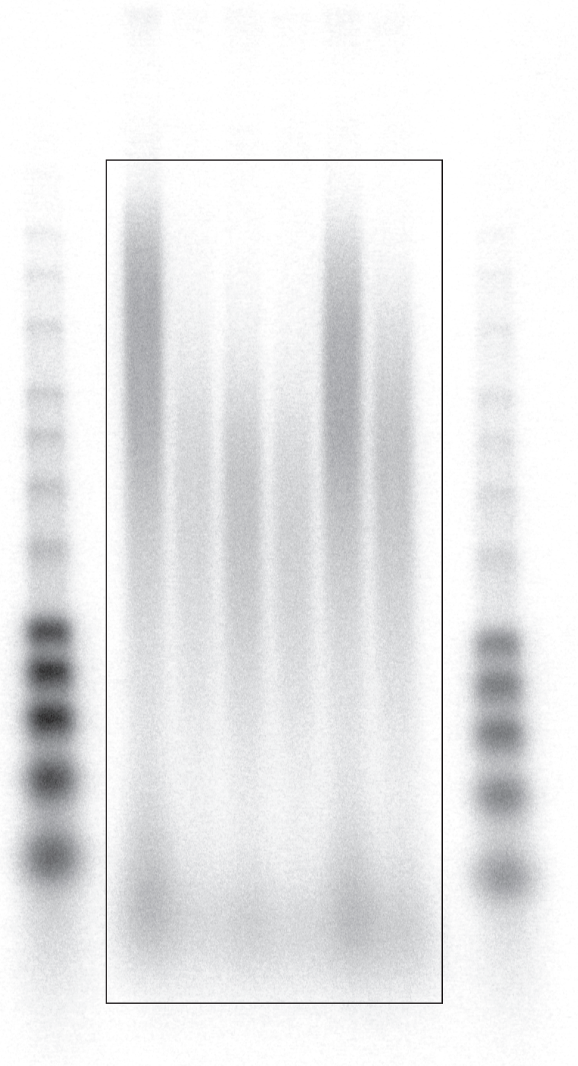

cropped area for Figure 6A

Figure 6 - source data 1.pdf

2500 x 2000

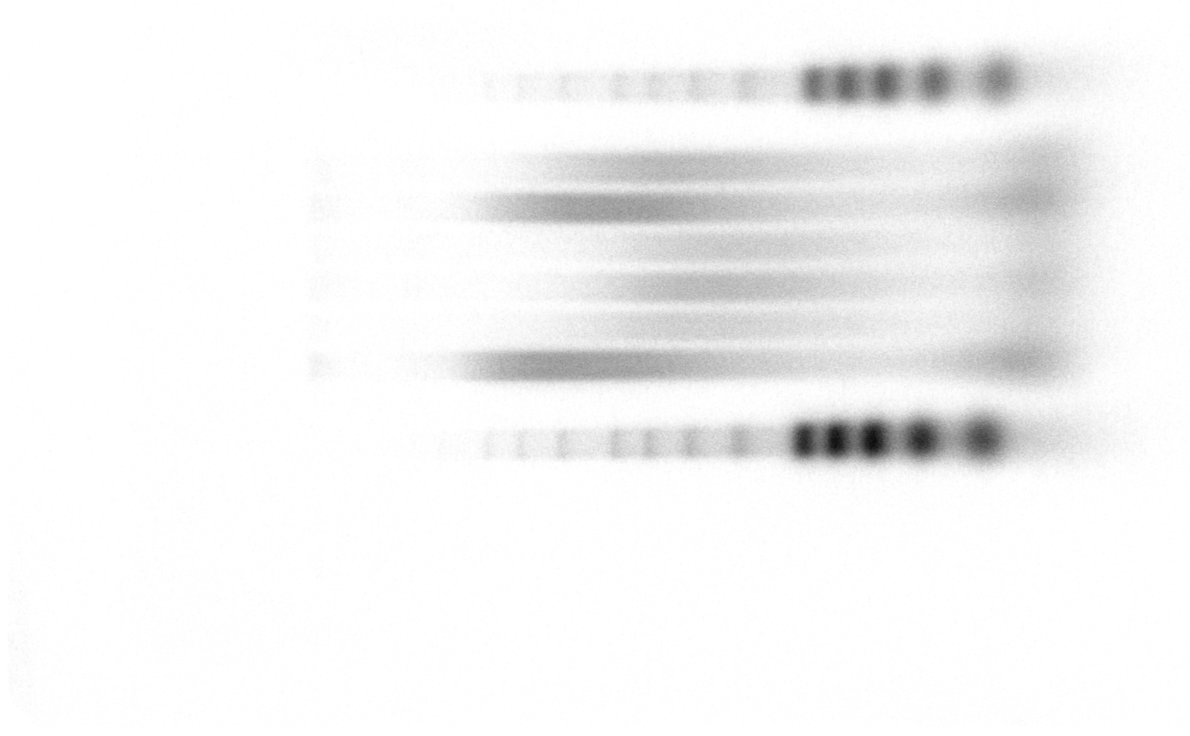

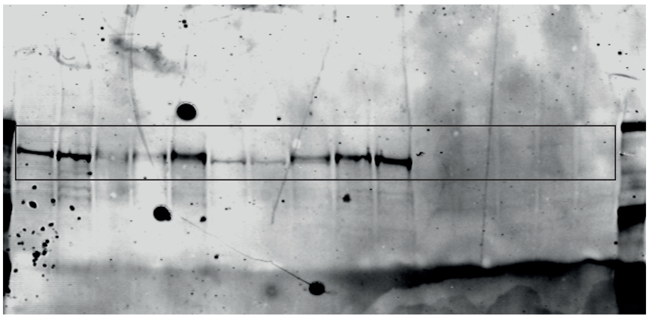

cropped area for Figure 6B (flag)

Figure 6 - source data 2.pdf

2005 x 968

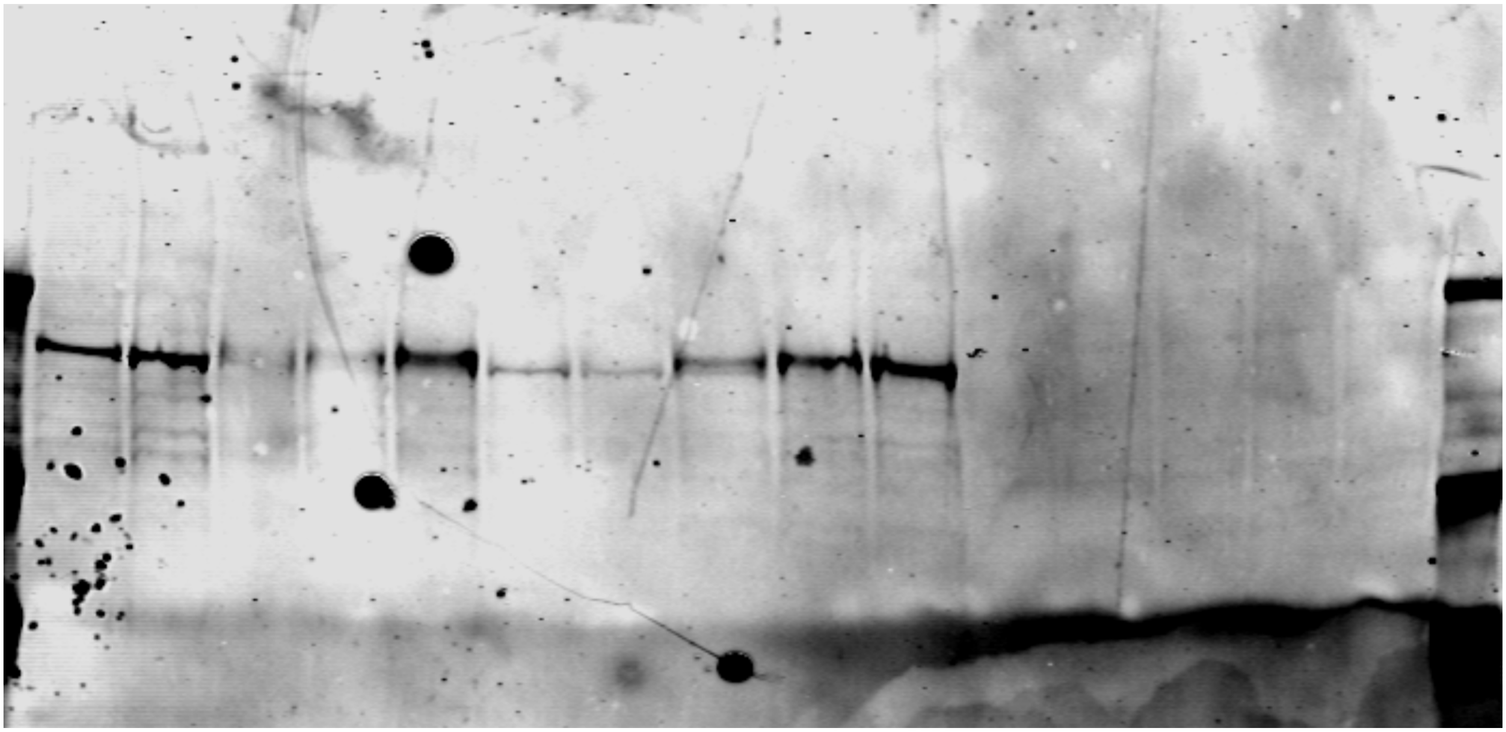

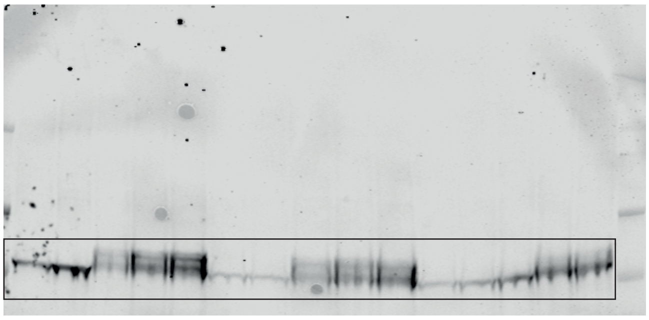

cropped area for Figure 6B (Rad53)

Figure 6 - source data 3.pdf

2005 x 968

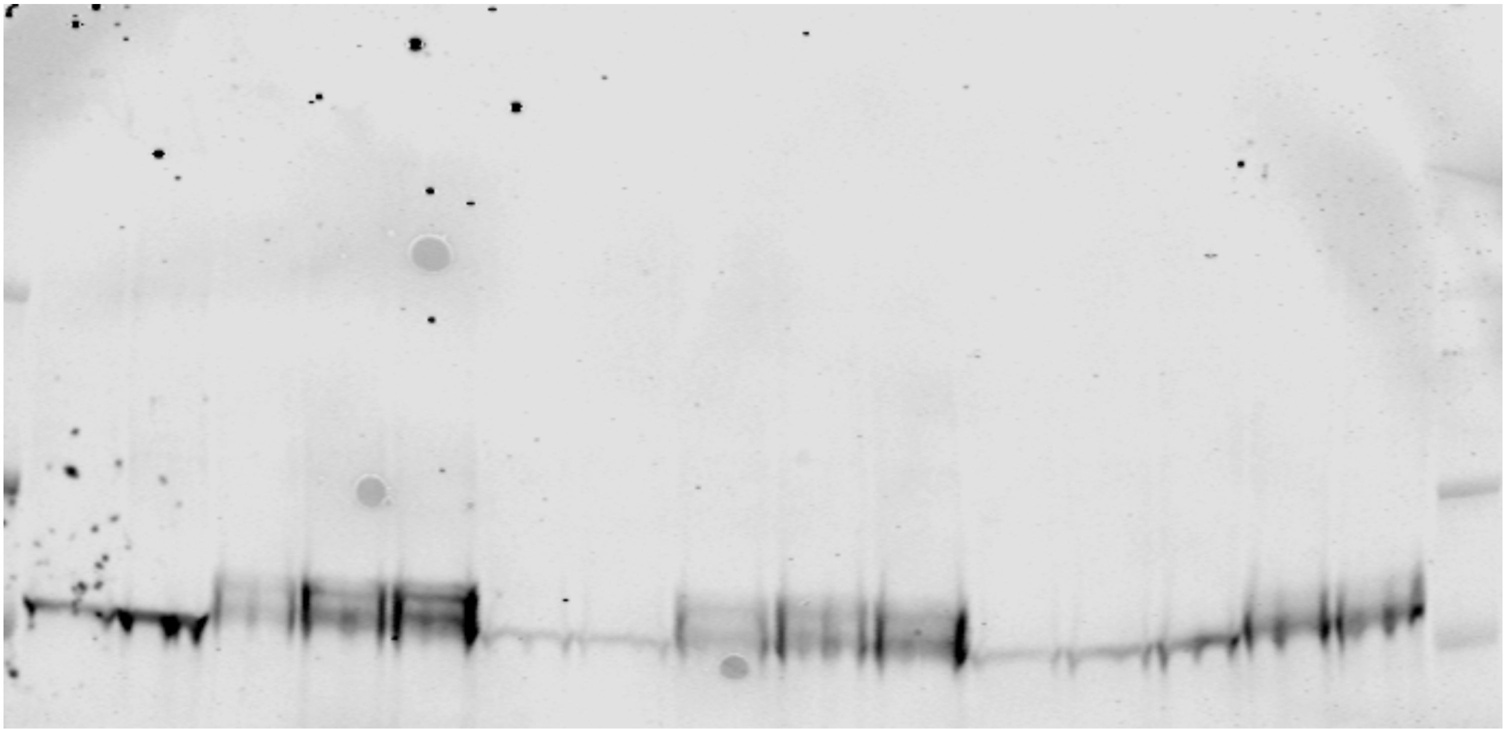

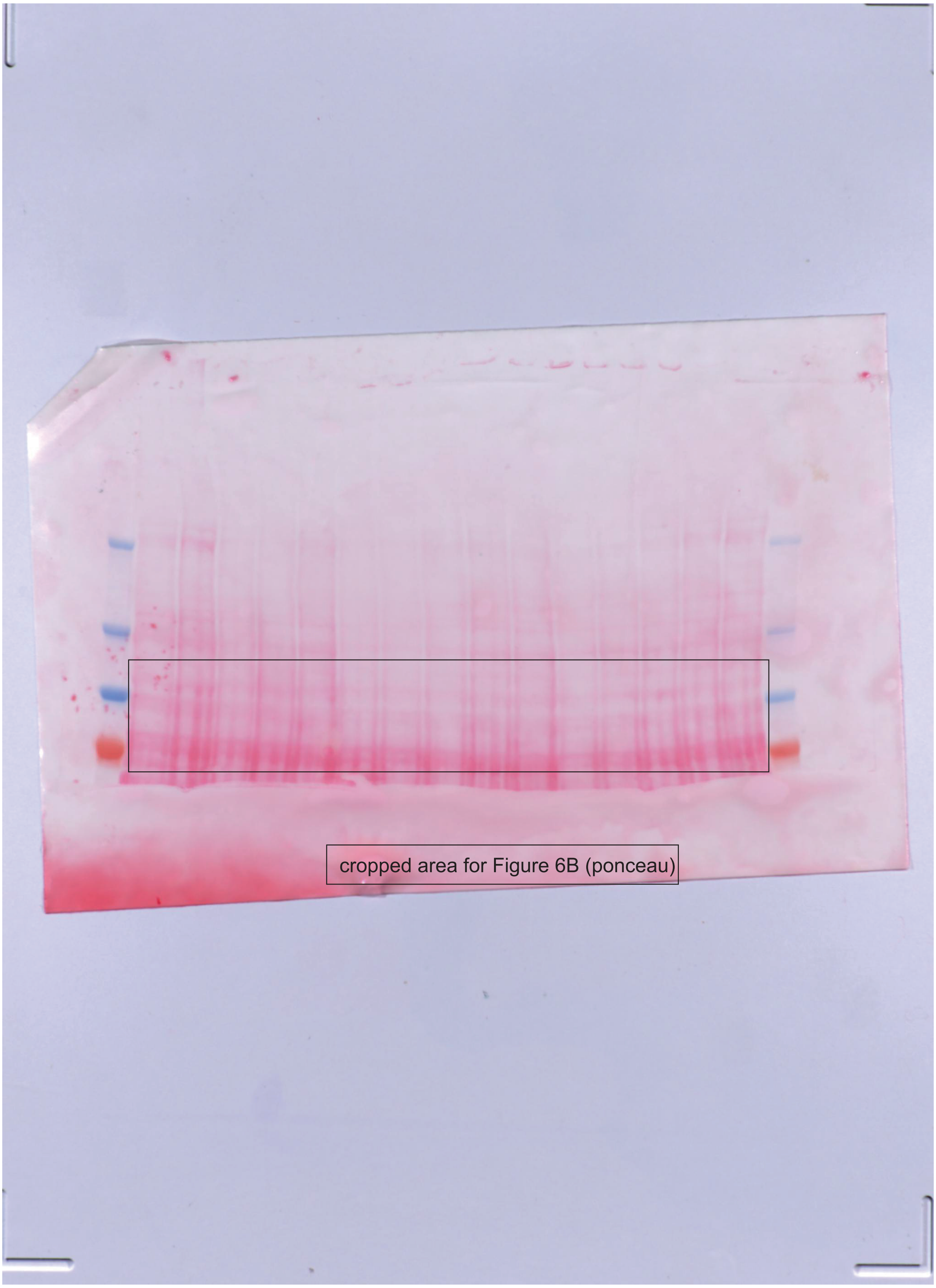
cropped area for Figure 6B (ponceau)

Figure 6 - source data 4.pdf

2048 x 2816

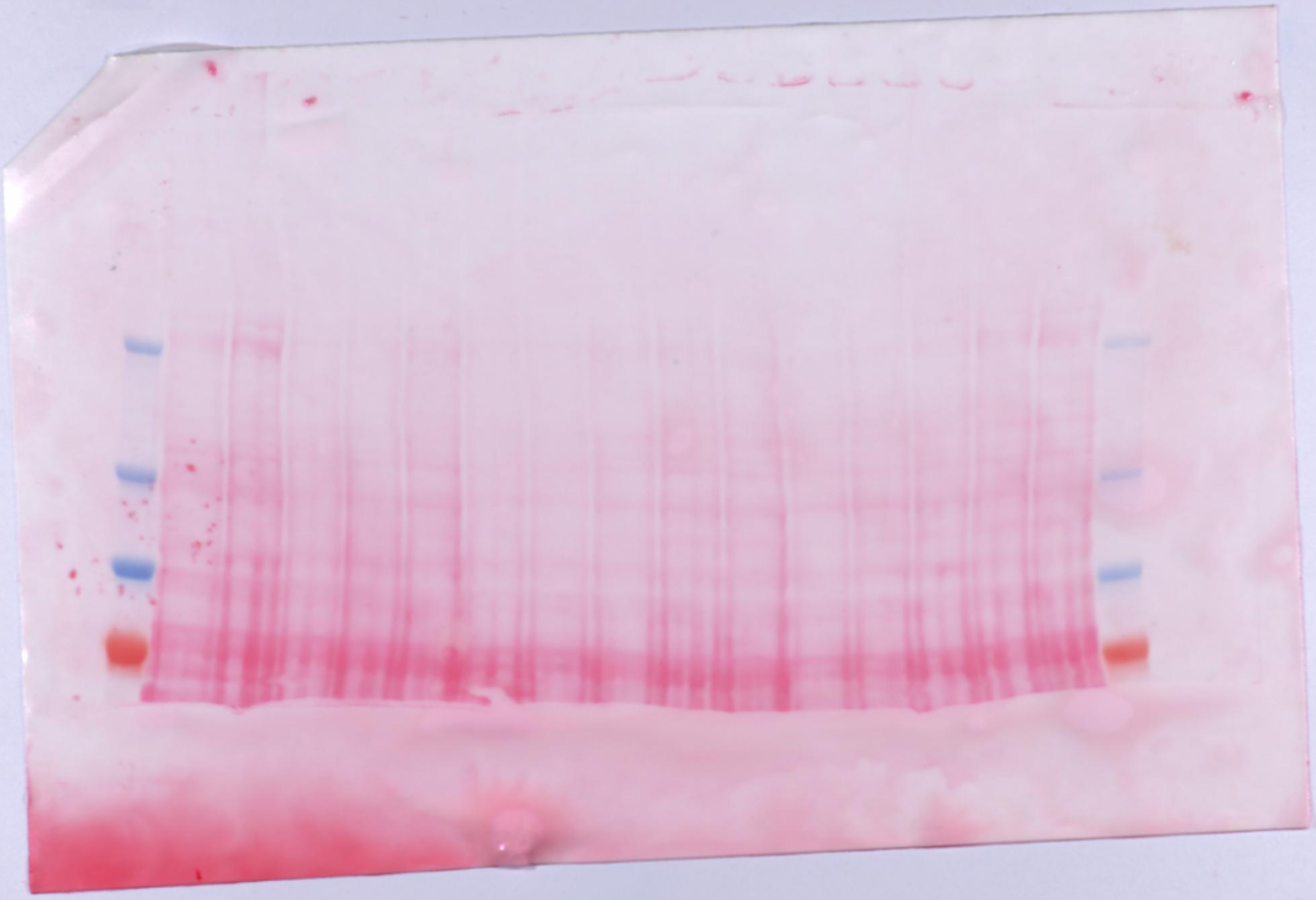

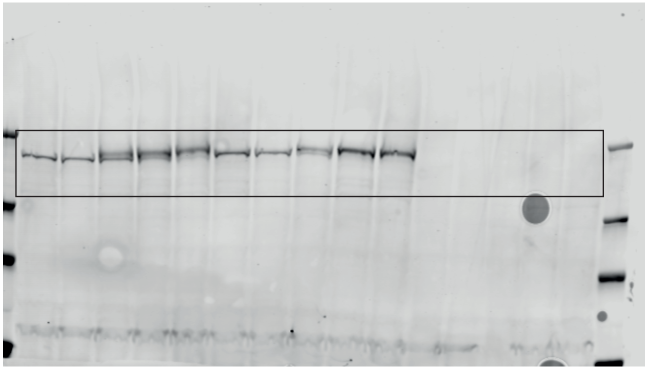

cropped area for Figure 6C (flag)

Figure 6 - source data 5.pdf

2005 x 1136

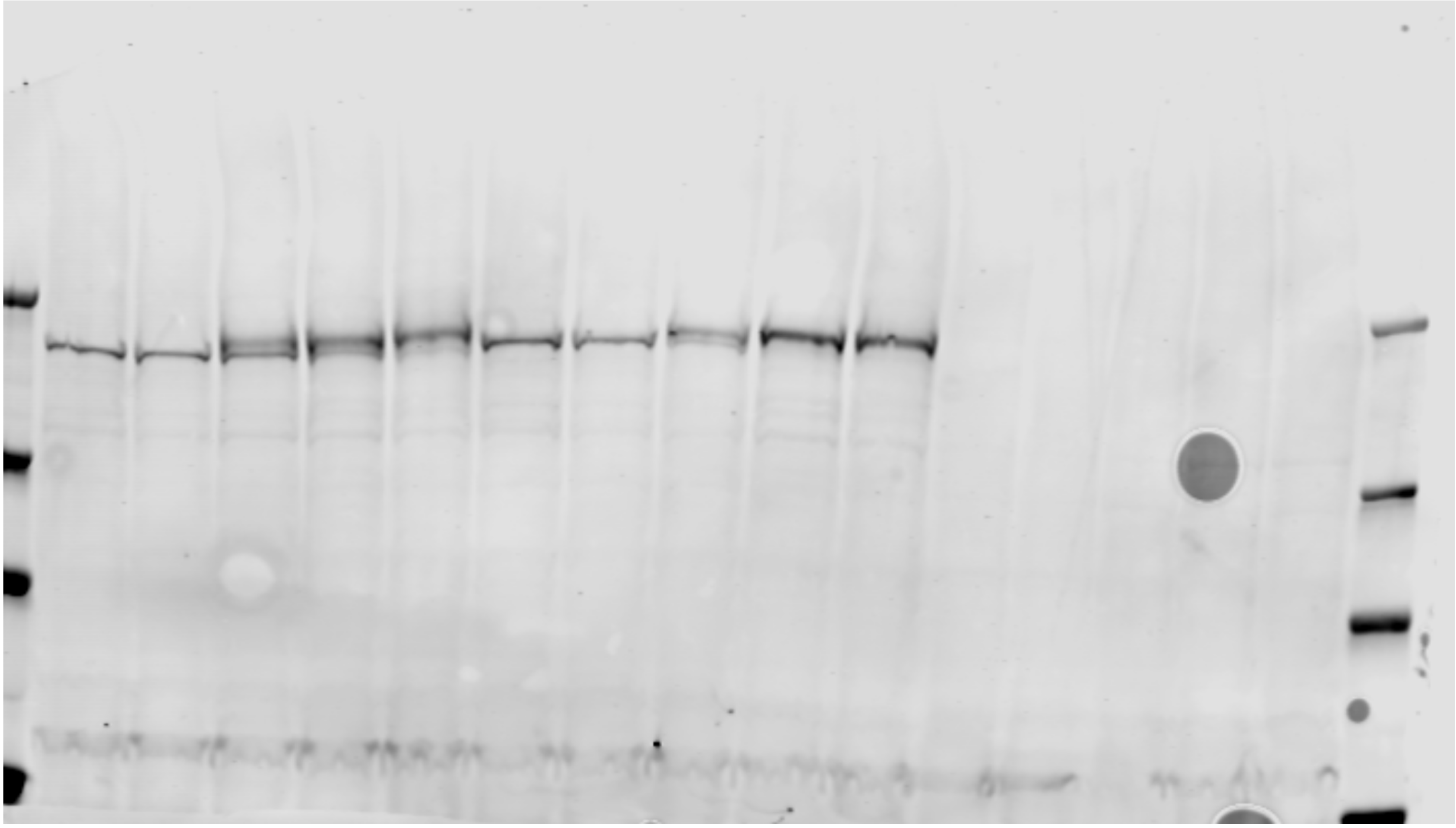

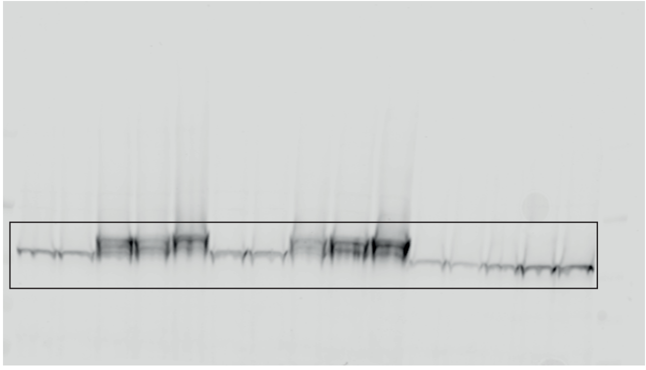

cropped area for Figure 6C (Rad53)

Figure 6 - source data 6.pdf

2005 x 1136

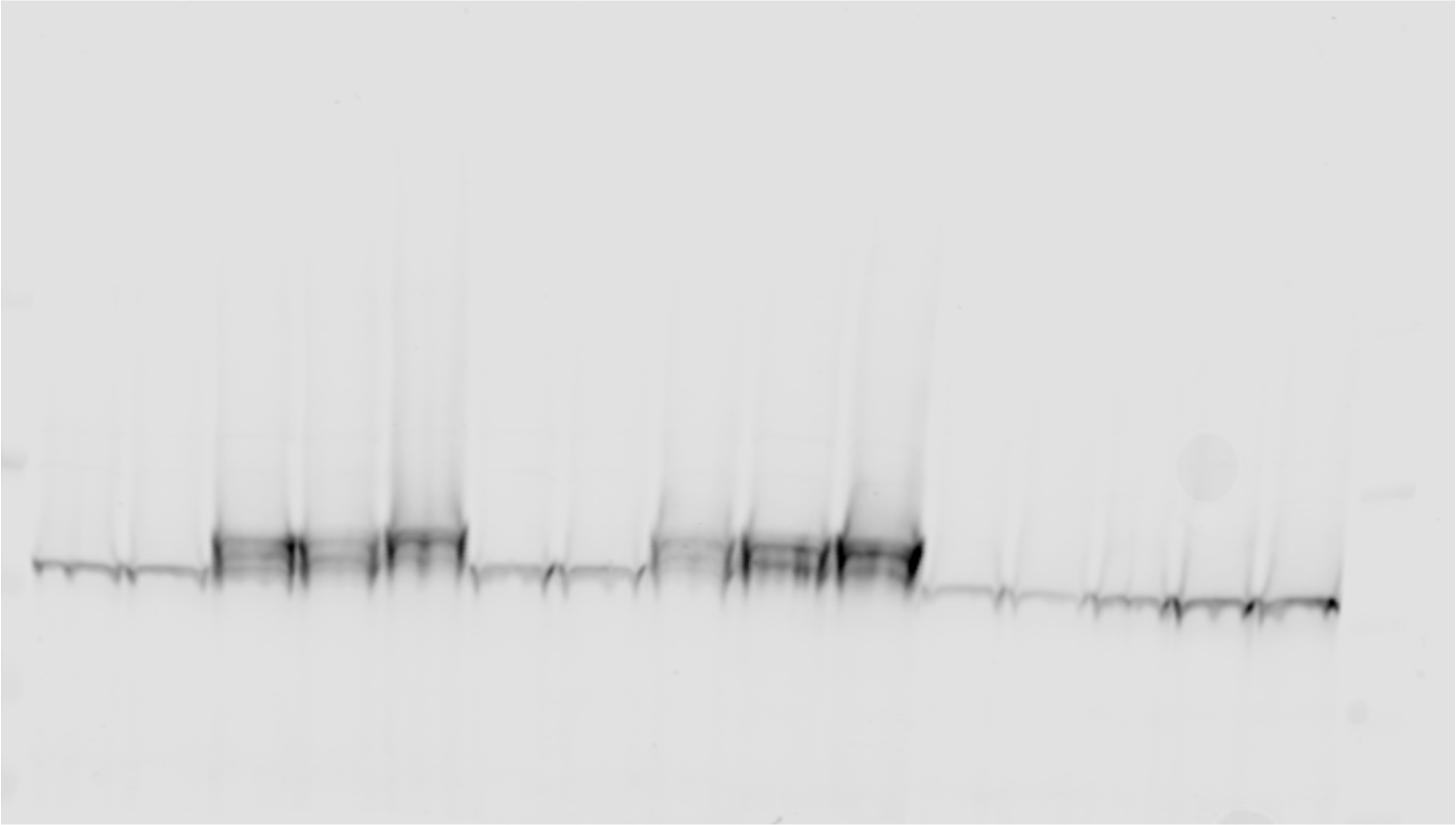

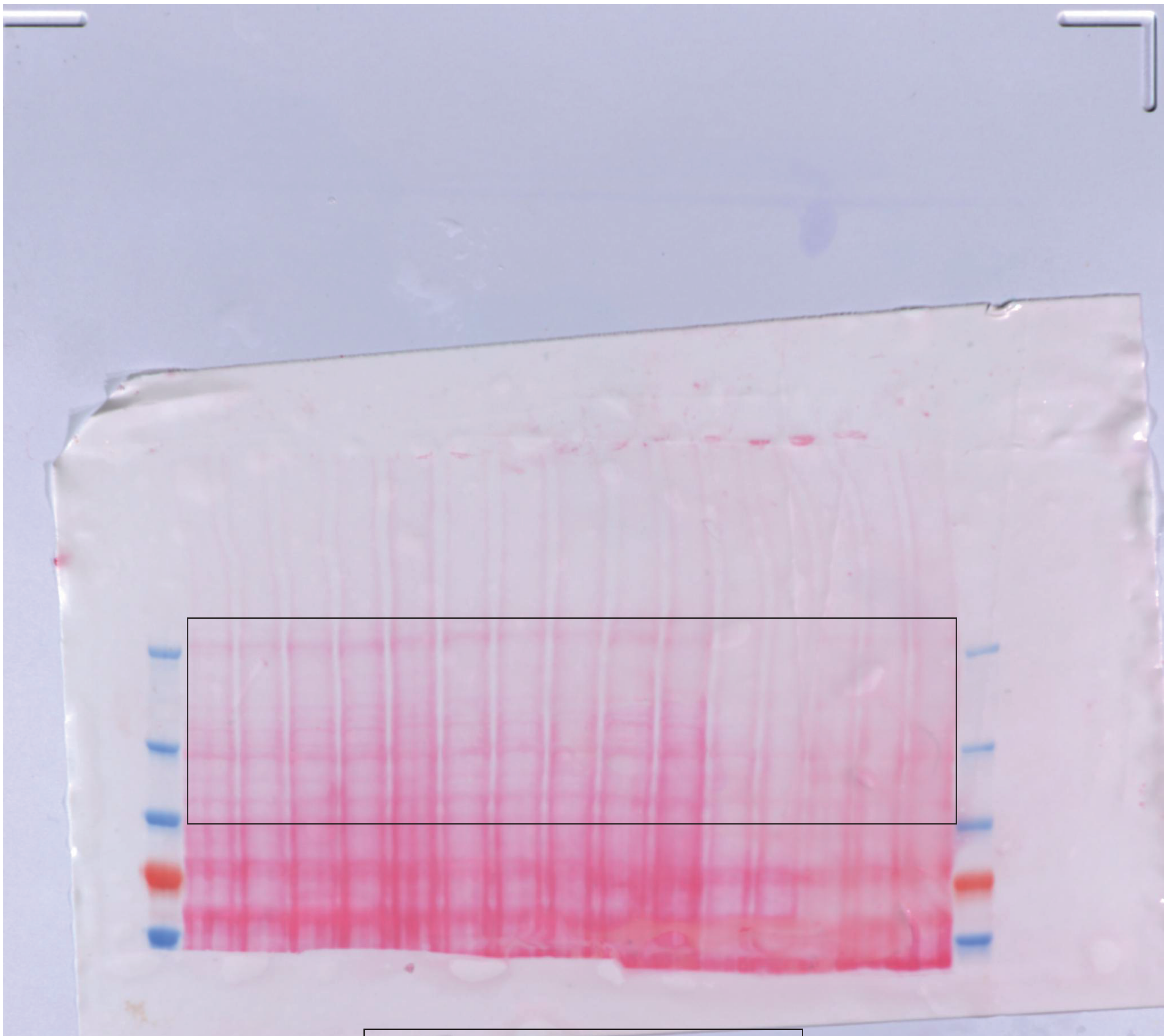

cropped area for Figure 6C (ponceau)

Figure 6 - source data 7.pdf

2048 x 2816

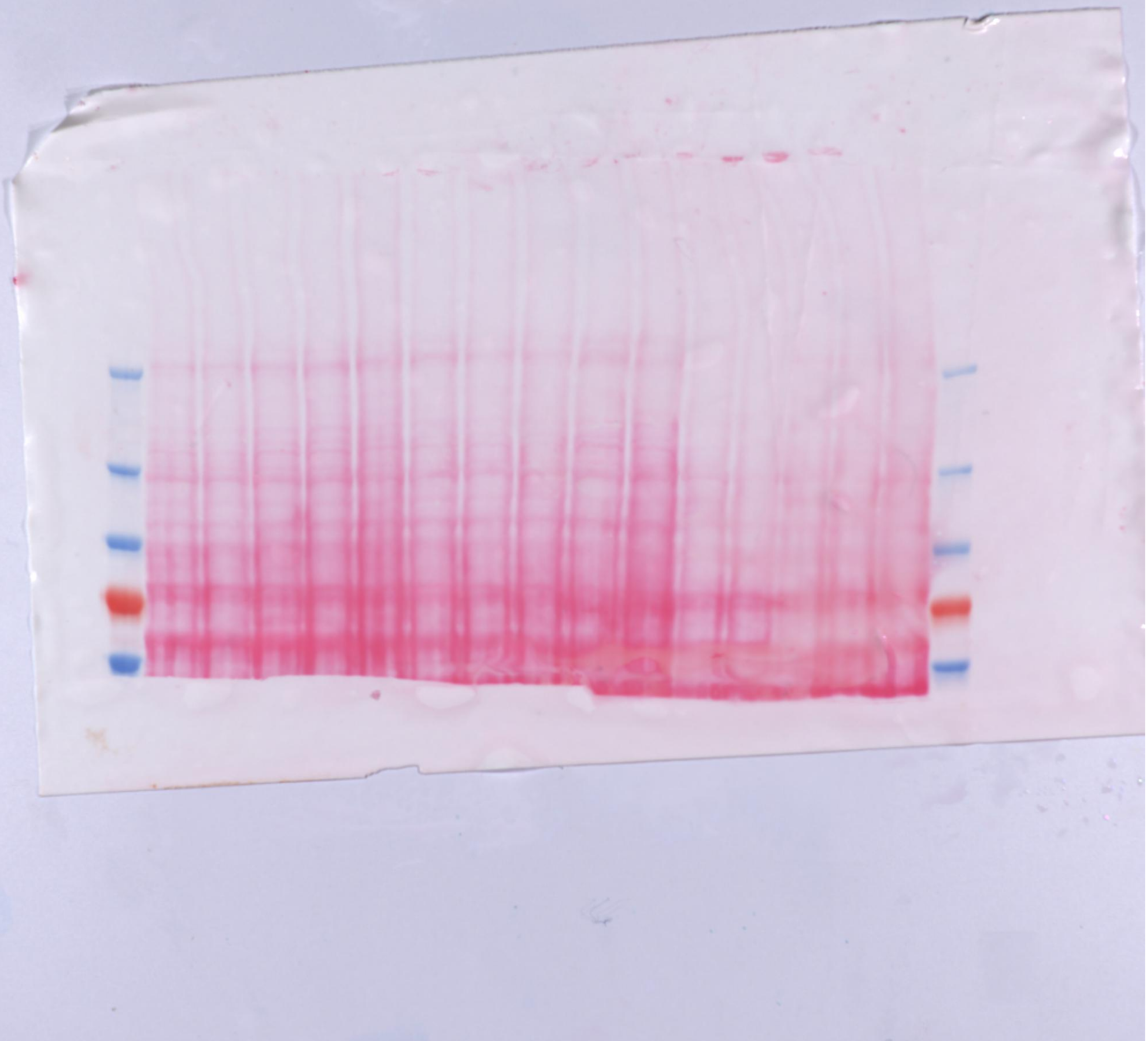

Figure 6 - source data 8.pdf
2048 x 2816

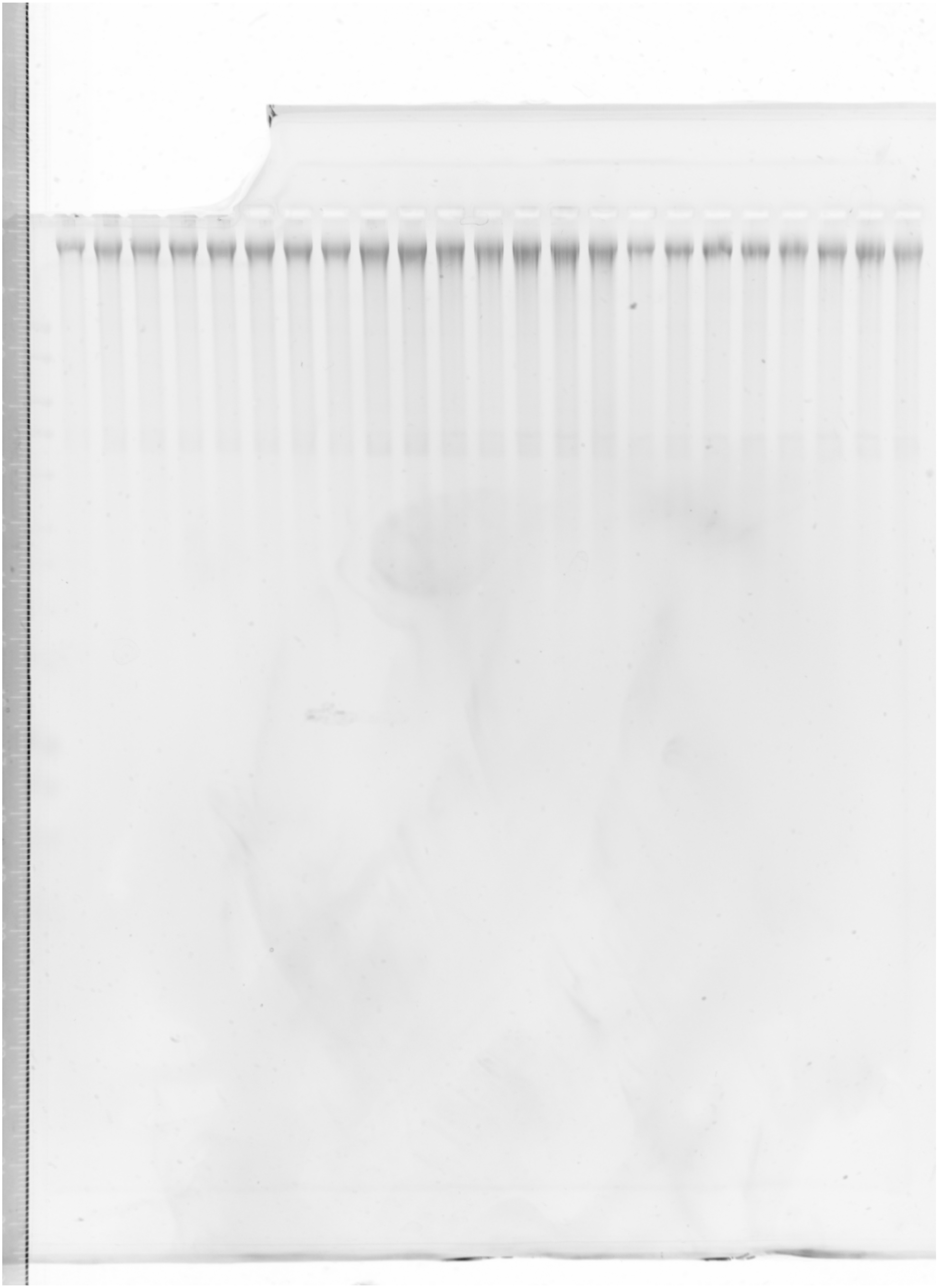

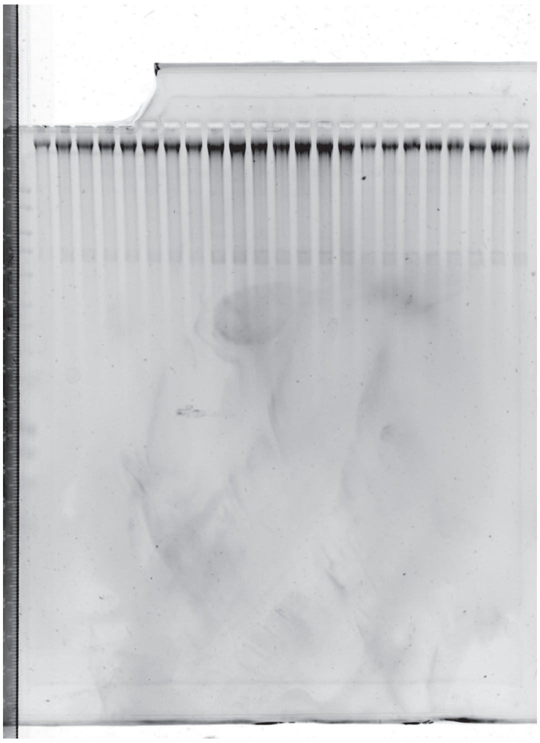

ethidium bromide stain to align bp ladder

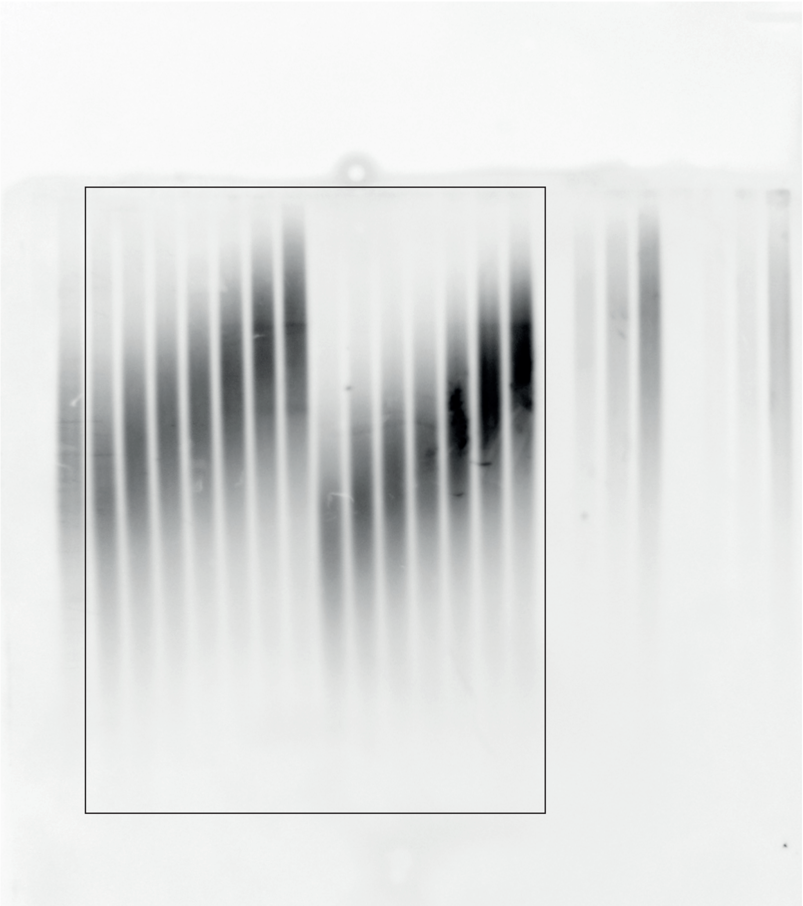

cropped area for Figure 6D

Figure 6 - source data 9.pdf

2048 x 2816

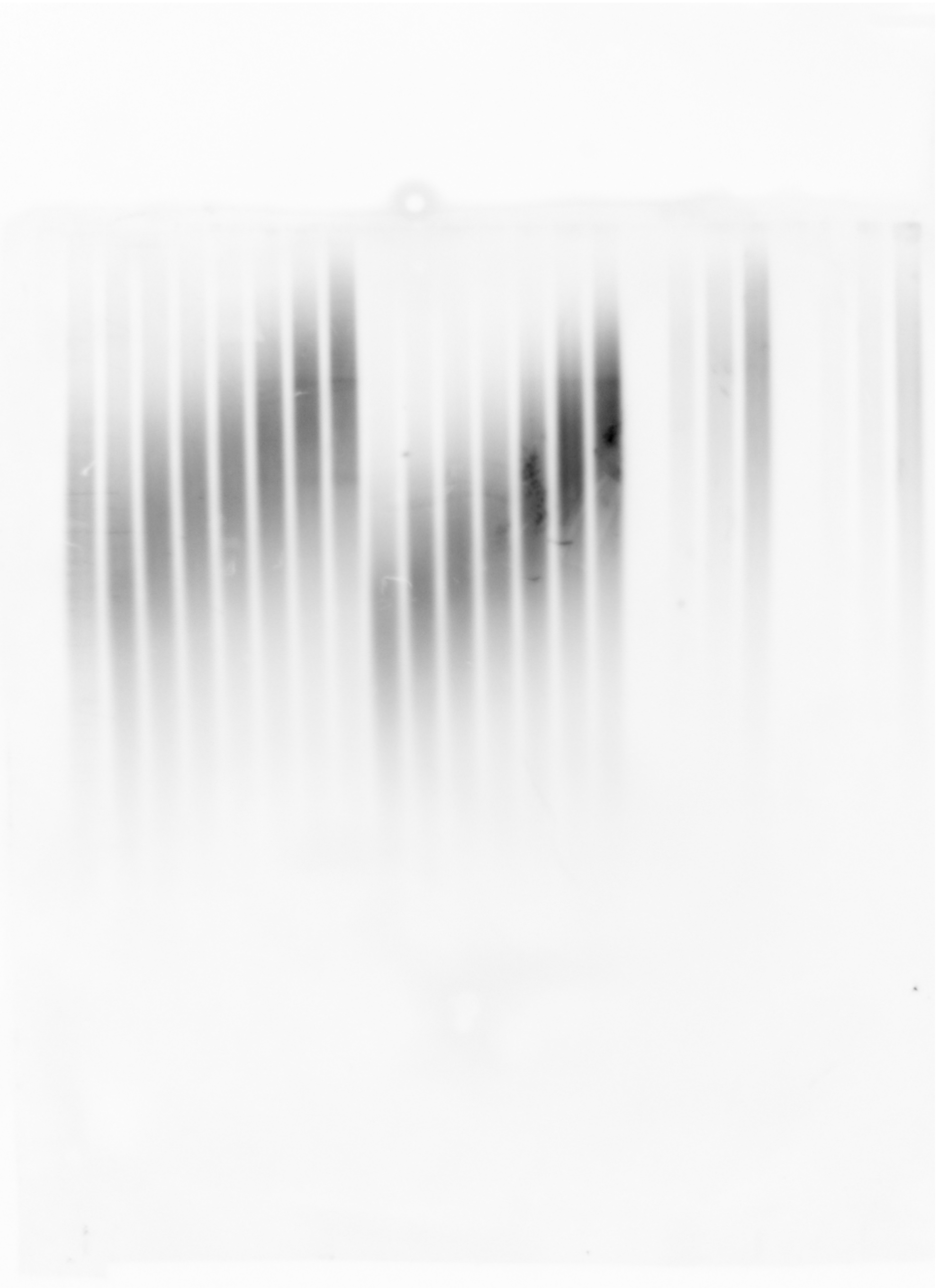

Supplement: Figure 6—source data 1. [file elife-69726-fig6-data1.pdf]
