# Supplementary material for: Rad53 checkpoint kinase regulation of DNA replication fork rate via Mrc1 phosphorylation
Source: eLife. 2021 Aug 13;10:e69726. doi: 10.7554/eLife.69726 (PMC8387023; doi:10.7554/eLife.69726)

Figure 7 - source data 1.pdf
2048 x 2816

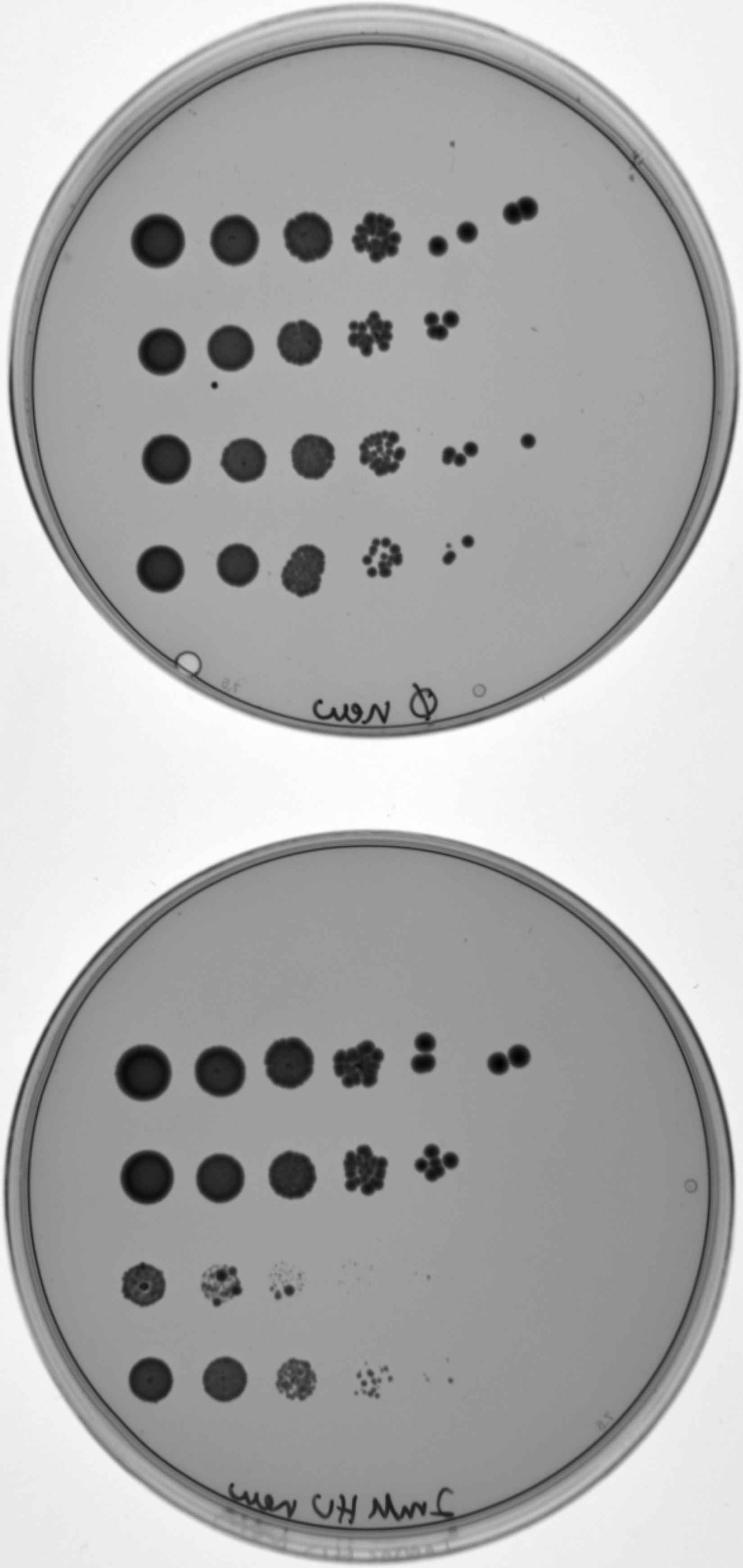

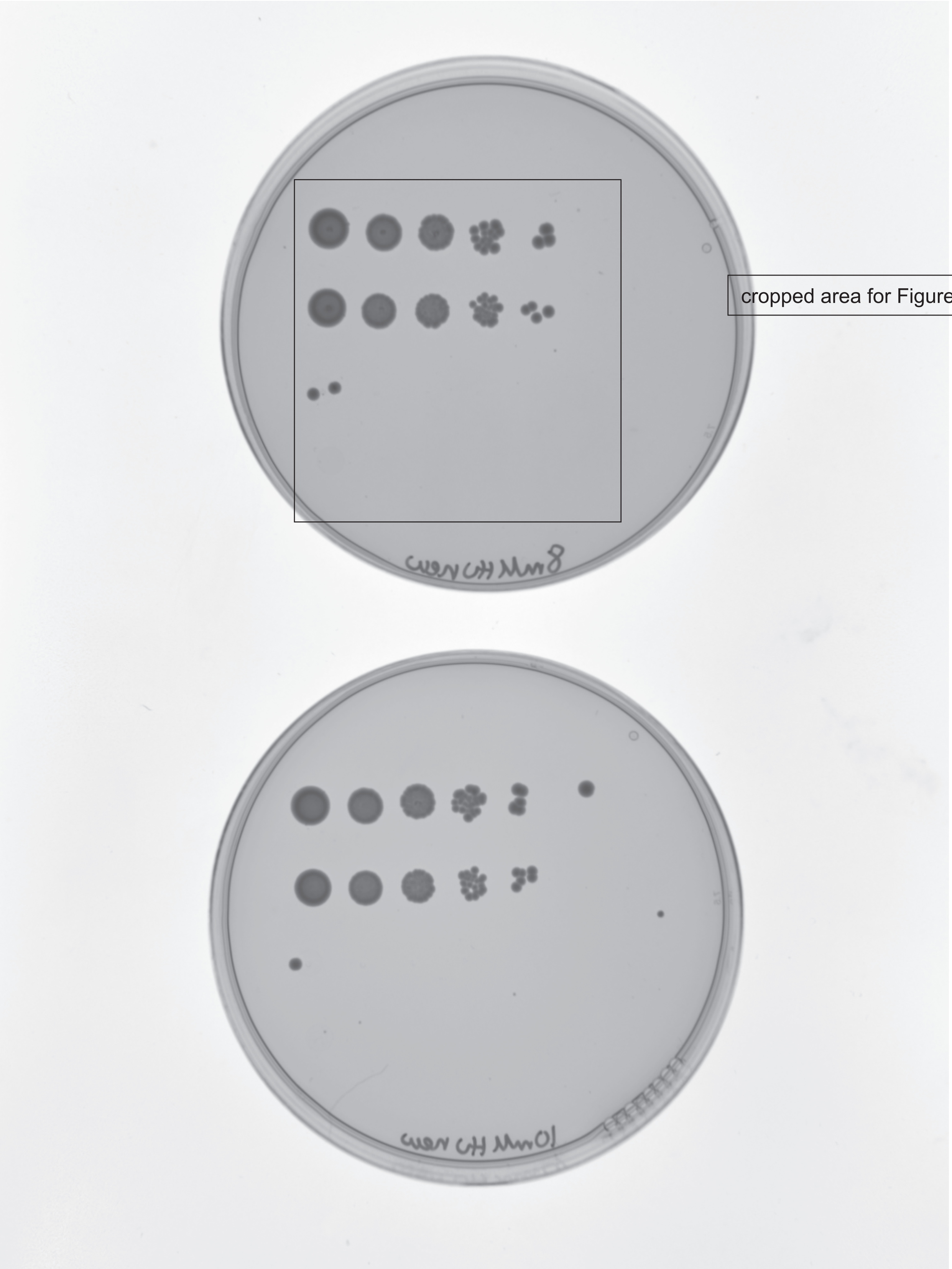

cropped area for Figure 7A top 8 mM HU

Figure 7 - source data 2.pdf
2048 x 2816

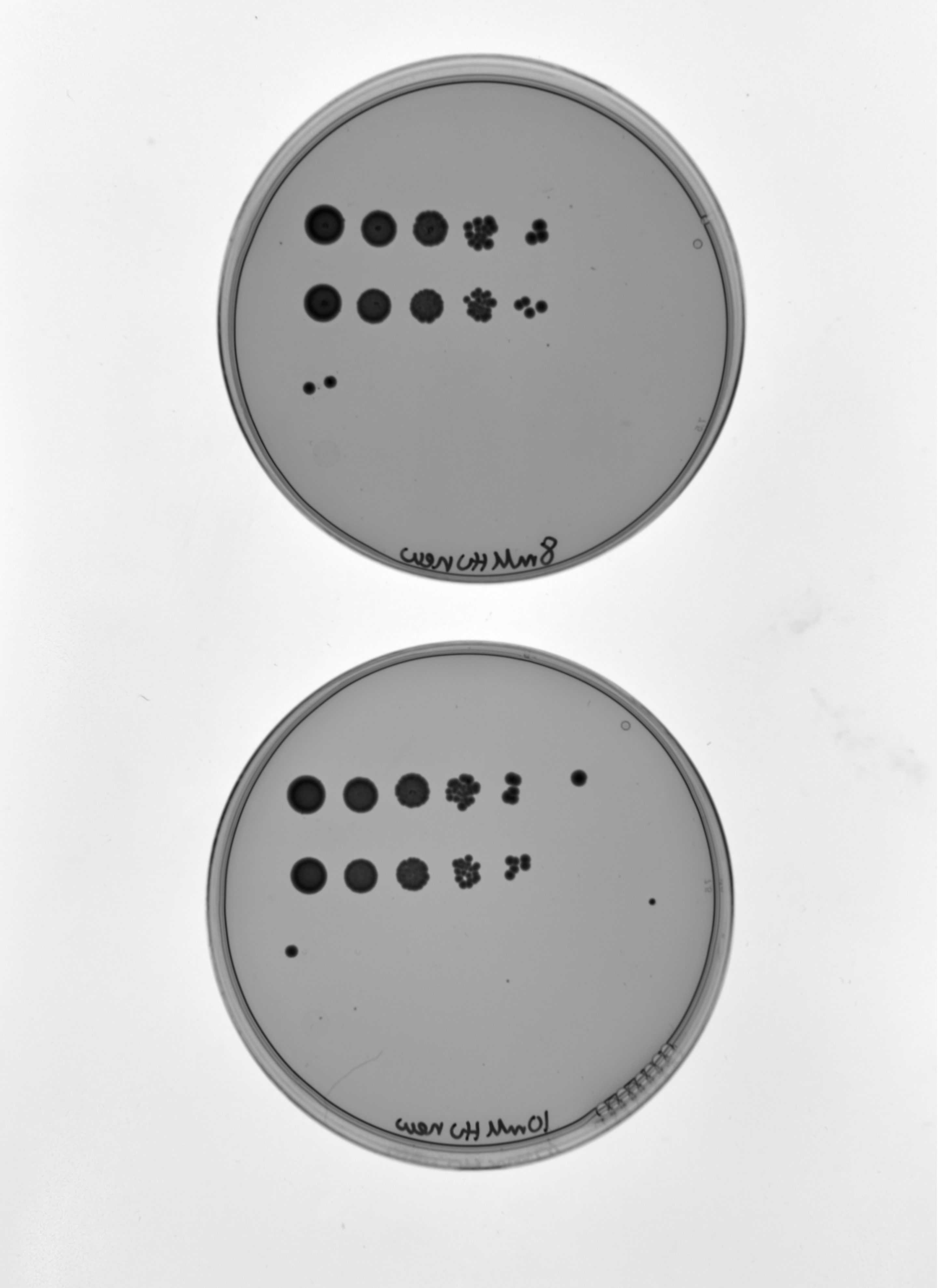
8mM HU new
10mM HU new

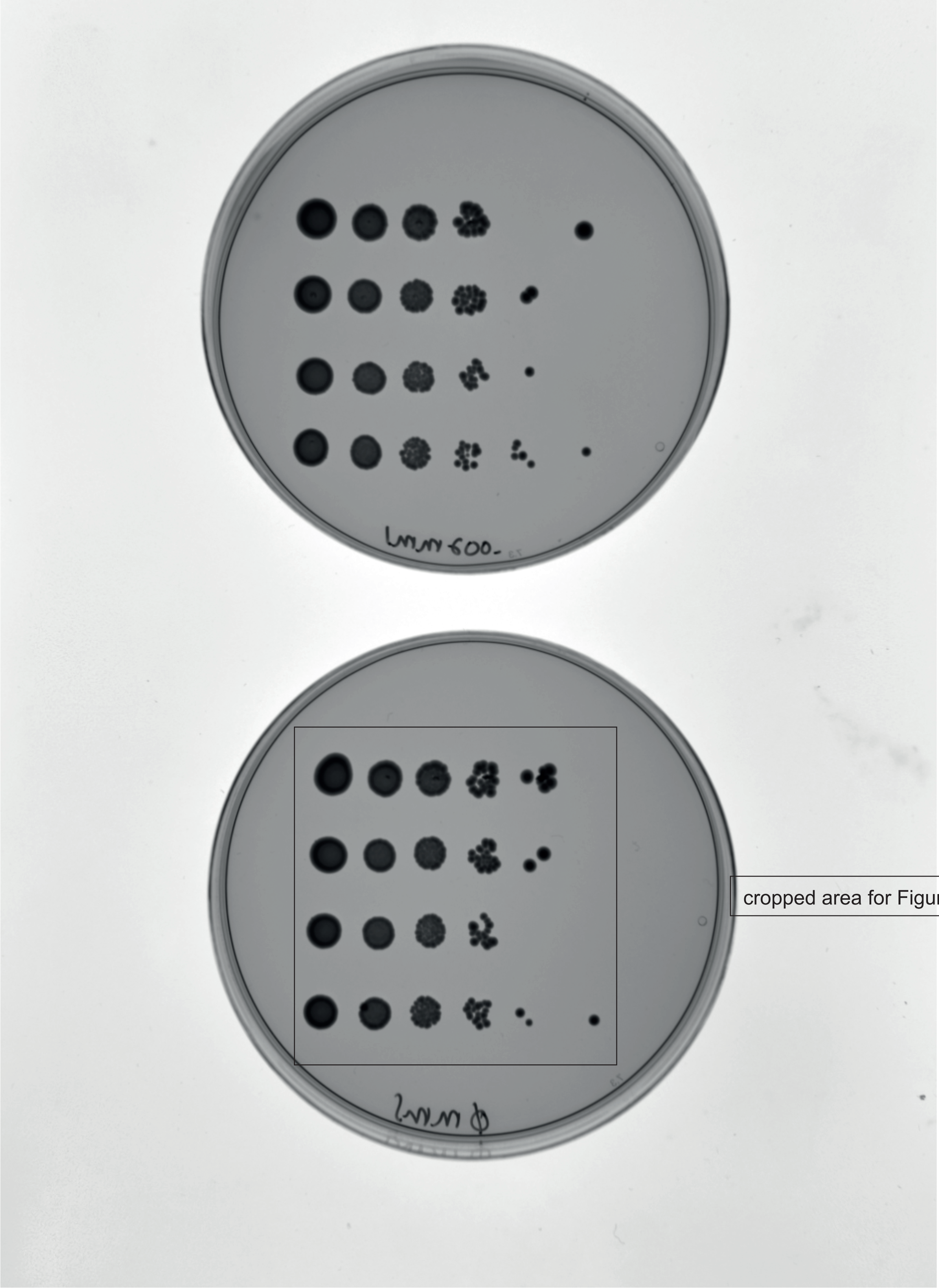

cropped area for Figure 7A bottom YPD

Figure 7 - source data 3.pdf

2048 x 2816

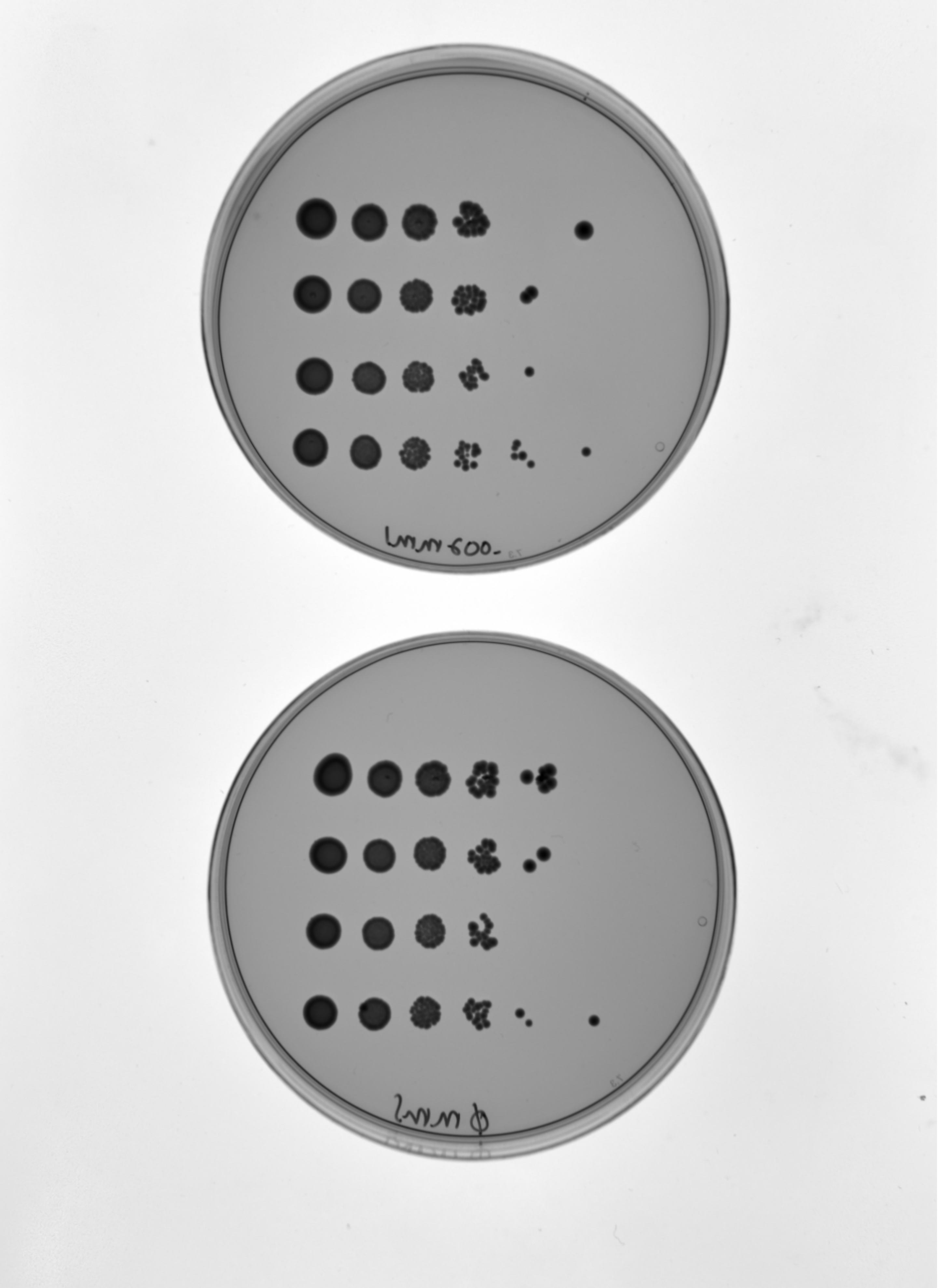

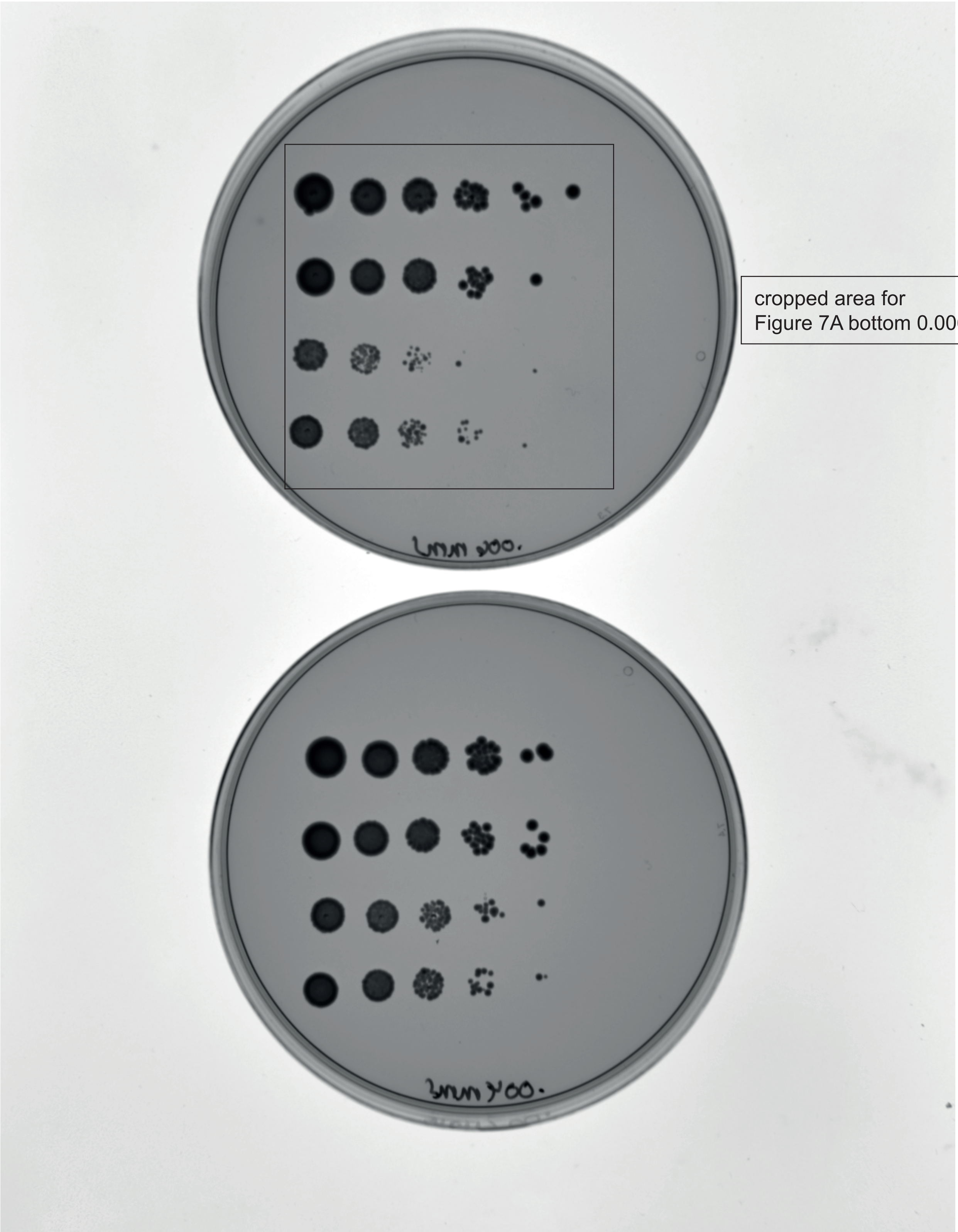

cropped area for
Figure 7A bottom 0.006% MMS

Figure 7 - source data 4.pdf

2048 x 2816

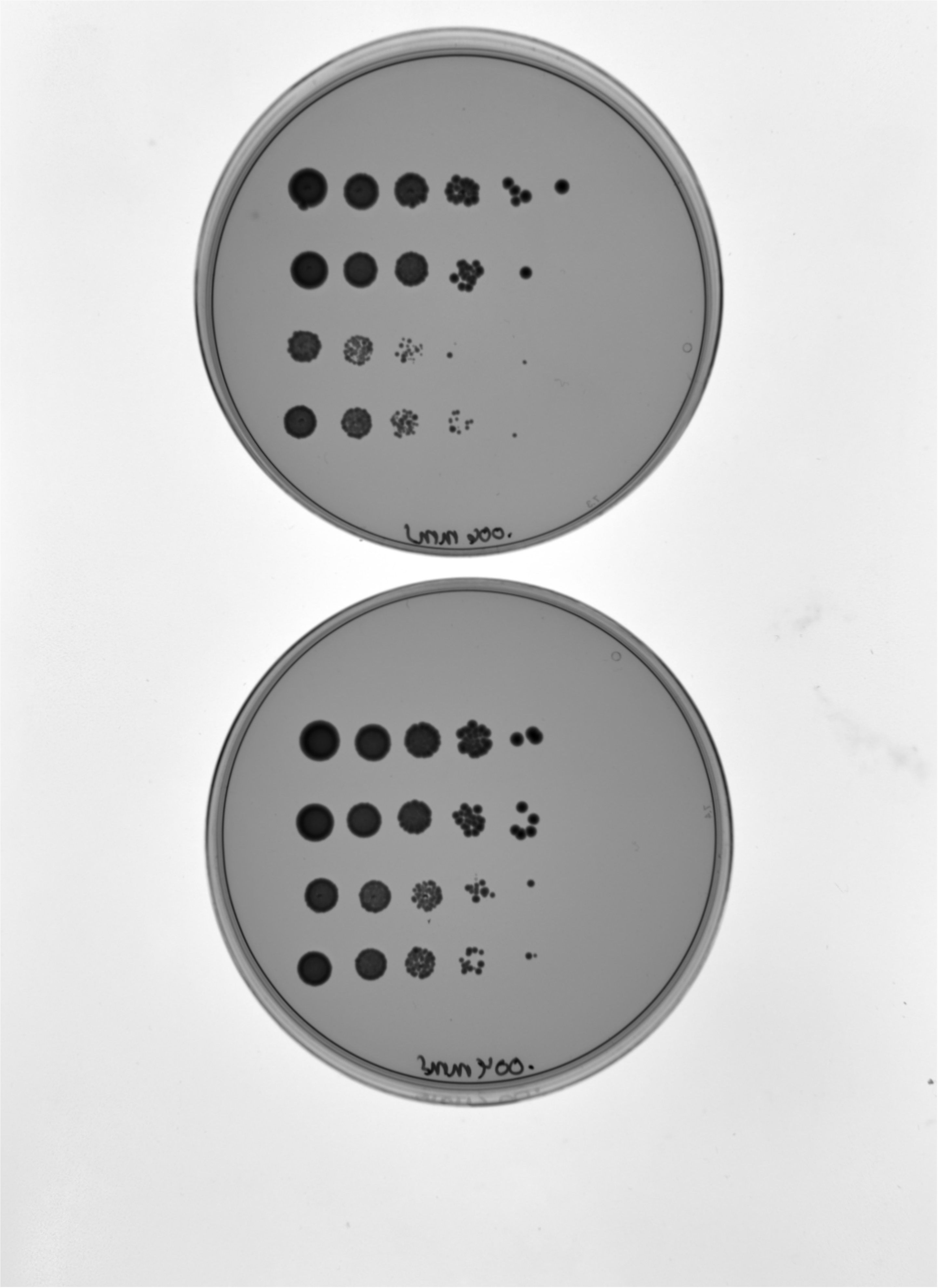

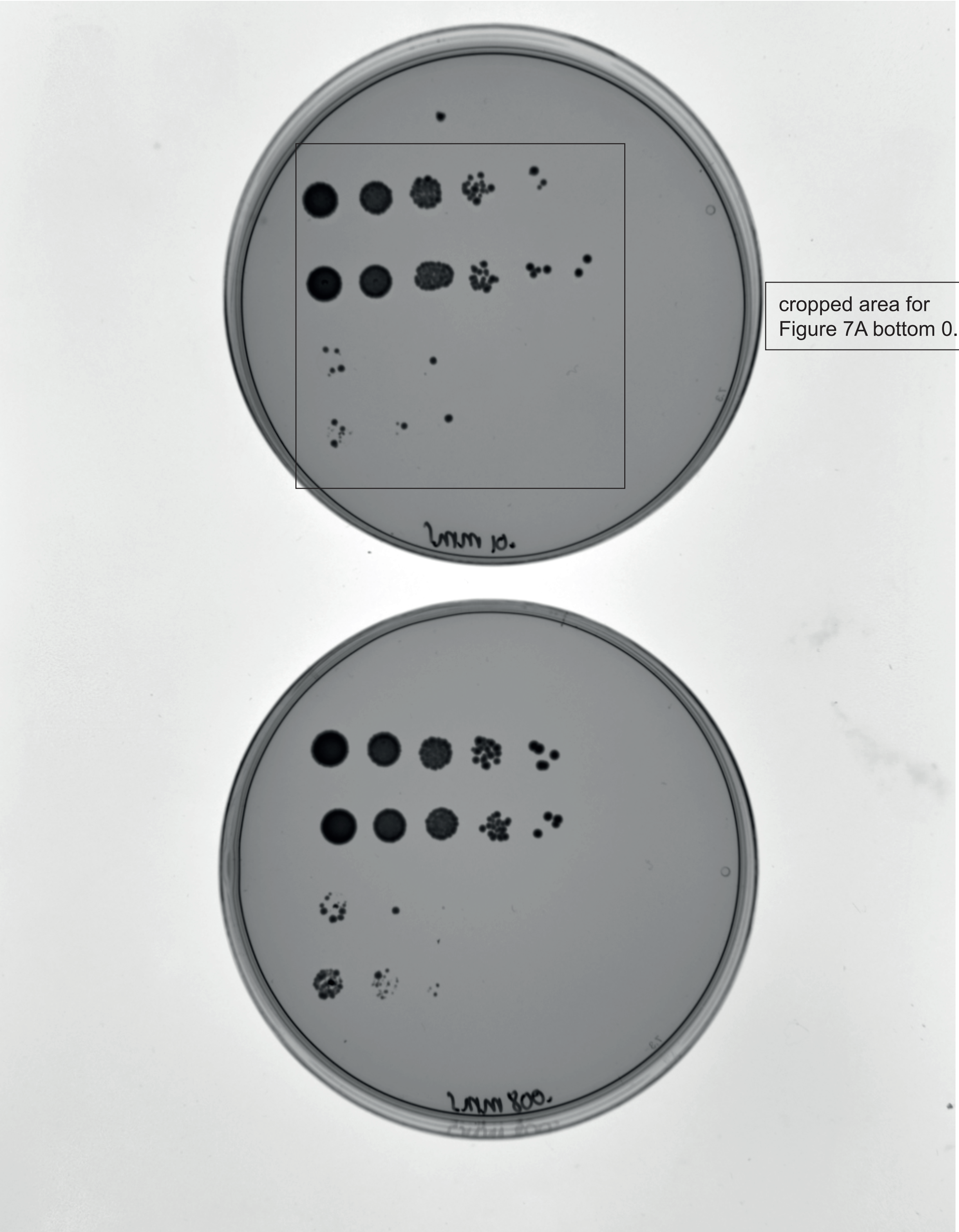

cropped area for
Figure 7A bottom 0.01% MMS

Figure 7 - source data 5.pdf

2048 x 2816

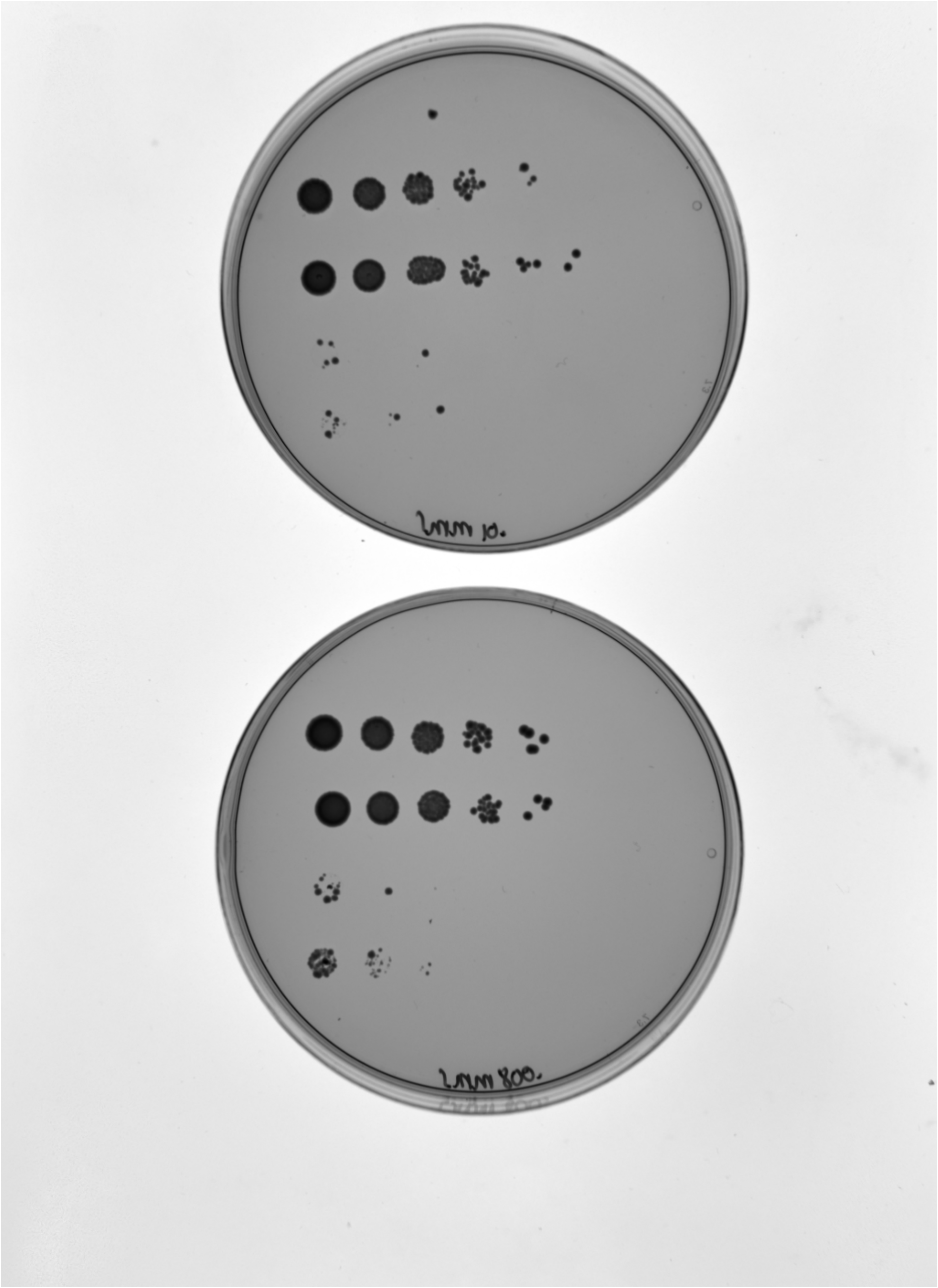

Supplement: Figure 7—source data 1. [file elife-69726-fig7-data1.pdf]
