# Supplementary material for: Rad53 checkpoint kinase regulation of DNA replication fork rate via Mrc1 phosphorylation
Source: eLife. 2021 Aug 13;10:e69726. doi: 10.7554/eLife.69726 (PMC8387023; doi:10.7554/eLife.69726)

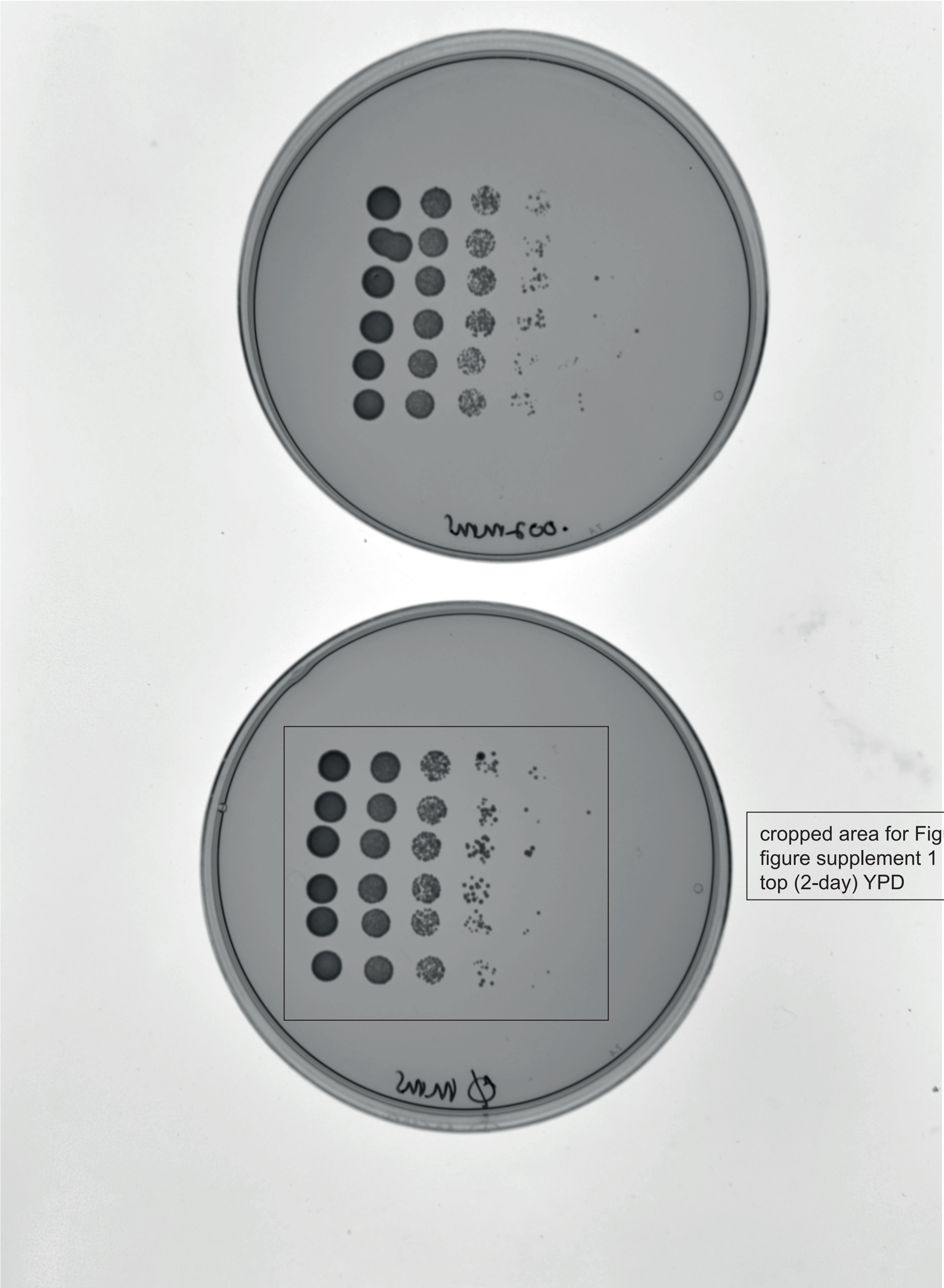

cropped area for Figure 7 -
figure supplement 1
top (2-day) YPD

Figure 7 - figure supplement 1 - source data 1.pdf

2048 x 2816

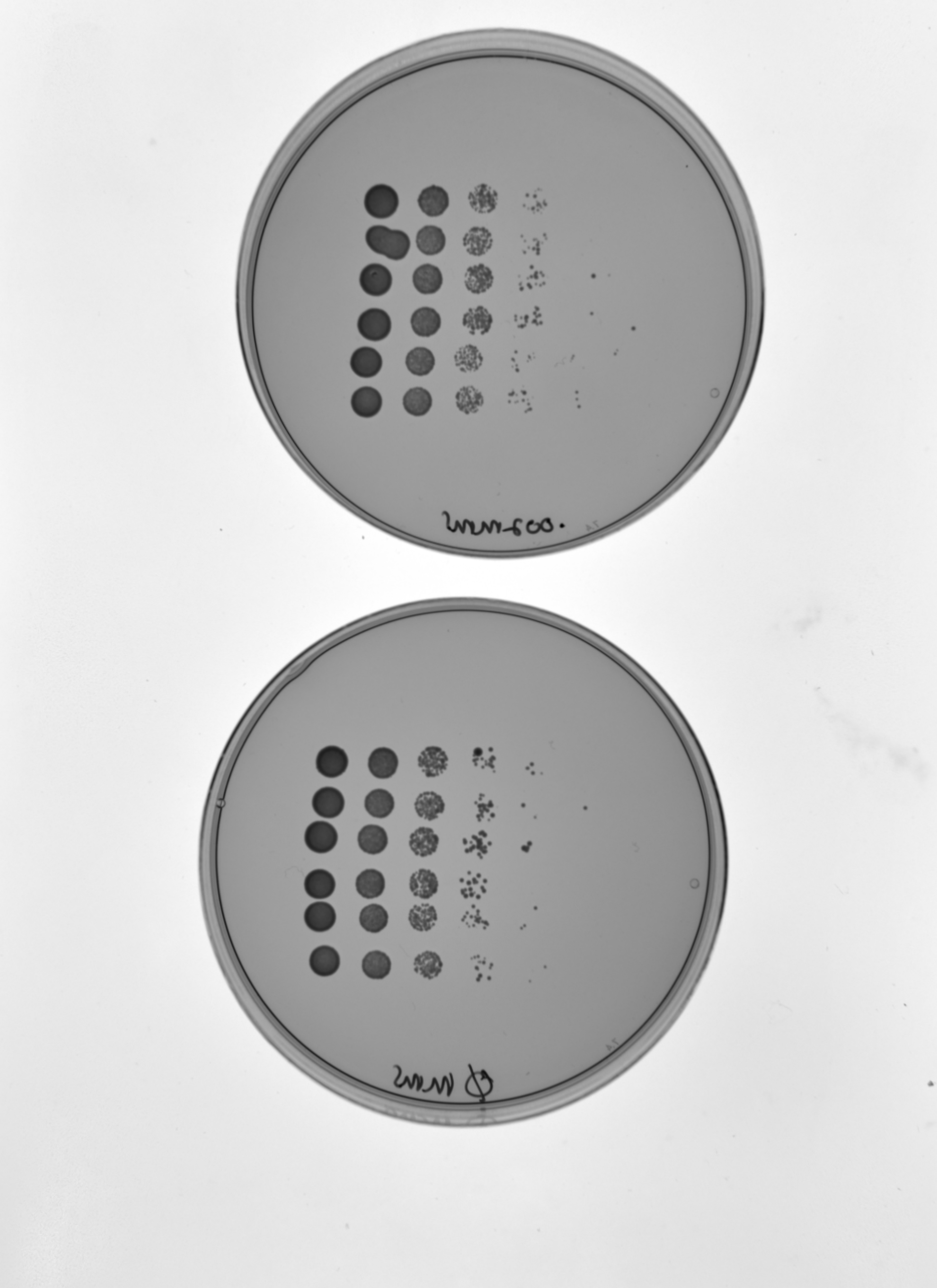

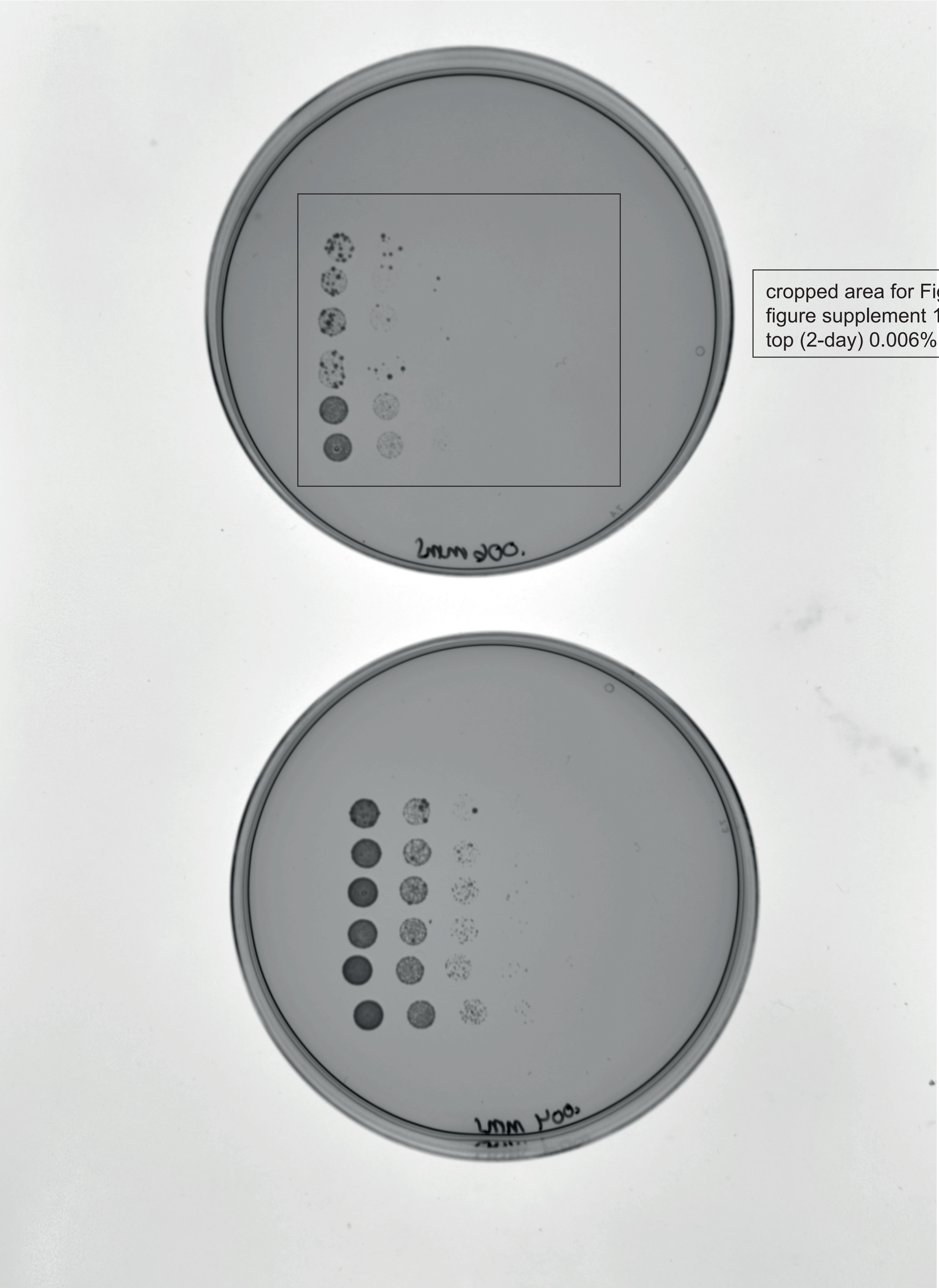

cropped area for Figure 7 -
figure supplement 1
top (2-day) 0.006% MMS

Figure 7 - figure supplement 1 - source data 2.pdf

2048 x 2816

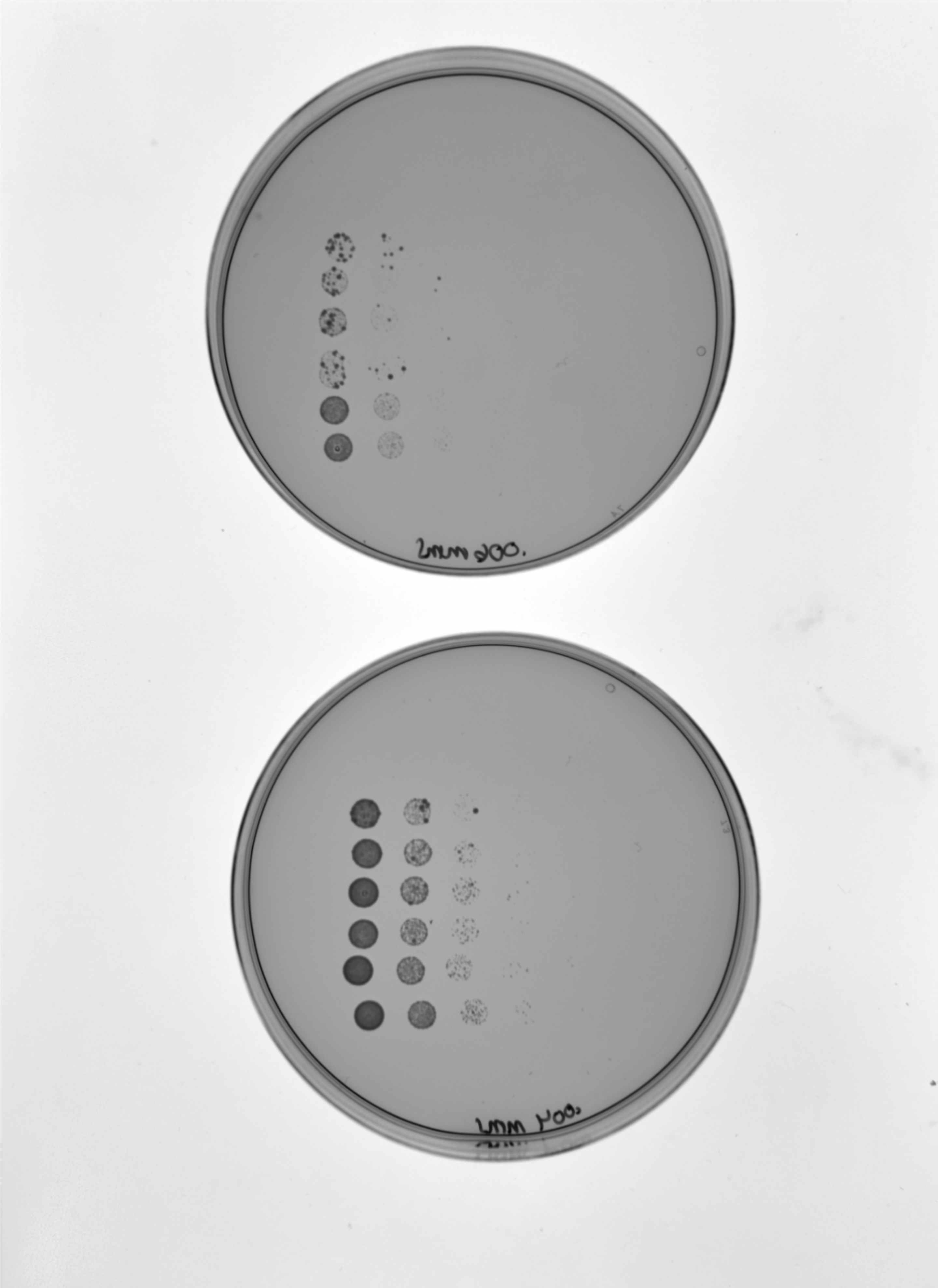

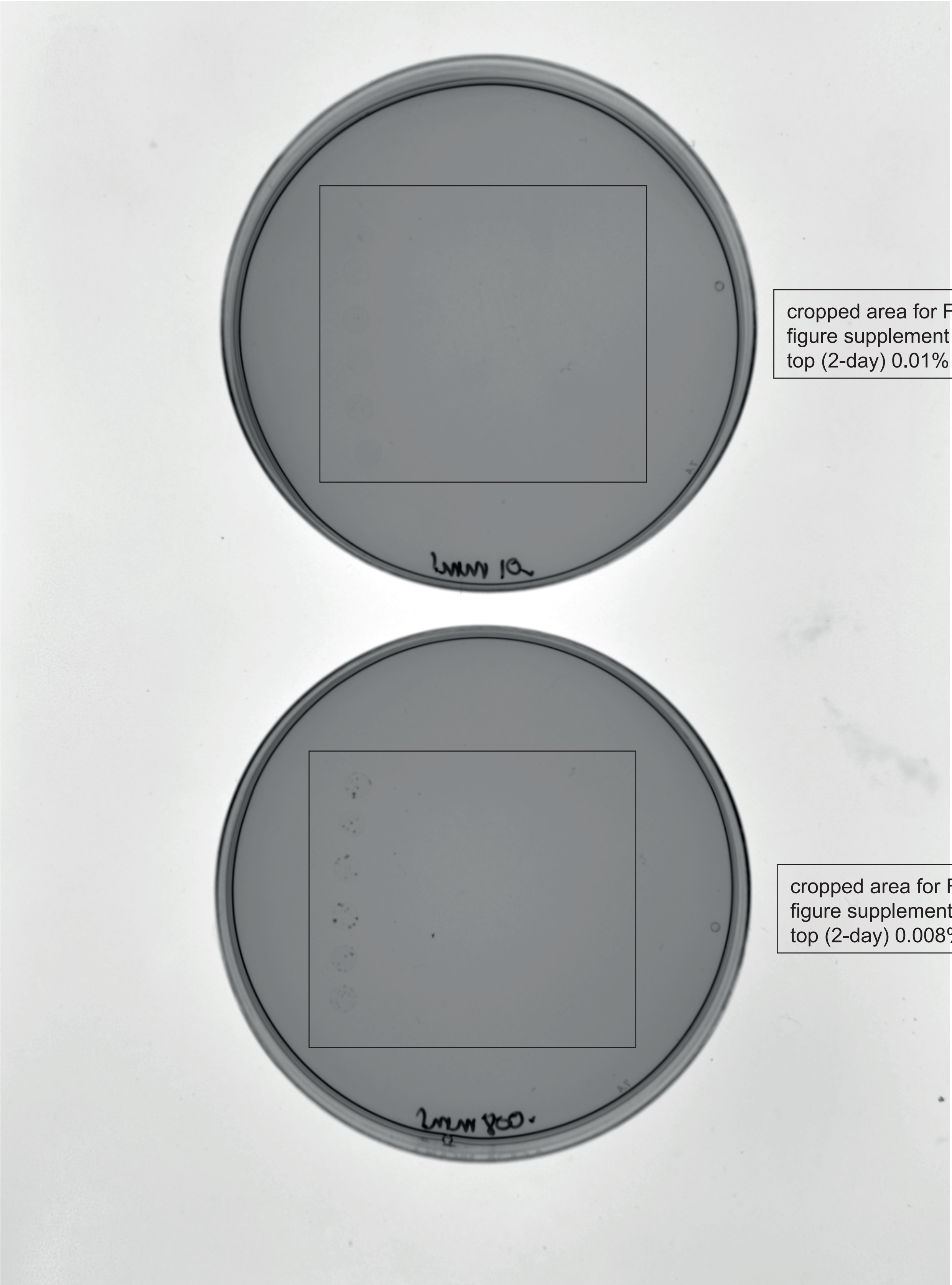

cropped area for Figure 7 - figure supplement 1
top (2-day) 0.01% MMS

cropped area for Figure 7 - figure supplement 1
top (2-day) 0.008% MMS

Figure 7 - figure supplement 1 - source data 3.pdf

2048 x 2816

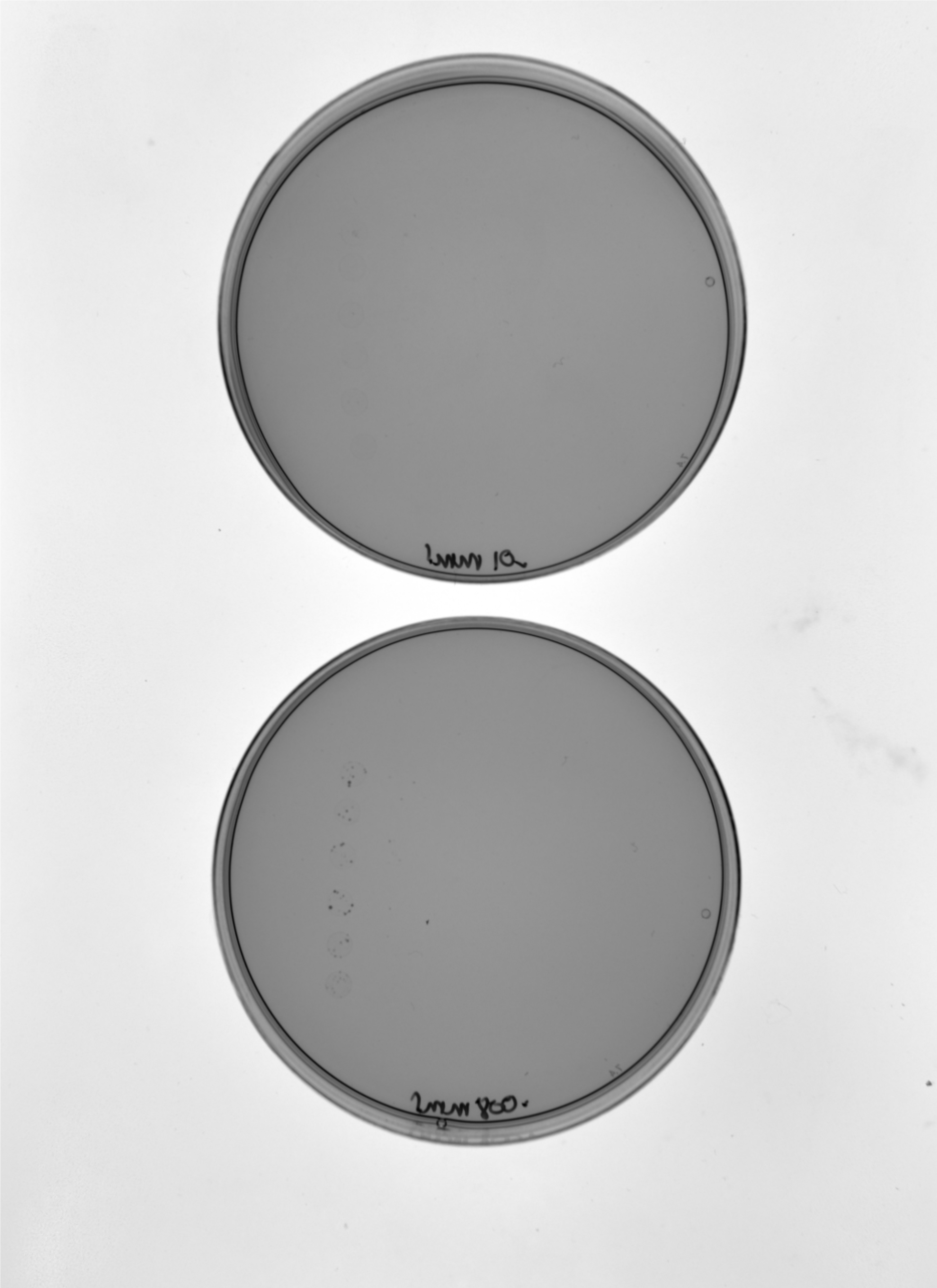

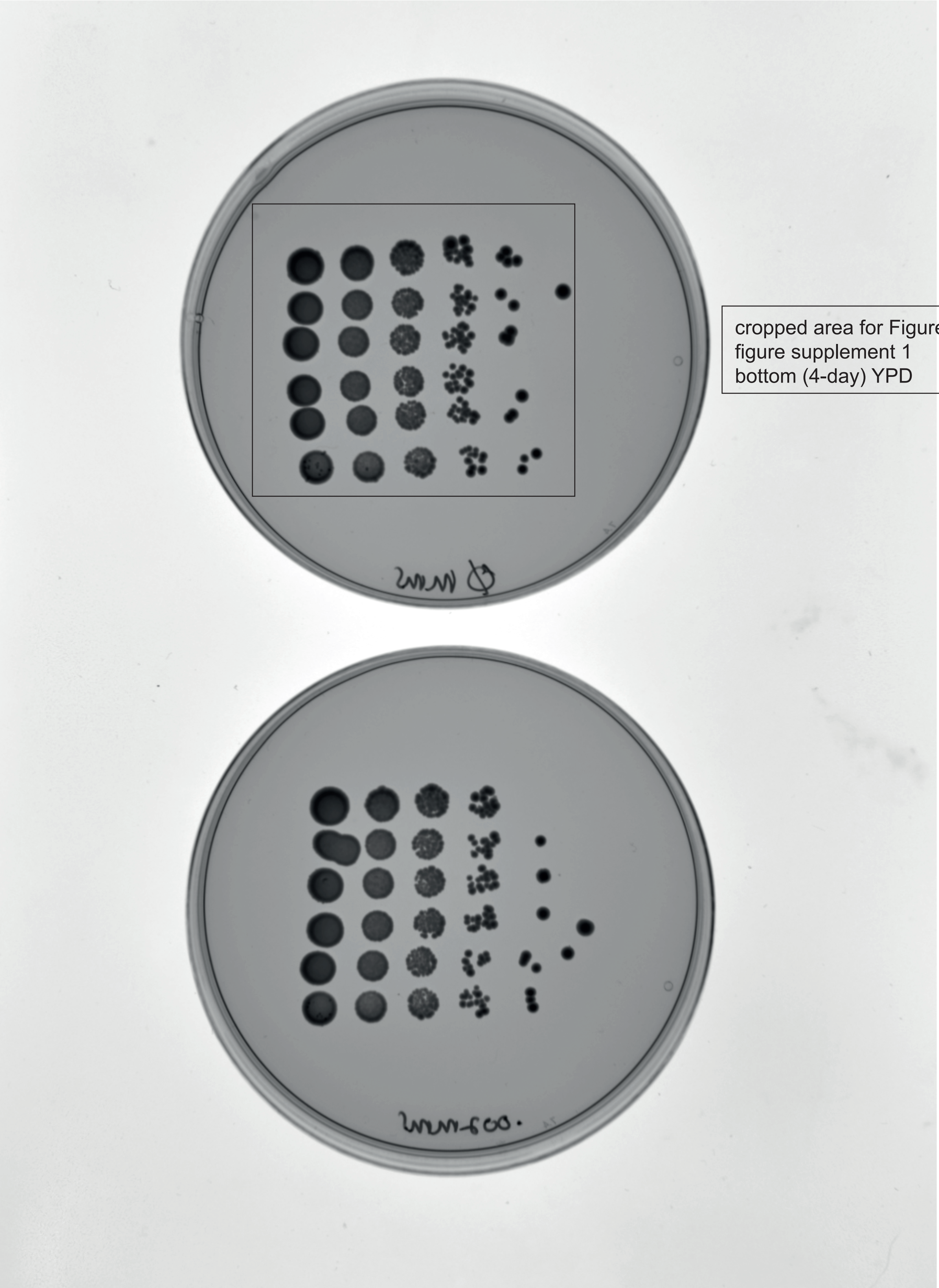

cropped area for Figure 7 - figure supplement 1 bottom (4-day) YPD

Figure 7 - figure supplement 1 - source data 4.pdf

2048 x 2816

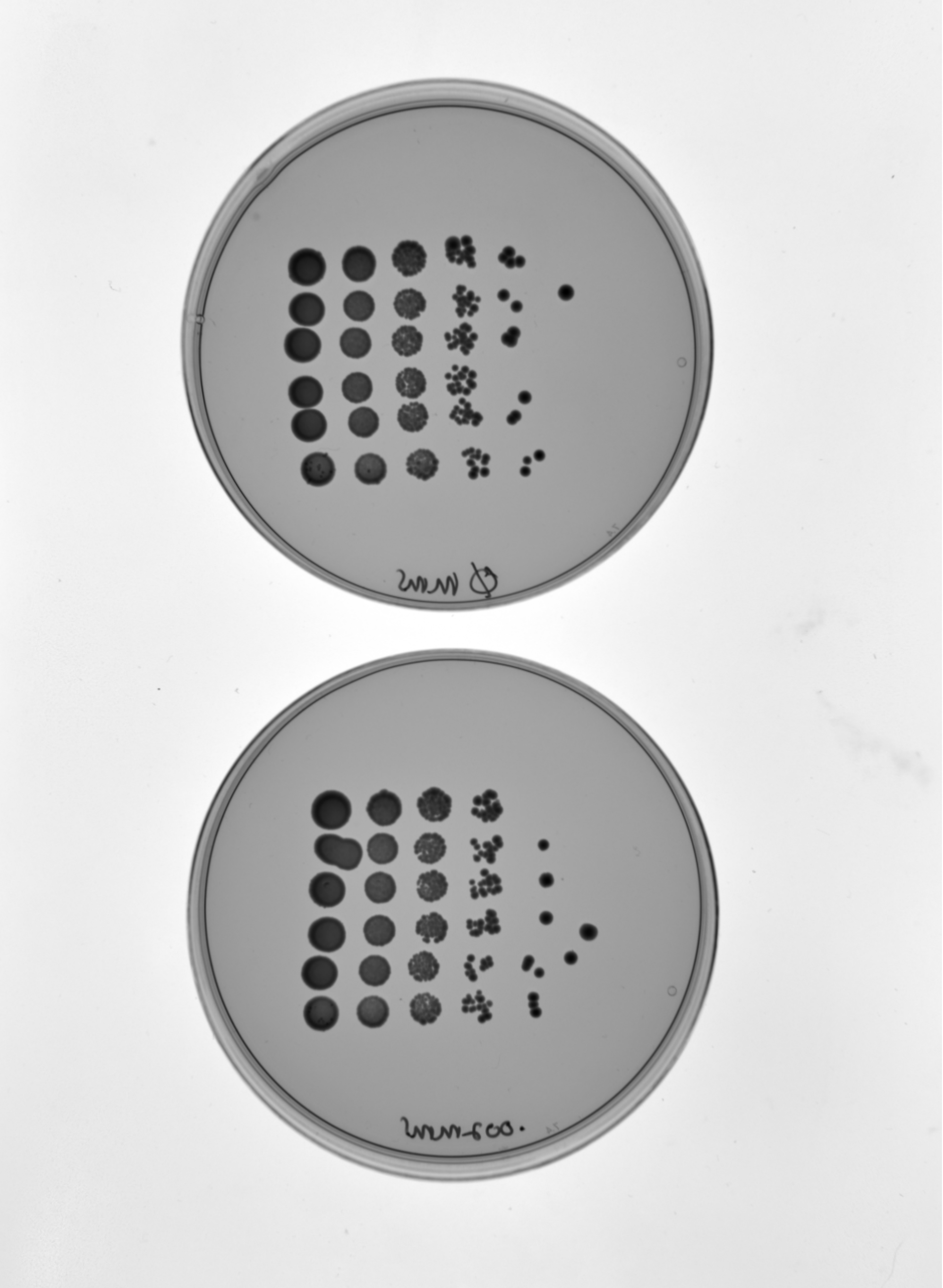

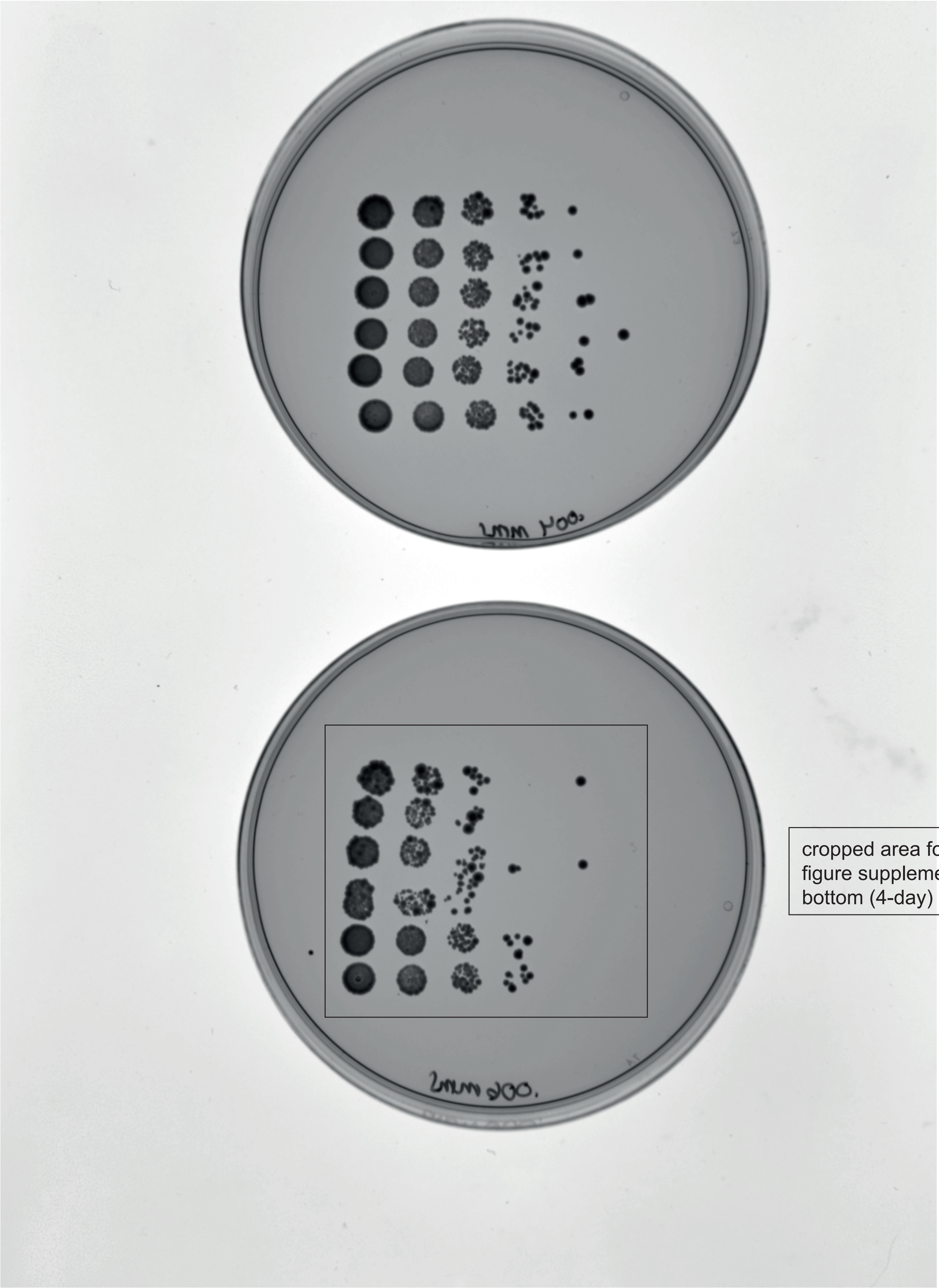

cropped area for Figure 7 -
figure supplement 1
bottom (4-day) 0.006% MMS

Figure 7 - figure supplement 1 - source data 5.pdf

2048 x 2816

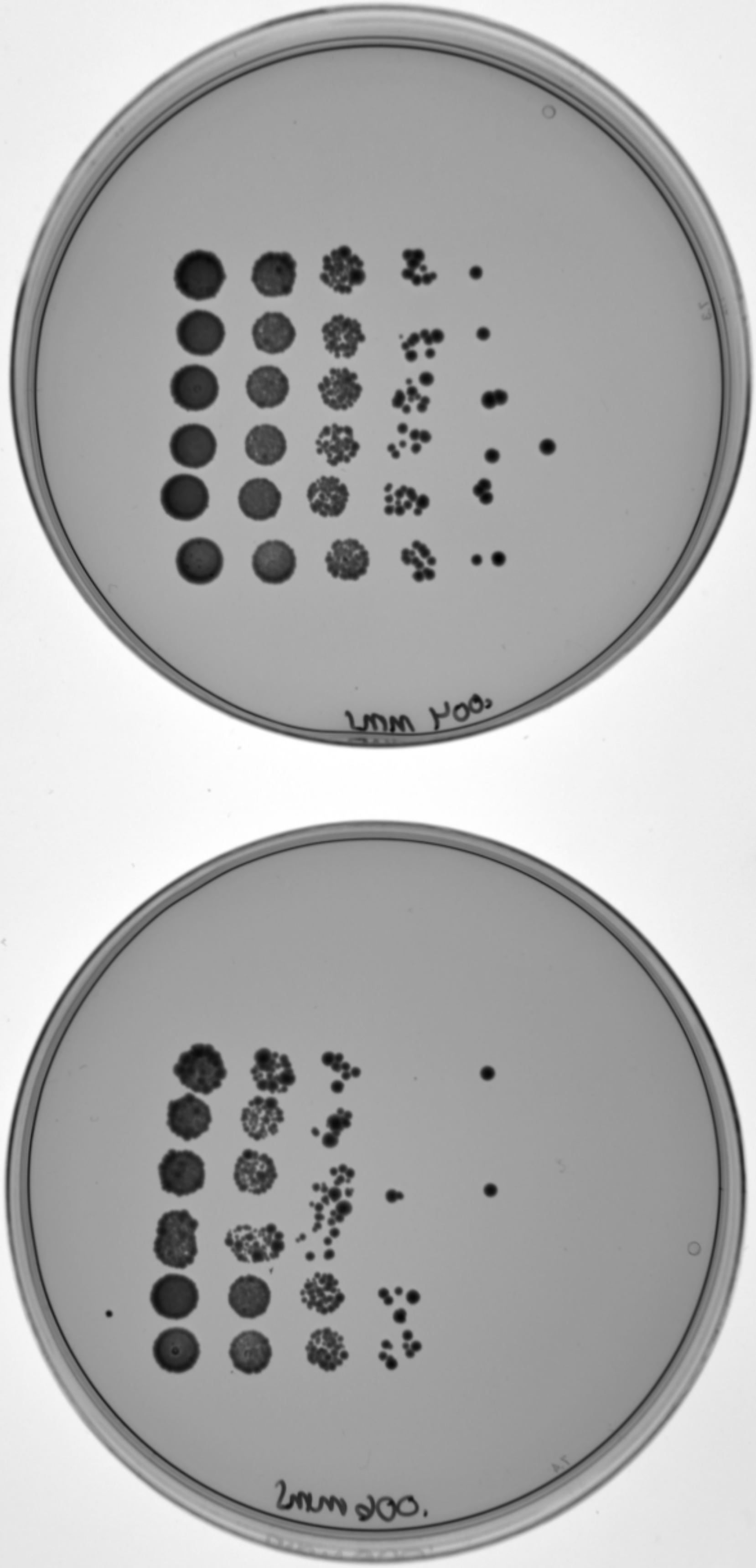
2mm 400.
2mm 200.

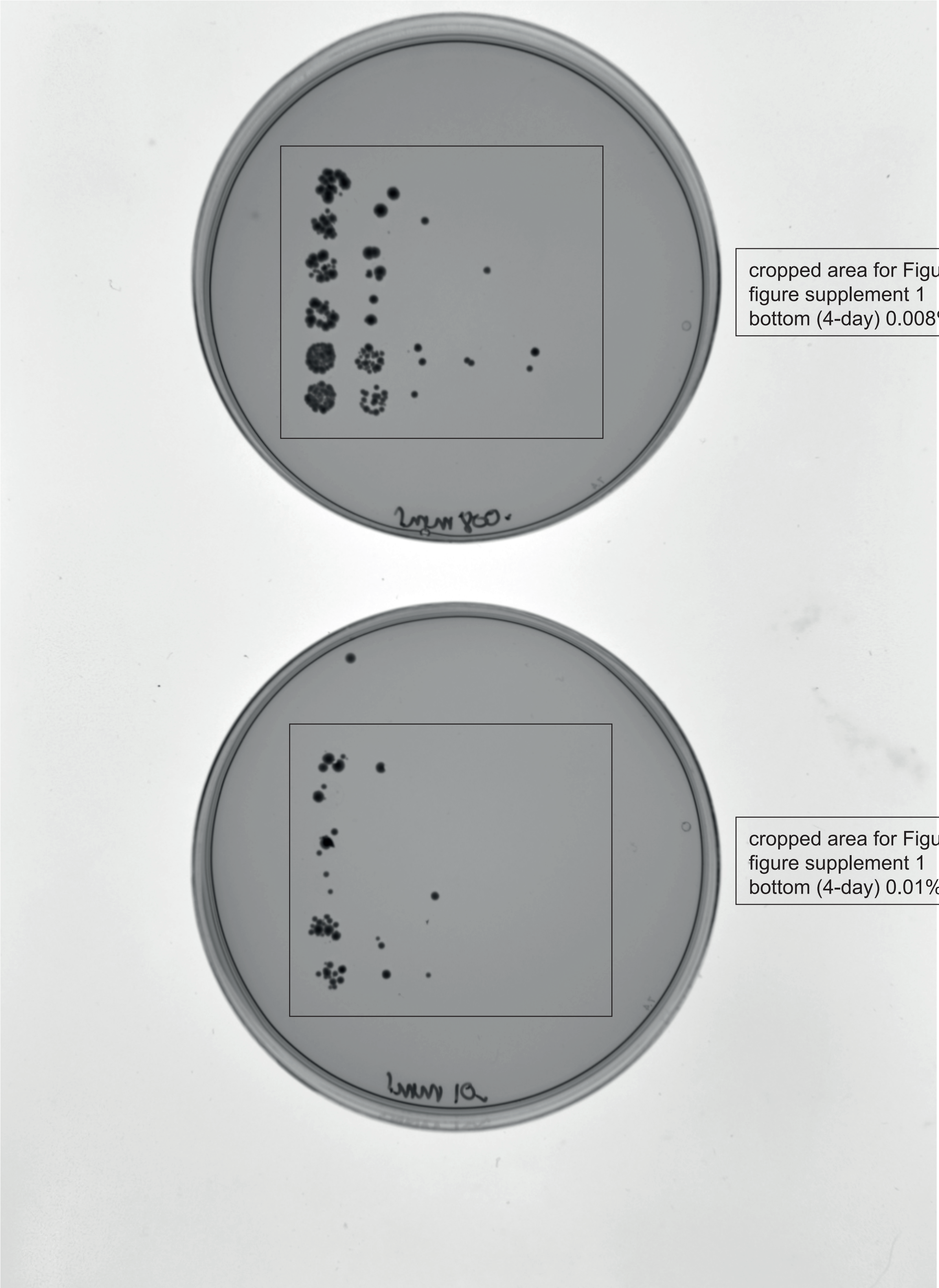

cropped area for Figure 7 -
figure supplement 1
bottom (4-day) 0.008% MMS

cropped area for Figure 7 -
figure supplement 1
bottom (4-day) 0.01% MMS

Figure 7 - figure supplement 1 - source data 6.pdf
2048 x 2816

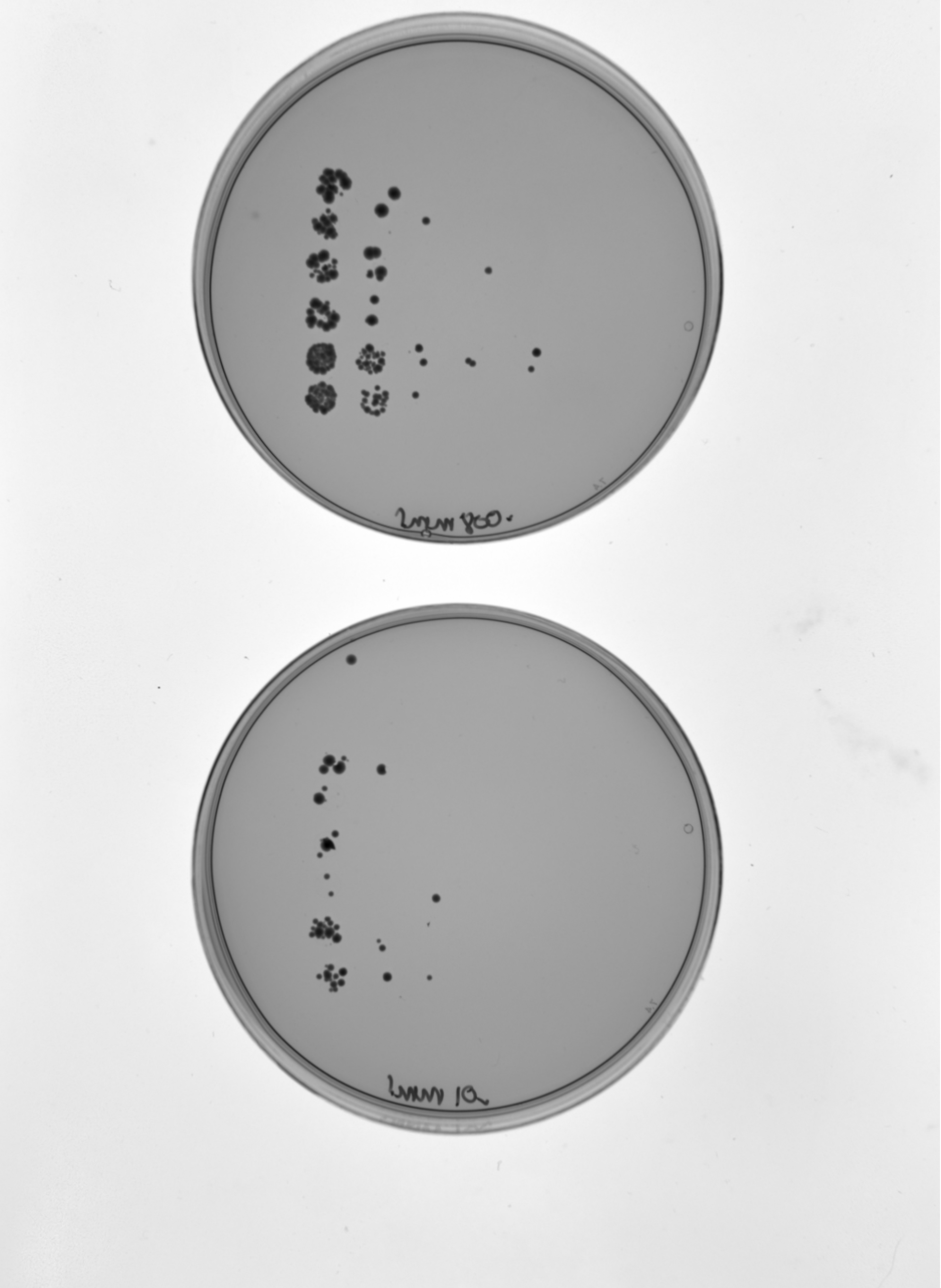

Supplement: Figure 7—figure supplement 1—source data 1. [file elife-69726-fig7-figsupp1-data1.pdf]
